# Beyond Structure: The Dynamic Role of the Extracellular Matrix Components in Immune Evasion

**DOI:** 10.34133/cancomm.0045

**Published:** 2026-09-24

**Authors:** Mohsin Maqbool, Anju Surendranath, Sadaf Khursheed Baba, Tooba Jawwad, Imadeldin Elfaki, Rashid Mir, Rakesh Kumar, Shahab Uddin, Mohammad Haris, Muzafar Ahmad Macha, Ammira Al-Shabeeb Akil, Mayank Singh, Sameer Mirza, Ajaz Ahmad Bhat

**Affiliations:** ^1^ Department of Cancer Biology and Pharmacology, Thomas Jefferson University, Philadelphia, PA, USA.; ^2^ Metabolic and Mendelian Disorders Clinical Research Program, Precision Omics Research and Translational Science, Sidra Medicine, Doha, Qatar.; ^3^ Department of Chemistry, College of Sciences, United Arab Emirates University, Al-Ain, United Arab Emirates.; ^4^ Department of Biochemistry, Faculty of Science, University of Tabuk, Tabuk, Saudi Arabia.; ^5^ Department of Medical Laboratory Technology, Prince Fahad Bin Sultan Chair for Biomedical Research, Faculty of Applied Medical Sciences, University of Tabuk, Tabuk, Saudi Arabia.; ^6^ School of Biotechnology, Shri Mata Vaishno Devi University, Katra, Jammu and Kashmir, India.; ^7^ Translational Research Institute, Academic Health System, Hamad Medical Corporation, Doha, Qatar.; ^8^ Center for Advanced Metabolic Imaging in Precision Medicine, Department of Radiology, Perelman School of Medicine, University of Pennsylvania, Philadelphia, PA, USA.; ^9^ Watson-Crick Centre for Molecular Medicine, Islamic University of Science and Technology, Pulwama, Jammu and Kashmir, India.; ^10^ Department of Medical Oncology, Dr. B.R. Ambedkar Institute Rotary Cancer Hospital, All India Institute of Medical Sciences, Ansari Nagar East, New Delhi, India.; ^11^ Zayed Bin Sultan Centre for Health Sciences, United Arab Emirates University, Abu Dhabi, UAE.

## Abstract

The extracellular matrix (ECM) is a dynamic and functionally active component of the tumor microenvironment that critically regulates immune cell trafficking, activation, and persistence. Rather than serving solely as a structural framework, ECM remodeling through collagen reorganization, proteoglycan-dependent chemokine sequestration, and fibroblast-driven matrix stiffening actively contributes to immune exclusion and evasion in solid tumors. This review integrates emerging mechanistic insights into how ECM composition, architecture, and mechanical properties shape spatial immune exclusion, T cell dysfunction, and immune-checkpoint regulation through mechanotransduction, metabolic reprogramming, and altered cytokine and chemokine signaling. Particular emphasis is placed on the coordinated activities of distinct ECM components and cancer-associated fibroblast subtypes in establishing spatially restricted immunosuppressive niches that limit antitumor immunity and therapeutic responsiveness. ECM-targeted therapeutic strategies are critically evaluated by integrating their mechanistic rationale, preclinical efficacy, clinical trial outcomes, and current stage of translational development. The importance of biomarker-guided patient stratification is also highlighted for identifying tumors most likely to benefit from ECM-directed interventions, particularly in combination with immune-checkpoint blockade and other immunotherapies. Finally, recent advances in spatial transcriptomics, proteomics, matrix imaging, and ECM-derived circulating biomarkers are discussed as tools to refine therapeutic targeting, monitor matrix remodeling, and predict treatment response. By conceptualizing the ECM as an active immunoregulatory network rather than a passive physical barrier, the review provides a mechanistic and translational framework for developing next-generation, ECM-informed cancer immunotherapies.

## Introduction

### Overview of the extracellular matrix and its components

**Fig. 1.**
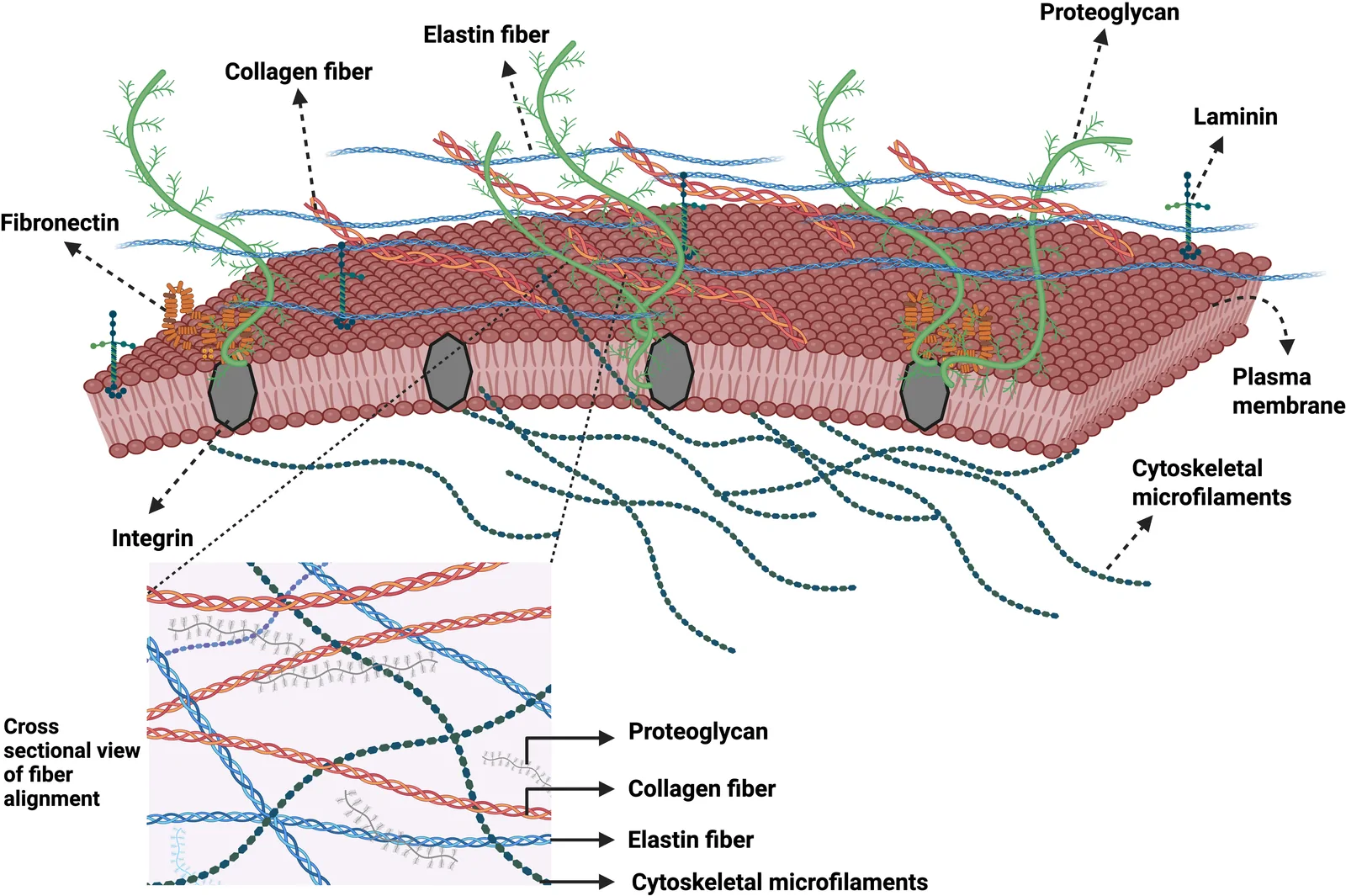
Schematic representation of the extracellular matrix and its molecular components. This figure illustrates the structural organization of the ECM and its key components involved in cell–cell and cell–matrix interactions. Integrins, transmembrane receptors embedded in the plasma membrane, are the primary link between the intracellular actin cytoskeleton and ECM proteins such as FN and collagen. Collagen fibers provide structural support and tensile strength to the ECM, helping maintain tissue integrity and architecture. FN connects integrins to collagen fibers in the ECM and to actin filaments in the cytosol, promoting cellular adhesion, migration, and signaling. Elastin is an ECM protein that provides tissues with elasticity and recoil, allowing them to stretch and return to their original shape. Laminin is a major basement membrane glycoprotein that supports cell adhesion, tissue organization, and cell signaling in the ECM. PGs modulate biochemical signaling between cells and the ECM, while cytoskeletal microfilaments support cell shape, vesicular transport, and mechanical integrity. ECM, extracellular matrix; PGs, proteoglycans; FN, fibronectin. The figure was created using BioRender.

The extracellular matrix (ECM) is a highly organized 3-dimensional (3D) network of connective tissue niches that support cells within tissues and provide a structural framework for the organization, remodeling, and regulation of cellular processes [1]. Beyond serving as a structural scaffold, the ECM plays a central role in regulating cell behavior, tissue architecture, and organ function, providing biochemical signals and biomechanical cues that govern cell adhesion, migration, proliferation, differentiation, and survival [2,3]. ECM comprises major components, such as matrix proteins, which provide cells and tissues with the essential support they need [3]. ECM proteins are classified into structural and nonstructural [4]. The structural proteins include collagen, elastin, fibronectin (FN), laminin, and other cell-binding glycoproteins. Nonstructural ECM components include matricellular proteins, such as tenascins (TNs), osteopontin, and thrombospondins, which primarily regulate cell–matrix interactions and cellular signaling [5]. Proteoglycans (PGs) are also important nonfibrillar components of the ECM and consist of one or more glycosaminoglycan (GAG) chains covalently attached to a defined core protein, with the notable exception of hyaluronic acid (HA), which lacks a protein backbone. Growth factors (GFs), integrins, and different subtypes of matrix metalloproteinases (MMPs) are the other crucial mediators involved in ECM regulation and signaling (Fig. 1). A diverse array of matrix proteins constitutes the main framework of the ECM, collectively forming the core matrisome [6] that comprises roughly 300 identified proteins [7].

Collagens are the principal load-bearing proteins of the ECM, constituting more than 30% of its overall protein content. Among these, fibrillar collagens I, II, and III collectively comprise approximately 80% to 90% of total body collagen, underscoring their central role in maintaining tissue architecture and biomechanical integrity [8]. To date, about 46 triple-helical collagen-α chains have been identified in vertebrates, which assemble into 28 distinct collagen types, each defined by the presence of one or more triple-helical domains [9]. It mainly accumulates in the ECM of connective tissues such as skin and tendons. Collagen is one of the most abundant proteins in the body, contributing to tensile strength and other cellular functions, such as adhesion and migration [10]. Almost 30 different forms of collagen have been identified; however, not all are exclusive to the ECM [11]. The most common type of collagen, type I, is widely distributed throughout the tissues, but is particularly abundant in bones, tendons, and the skin [12].

Elastin is the stretchy protein component of the ECM, whose function is closely related to that of collagen. The amino acid composition of elastin consists of a 75% abundance of glycine, proline, valine, and alanine residues, which imparts an overall hydrophobic property and acid/alkali resistance to elastin [7]. Elastin is a key determinant of ECM elasticity, enabling tissues and organs to stretch and recoil under mechanical stress. In addition to maintaining structural resilience, elastin shapes the cellular microenvironment by influencing adhesion, migration, and signal transduction [13]. It allows tissue to heal from prolonged strain in conjunction with glycoproteins, such as fibrillin and fibulin-5. It is essential for the functional integrity of tendons and other mechanically dynamic tissues, including arterial vessels, as it enables the characteristic crimped tendon architecture to extend under load and efficiently recoil upon release [7].

FN is a multifunctional glycoprotein that plays a central role in organizing the ECM and is vital to wound healing, cell adhesion, and ECM modeling [7]. It exists in 2 primary forms: the plasma FN, a soluble dimer synthesized mainly by hepatocytes and involved in wound healing and hemostasis, and cellular FN, an insoluble form produced by stromal cells and assembled into fibrillar networks within tissues [14]. Through integrin-mediated assembly into fibrillar networks, FN acts as a scaffold for collagen deposition and matrix alignment, thereby regulating ECM architecture and mechanics [15]. By binding integrins, GFs, and other ECM components, FN integrates biochemical and mechanical signals to control cell adhesion, migration, and survival, particularly during tissue remodeling and disease [7].

Laminins are a class of glycoproteins with a trimeric structure, composed of 3 disulfide-linked polypeptide chains: α, β, and γ, each with multiple genetic variants [16]. From a single heterotrimer in simpler organisms, laminins have evolved into over 16 distinct isoforms in more complex vertebrates, reflecting their diverse functional roles [17]. These proteins are expressed in various tissue types, including muscle, epithelial, and endothelial cells, and are pivotal in maintaining the structural integrity of the basement membrane (BM) [18]. Laminins are crucial for cell adhesion, influencing cell migration, differentiation, polarity, and survival. Through their interaction with integrins, they mediate signaling pathways that regulate tissue development, wound healing, and immune responses [19]. Furthermore, laminins are involved in pathological processes such as cancer metastasis and tissue fibrosis, highlighting their importance beyond normal cellular functions [17].

TNs constitute a family of ECM glycoproteins comprising 5 members: tenascin C (TNC) [20], tenascin R (TNR) [21], tenascin W (TNW) [22], tenascin X (TNX), and tenascin Y (TNY), which are predominantly expressed in connective tissues but are also present in specialized tissues such as the neural and cutaneous tissues [23]. TN is also expressed in stem cell niches during embryonic development [7].

In addition to providing structural support, the ECM serves as a reservoir for GFs and proteolytic enzymes, including MMPs. This reservoir function enables the ECM to integrate mechanical cues with GF signaling, thereby influencing cell fate decisions and tissue dynamics [24]. GFs, including transforming growth factor-β (TGF-β), fibroblast growth factor (FGF), and vascular endothelial growth factor (VEGF), interact with the ECM by binding to heparan sulfate (HS) or related heparan-based PGs. The activation of GFs is controlled by a range of tightly regulated molecular processes, including tissue remodeling, cellular proliferation, adhesion, migration, and cancer metastasis [25]. For instance, VEGF promotes the development of nerves and blood vessels [26]. MMPs constitute a major class of proteolytic enzymes that mediate ECM degradation. Numerous MMPs have been identified to date, and because of their “overlap in substrate specificity”, they are highly efficient at degrading the matrix [27]. Matrix degradation is a key process in ECM remodeling, occurring, for instance, during neovascularization, tissue regeneration, and tumor invasion, and is essential for the proper functioning of the ECM in many tissues [28]. MMPs are activated when tissue healing increases cytokine and GF activity [7,29].

### Functions of the ECM

The ECM performs various tasks, mainly tissue organization, remodeling, morphogenesis, and regulating intercellular communication. The primary role of the ECM is to provide cells with an anchoring substrate, which is essential for maintaining cell polarity and division [30]. ECM is now recognized as a material that gives cells biophysical and biochemical cues and serves as a scaffold for cell formation [31]. The ECM plays a fundamental role in maintaining the structural integrity of tissues. Collagen is the main protein component that provides the ECM with mechanical stability and tensile strength [32]. Moreover, the ECM serves as a physical scaffold that facilitates cellular adhesion via cytoskeletal coupling, thereby enabling extracellular communication and cell migration. Several cell adhesion molecules, such as integrins, immunoglobulin (Ig) family, selectins, and cadherins, often facilitate this process [33], which will activate several signal transduction mechanisms that promote immune responses, cell recruitment, tissue differentiation, and regeneration [34]. The ECM directs cell migration by modulating cell polarity, morphology, and matrix remodeling. At the same time, the spatial organization of adhesion sites and adhesion molecules such as integrins and cadherins guides directional movement [35]. On the contrary, cancer cells exhibit a strong tendency to dynamically alter the ECM to promote invasive cell movement by releasing matrix-degrading enzymes such as MMPs [28].

In an early study by Kessler et al. [36], it was evidenced that the human dermal fibroblasts cultured within a 3D network of fibrillar collagen led to the systematic identification of mechanoresponsive genes that code for ECM glycoproteins, along with protease inhibitors, fibrogenic GFs, and focal adhesion components. By sequestering GFs and bioactive ligands, the ECM enables their controlled release, thereby shaping cell signaling and phenotypic outcomes [37,38]. The physical characteristics of the ECM, such as its topography, elasticity, and rigidity, also play a substantial role in shaping cell growth and specialization, ultimately influencing the structure and stability of tissues [39].

While physiological ECM stiffness maintains tissue strength and structural coherence, aberrant matrix stiffening activates mechanotransduction pathways in tumor cells, promoting cytoskeletal remodeling, pseudopodia extension, and enhanced integrin-mediated adhesion. These stiffness-induced changes increase interstitial fluid pressure, compromising vascular perfusion and generating nutrient or oxygen-deprived microenvironments that favor tumor progression. For instance, this stiffening may activate ion channels, such as piezo-type mechanosensitive ion channel component 1 (PIEZO1) and transient receptor potential vanilloid 4 (TRPV4), as well as integrin-like receptors to modulate cancer hallmarks, such as inflammation and angiogenesis, thereby facilitating the malignant phenotype of tumors [40].

### Introduction to immune escape mechanisms in cancer

The ECM not only supports tissue integrity but also plays an essential role in tumor biology by influencing tumor growth, metastasis, and the response to therapies. As the ECM remodels during tumor progression, it alters the tumor microenvironment (TME) and interacts with immune cells, facilitating or hindering immune responses [41]. The immune system is a crucial component of cancer therapy, as it serves both as a potential defense mechanism against tumors and as a regulator of tumor biology. Tumor immunotherapy leverages the host immune system’s ability to restore its capacity to mount an effective defense against malignant cells, including inducing an antitumor immune response while preventing immune attacks on healthy tissue. The immune system, therefore, suppresses or promotes tumor growth, invasion, metastasis, and overall tumor progression [42].

Tumor cells utilize multiple overlapping immune evasion strategies to escape immune surveillance [42]. A key approach is the up-regulation of immune-checkpoint proteins such as programmed death ligand 1 (PD-L1) [43] on both tumor and stromal cells, and cytotoxic T lymphocyte-associated antigen 4 (CTLA-4) [44] on T cells. These checkpoints inhibit the activation, proliferation, and effector functions of cytotoxic T cells; specifically, the binding of PD-L1 to programmed cell death protein-1 (PD-1) on cytotoxic T cells triggers the PD-L1/PD-1 pathway, promoting T cell exhaustion and apoptosis [45]. Similarly, the interaction of CTLA-4 with cluster of differentiation 80/86 (CD80/CD86) on antigen-presenting cells (APCs) dampens T cell activation, further impeding an effective immune response [46]. These immune checkpoints have become key targets for immunotherapies designed to block these inhibitory signals and enhance T cell responses against tumors [46].

**Fig. 2.**
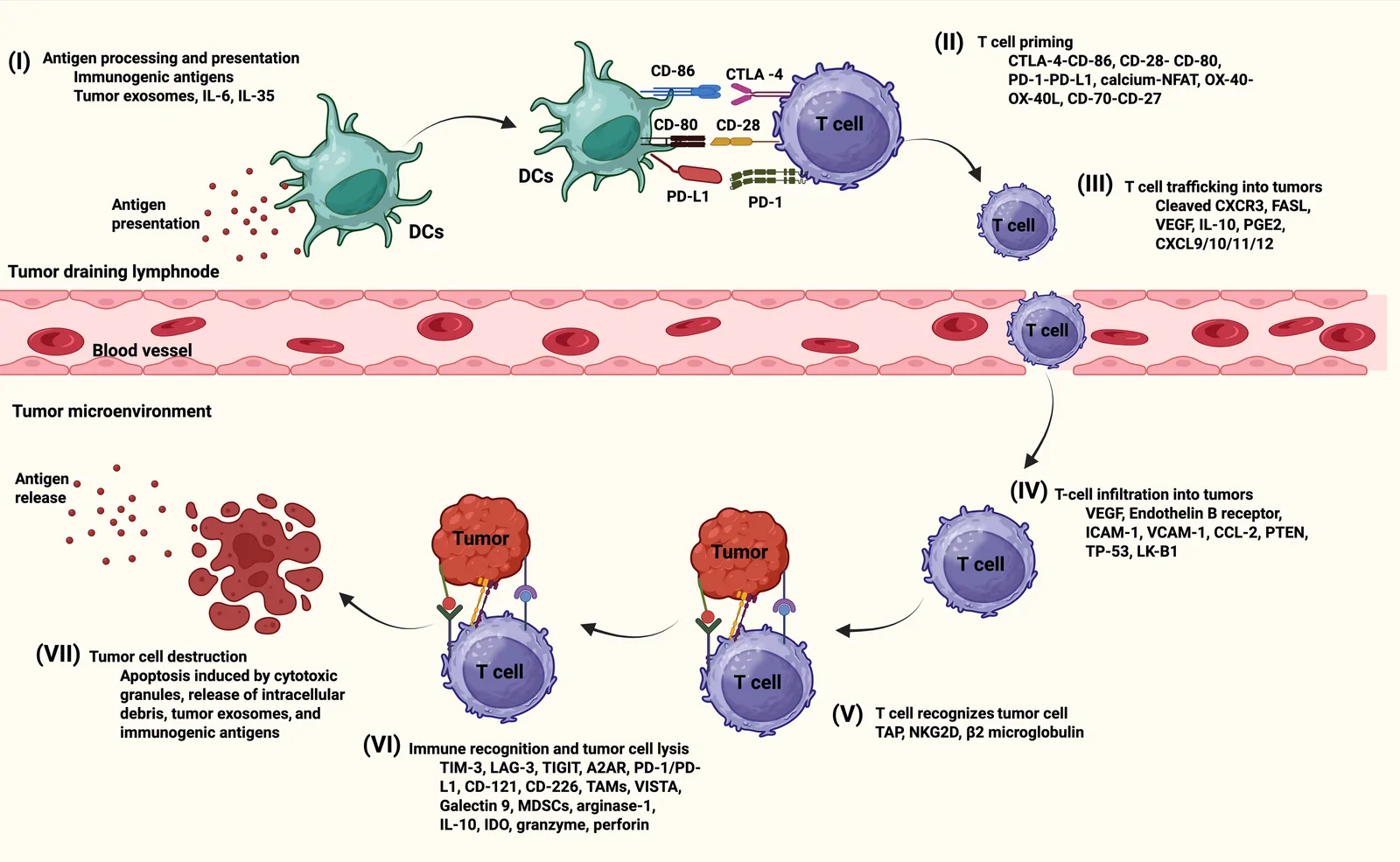
Sequential steps regulating T cell-mediated antitumor immunity and immune escape in the tumor microenvironment. The figure illustrates the major stages required for effective T cell-mediated antitumor immunity, beginning with antigen release and DC activation in the tumor-draining lymph node and progressing through T cell priming, trafficking, infiltration, tumor recognition, immune-mediated lysis, and tumor-cell destruction. Each stage can be regulated or disrupted by tumor-derived factors, immune-checkpoint pathways, stromal barriers, and suppressive immune cell populations. (I) Antigen processing and presentation: DCs process and present tumor-derived immunogenic antigens to T cells, initiating adaptive antitumor immunity, while exosomes and cytokines such as IL-6 and IL-35 mainly modulate DC function and the immune response. (II) T cell priming: Antigen-presenting DCs interact with T cells through costimulatory and co-inhibitory receptor–ligand pairs. The figure highlights CD86–CTLA-4, CD28–CD80, PD-L1–PD-1, OX-40–OX-40L, CD70–CD27, and calcium–NFAT interactions that determine whether T cells become effectively activated or are restrained by inhibitory checkpoint signaling. (III) T cell trafficking to tumors: Activated T cells exit the tumor-draining lymph node and traffic through the blood vessel toward the TME. This process is influenced by chemokines, cytokines, and tumor-derived mediators, including cleaved CXCR3, FASL, VEGF, IL-10, PGE2, and CXCL9/10/11/12, which regulate T cell migration, survival, and recruitment to tumor sites. (IV) T cell infiltration into tumors: After reaching the tumor vasculature, T cells must extravasate and infiltrate the tumor tissue. This step is shaped by vascular and stromal factors, including VEGF, endothelin B receptor, ICAM-1, VCAM-1, CCL-2, PTEN, TP-53, and LK-B1, which can either support or restrict immune-cell entry into the TME. (V) T cell recognition of tumor cells: Within the tumor, T cells recognize malignant cells through antigen-presentation machinery and activate immune-recognition pathways. Molecules shown include TAP, β2 microglobulin, and NKG2D, which contribute to antigen processing, major histocompatibility complex class I presentation, and cytotoxic lymphocyte recognition of tumor cells. (VI) Immune recognition and tumor-cell lysis: Following recognition, T cells engage tumor cells and induce cytotoxic killing. This stage is regulated by immune checkpoints and suppressive pathways, including TIM-3, LAG-3, TIGIT, A2AR, PD-1/PD-L1, CD121, CD226, VISTA, Galectin 9, IDO, IL-10, Arginase-1, TAMs, and MDSCs. Effector molecules such as granzyme and perforin mediate tumor-cell lysis, whereas suppressive mediators and cells can reduce cytotoxic efficacy. (VII) Tumor-cell destruction: Successful cytotoxic T cell activity results in tumor-cell apoptosis, the release of intracellular debris, tumor exosomes, and the presentation of additional immunogenic antigens. These products can feed back into antigen processing and presentation, thereby sustaining or amplifying the cancer–immunity cycle. Overall, the figure depicts the cancer–immunity cycle as a coordinated process involving antigen release, DC-mediated presentation, T cell priming, vascular trafficking, tumor infiltration, antigen recognition, and cytotoxic destruction. It also highlights key immune-evasion mechanisms that may interrupt this cycle, including defective antigen presentation, immune-checkpoint activation, impaired trafficking and infiltration, suppressive cytokines, TAM- and MDSC-mediated inhibition, and reduced cytotoxic effector function. A2AR, adenosine A2A receptor; CCL-2, C-C motif chemokine ligand 2; CD27, cluster of differentiation 27; CD28, cluster of differentiation 28; CD70, cluster of differentiation 70; CD80, cluster of differentiation 80; CD86, cluster of differentiation 86; CD121, cluster of differentiation 121; CD226, cluster of differentiation 226; CTLA-4, cytotoxic T lymphocyte-associated protein 4; CXCL9, C-X-C motif chemokine ligand 9; CXCL10, C-X-C motif chemokine ligand 10; CXCL11, C-X-C motif chemokine ligand 11; CXCL12, C-X-C motif chemokine ligand 12; DCs, dendritic cells; FASL, Fas ligand; ICAM-1, intercellular adhesion molecule 1; IDO, indoleamine 2,3-dioxygenase; IL-6, interleukin-6; IL-10, interleukin-10; IL-35, interleukin-35; LAG-3, lymphocyte activation gene 3; LKB1, liver kinase B1; MDSCs, myeloid-derived suppressor cells; NFAT, nuclear factor of activated T cells; NKG2D, natural killer group 2 member D; OX-40, tumor necrosis factor receptor superfamily member 4; OX-40L, OX-40 ligand; PD-1, programmed cell death protein 1; PD-L1, programmed death-ligand 1; PGE2, prostaglandin E2; PTEN, phosphatase and tensin homolog; TAMs, tumor-associated macrophages; TAP, transporter associated with antigen processing; TIGIT, T cell immunoreceptor with immunoglobulin and immunoreceptor tyrosine-based inhibitory motif domains; TIM-3, T cell immunoglobulin and mucin-domain containing-3; TP-53, tumor protein p53; VCAM-1, vascular cell adhesion molecule 1; VEGF, vascular endothelial growth factor; VISTA, V-domain immunoglobulin suppressor of T cell activation. The figure was created using BioRender.

The cancer–immunity cycle often begins with tumor antigen release and dendritic cell (DC)-mediated antigen processing, followed by T cell priming and trafficking through the circulation, and finally infiltrating into the TME. This is followed by tumor-cell recognition and cytotoxic killing by T cells with subsequent antigen release [47]. At each stage, the antitumor immune response is shaped by stimulatory and inhibitory molecular signals [47,48]. Costimulatory pathways such as CD80/86-CD28 and tumor necrosis factor receptor superfamily member 4–tumor necrosis factor ligand superfamily member 4 (OX40–OX40L) pathway promote T cell activation [49,50], whereas immune checkpoints, including CTLA-4, restrain the effector function [50,51] (Fig. 2).

In addition to these immune checkpoints, the TME establishes physical and biochemical barriers that limit effective immune responses. Dense and highly crosslinked ECM networks, mainly produced by cancer-associated fibroblasts (CAFs), restrict immune cell infiltration and create “immune-excluded” regions [52]. Collagen [53], FN [54], hyaluronan [55], and PGs [56] increase ECM stiffness, creating “immune-excluded” zones where cytotoxic T cells and natural killer (NK) cells cannot reach tumor cells. The abnormal vasculature in tumors, often irregular, leaky, and poorly perfused, contributes to the poor trafficking of immune cells [57]. These result in heterogeneous blood flow and nutrient deprivation within the tumor, further compromising the immune system’s ability to target and eliminate cancer cells [58]. Additionally, hypoxic conditions within the tumor stabilize hypoxia-inducible factor-1α (HIF-1α), which promotes the expression of immunosuppressive molecules such as PD-L1 and recruits immunosuppressive cells like T regulatory cells (Tregs) and myeloid-derived suppressor cells (MDSCs). Hypoxia-driven up-regulation of VEGF further inhibits immune cell function by promoting aberrant blood vessel formation, exacerbating the TME’s immunosuppressive nature [7]. Hypoxia also up-regulates immunosuppressive factors such as VEGF, further inhibiting immune cell function [59].

The ECM components, such as GAGs in the TME, also play a role in sequestering chemokines, shaping gradients that trap immune cells in the stroma rather than directing them to the tumor core [60]. These ECM-mediated chemokine gradients further hinder effective immune cell recruitment and activation within the tumor. Such physical, metabolic, and signaling barriers collectively facilitate immune evasion, enabling tumor cells to survive, proliferate, invade tissues, and resist immunotherapy. This phenomenon, referred to as cancer immunoediting, describes the process by which immune pressure selects for tumor clones that are more invasive, stem-like, and less immunogenic [61].

Furthermore, specific biochemical and immunosuppressive signaling pathways facilitate the recruitment of immunosuppressive cells, Tregs, and MDSCs into the TME, thereby inhibiting cytotoxic T cells [62]. One well-known mechanistic example is the signal transducer and activator of transcription 3 (STAT3)/C–C motif chemokine ligand 2 (CCL2)/C–C motif chemokine receptor 2 (CCR2) signaling axis [63], where constitutive STAT3 activation in tumor cells induces the secretion of CCL2 that recruits CCR2^+^ myeloid cells to the TME. These recruited cells differentiate into MDSCs and regulatory T cells, which release immunosuppressive mediators including interleukin-10 (IL-10), TGF-β, and arginase-1, ultimately suppressing CD8^+^ cytotoxic T cell activity and promoting tumor immune escape [63,64]. Specific cytokines and key soluble factors, such as IL-10, TGF-β, and others, create a localized immunosuppressive milieu that further dampens immune responses [65]. Anti-apoptotic proteins and immune resistance factors, including B cell lymphoma 2 (BCL-2) [66], cellular FLICE (Fas-associated protein with death domain-like IL-1β converting enzyme)-inhibitory protein (c-FLIP) [67], Serpin B9 [68], and proteinase inhibitor-9 [69], contribute to tumor-cell survival by protecting tumor cells from immune-mediated killing.

Despite these challenges, recent advances in cancer immunotherapy have demonstrated remarkable promise. T cell-based therapies, such as chimeric antigen receptor (CAR)-T cell therapy, have shown significant efficacy in treating hematologic cancers, and monoclonal antibodies targeting immune checkpoints, such as CTLA-4 [70] and PD-1 [71], have improved survival in patients with melanoma and other cancers. These immunotherapies work by blocking inhibitory signals that tumors use to escape immune detection, thereby enhancing the body’s immune response against cancer cells [72].

Tumors with specific immune evasion mechanisms, such as the expression of immune checkpoints or the presence of dense ECM barriers, may remain resistant to treatment [73]. As a result, the ongoing challenge in cancer immunotherapy is to develop strategies that overcome these obstacles, improve patient selection, and enhance the therapeutic response across various cancer types. TME interactions between immune cells and ECM can result in ECM stiffness during the tumorigenesis process, impair normal mechanotransduction, and accelerate the development of malignancy. Therefore, a comprehensive understanding of the dysregulation of the ECM in the TME will be highly beneficial in understanding the immune escape [58,59].

This review provides a deeper understanding of the ECM and its components, emphasizing their critical role in tumor immune evasion. It delves into the signal transduction mechanisms by which tumor cells evade host immune surveillance, offering a comprehensive analysis of how ECM alterations influence these processes. It also focused on the disruption of normal mechanotransduction pathways within the ECM and how such disruptions contributed to tumor progression. The review also highlighted the substantial impact of ECM components on tumor immunity, particularly through their modulation of immune checkpoints and signaling pathways that enabled immune evasion. Furthermore, it discussed ECM-targeted therapeutic strategies and potential approaches for improving immunotherapy by modulating ECM remodeling and ECM-mediated mechanisms of tumor immune evasion. By highlighting key signaling pathways and ECM-related markers, the review suggested that targeting these components could enhance the efficacy of immunotherapies. It also emphasized the role of innovative technologies, such as single-cell RNA sequencing (scRNA-seq) and proteomics, in advancing our understanding of ECM dynamics in the TME. These cutting-edge approaches were poised to offer novel strategies for overcoming immune escape mechanisms, providing promising avenues for future cancer therapies.

## Basic Principles of ECM and Immune Escape

### ECM in normal physiology and pathological ECM remodeling

ECM is essential across multiple biological contexts, including early developmental stages, postnatal tissue maturation, wound healing, and the preservation of tissue and organ homeostasis [30,74]. A defining feature of normal ECM biology is its continuous turnover, which reflects a tightly regulated balance among matrix synthesis, posttranslational modification, structural maturation, crosslinking, and enzymatic degradation [75]. Stromal cells such as fibroblasts and epithelial cells synthesize and secrete ECM proteins to sustain matrix renewal, after which these components undergo key posttranslational modifications, including hydroxylation, glycosylation, and sulfation, that are necessary for correct fibril assembly and biological activity [76]. Enzymatic crosslinking, mainly mediated by lysyl oxidases and transglutaminases, reinforces tensile strength and mechanical integrity, whereas matrix degradation is driven by remodeling enzymes such as MMPs, A disintegrin and metalloproteinase with thrombospondin motifs (ADAMTs), and cathepsins, which cleave ECM components to enable tissue remodeling and cell migration. Importantly, degradation products can also act as bioactive matrikines that modulate immune and inflammatory signaling [77]. Under physiological conditions, these processes are precisely coordinated to preserve ECM architecture, mechanics, and tissue equilibrium. In contrast, dysregulated ECM turnover in cancer leads to excessive matrix deposition, aberrant crosslinking, and uncontrolled proteolysis, resulting in increased stiffness, altered architecture, and the formation of immunosuppressive niches that promote tumor progression and immune evasion [78].

#### Physiological functions and tissue-specific composition of the ECM

In normal tissues, the ECM serves not only as a structural scaffold but also as a dynamic regulator of cell–cell communication, proliferation, differentiation, and tissue homeostasis [79]. Its composition varies considerably across tissue types according to organ-specific functional demands. For example, loose connective tissues, such as blood vessels, contain reticular fibers and associated proteins, whereas bone ECM is enriched in thick collagen fibers and a mineralized matrix [4]. In circulating blood components, including platelets and red blood cells (RBCs), the extracellular environment is dominated by plasma and plasma proteins [80]. At the same time, the ECM of circulating blood cells, such as platelets and RBCs, is rich in plasma and plasma proteins, which are its major components [81]. Functionally, collagen and elastin-rich fibers support tissue integrity and elasticity, while glycoproteins, PGs, and hyaluronan contribute to hydration, signal transduction, mechanosensing, and biochemical cue presentation. The physical stress on tissues is primarily controlled by polysaccharide gels (hyaluronan) and fibrillar proteins [82]. Thus, a sequentially regulated and strictly coordinated ECM is the major regulator of inter/intracellular signaling and mechanosensing, and serves as the anchorage site for all standard biochemical cues and physiological processes [83]. Collectively, these components allow the ECM to act as a critical platform for both structural support and intercellular signaling.

#### Dysregulated ECM remodeling in cancer

**Fig. 3.**
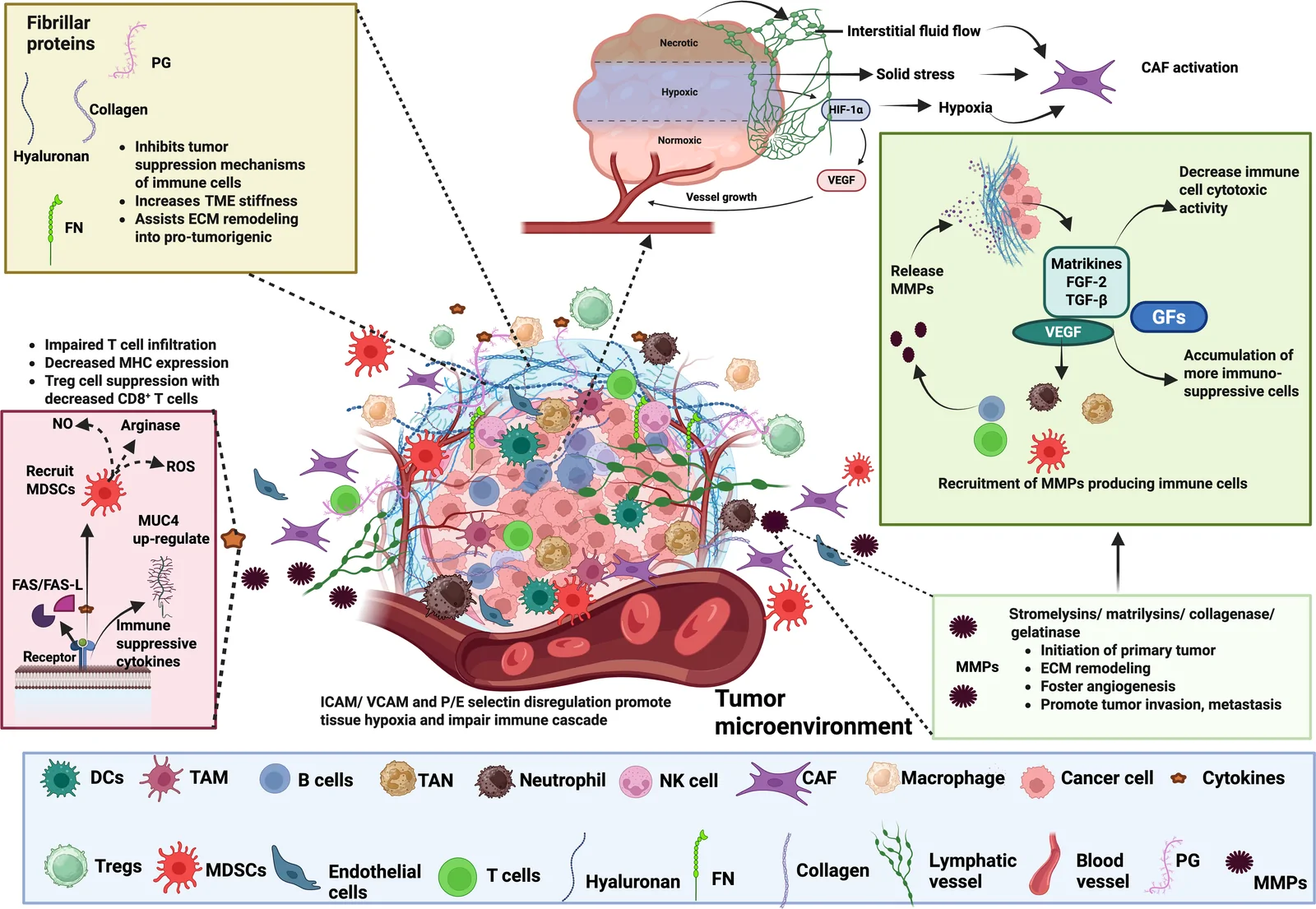
ECM remodeling, hypoxia, vascular dysfunction, and protease-driven immune suppression in the tumor microenvironment. The figure illustrates how ECM remodeling cooperates with hypoxia, abnormal vascular and lymphatic flow, MMP activity, and suppressive immune-cell recruitment to establish an immunosuppressive TME. The central panel depicts a heterogeneous tumor niche composed of cancer cells, CAFs, endothelial cells, DCs, T cells, NK cells, B cells, Tregs, TAMs, macrophages, TANs, neutrophils, MDSCs, cytokines, blood vessels, lymphatic vessels, and ECM components, including collagen, FN, hyaluronan, PGs, and MMPs The upper-left inset summarizes the contribution of fibrillar and nonfibrillar ECM components, including collagen, hyaluronan, FN, and PGs. These matrix components increase TME stiffness, assist ECM remodeling into a protumorigenic architecture, and impair immune-mediated tumor suppression. A dense and remodeled ECM limits T cell infiltration, reduces MHC expression, promotes Treg-mediated suppression, and decreases the abundance and activity of CD8^+^ T cells. The lower-left inset shows how tumor and stromal signals recruit MDSCs and amplify immunosuppressive mediators, including NO, arginase, ROS, MUC4 up-regulation, Fas/FasL signaling, and immunosuppressive cytokines, thereby weakening antitumor immune responses. The upper-middle inset highlights spatial gradients of normoxia, hypoxia, and necrosis within the tumor. Abnormal interstitial fluid flow and solid stress contribute to vessel compression, hypoxia, and CAF activation. Hypoxia stabilizes HIF-1α, which induces VEGF and promotes aberrant vessel growth. These vascular abnormalities, together with dysregulated endothelial adhesion molecules, including ICAM, VCAM, and P-/E Selectins, promote tissue hypoxia and impair immune-cell trafficking and immune-cascade activation. The right inset depicts MMP-mediated ECM degradation and matrikine generation. MMPs, including stromelysins, matrilysins, collagenases, and gelatinases, participate in primary tumor initiation, ECM remodeling, angiogenesis, invasion, and metastasis. MMPs release generates bioactive matrikines and growth factors, including FGF-2, TGF-β, VEGF, and other GFs. These mediators recruit additional MMP-producing immune cells, reduce immune-cell cytotoxic activity, and favor the accumulation of immunosuppressive cells. Collectively, the figure emphasizes that ECM remodeling is not only a structural feature of tumors but also an active regulator of immune escape. Through increased matrix stiffness, altered interstitial flow, hypoxia, CAF activation, MMP-driven proteolysis, impaired antigen presentation, reduced T cell infiltration, and suppressive myeloid-cell recruitment, the remodeled ECM creates a self-reinforcing protumorigenic niche that supports immune evasion, angiogenesis, tumor invasion, and metastatic progression. B cells, B lymphocytes; CAF, cancer-associated fibroblast; CD8, cluster of differentiation 8; DCs, dendritic cells; ECM, extracellular matrix; FAS/FAS-L, Fas/Fas ligand; FGF-2, fibroblast growth factor 2; FN, fibronectin; GFs, growth factors; HIF-1α, hypoxia-inducible factor 1α; ICAM, intercellular adhesion molecule; MDSCs, myeloid-derived suppressor cells; MHC, major histocompatibility complex; MMPs, matrix metalloproteinases; MUC4, mucin 4; NK cell, natural killer cell; NO, nitric oxide; P/E-Selectin, platelet/endothelial selectin; PG, proteoglycan; ROS, reactive oxygen species; TAM, tumor-associated macrophage; TAN, tumor-associated neutrophil; T cell, T lymphocyte; TGF-β, transforming growth factor-β; TME, tumor microenvironment; Tregs, regulatory T cells; VCAM, vascular cell adhesion molecule; VEGF, vascular endothelial growth factor. The figure was created using BioRender.

Alterations in ECM structure and composition profoundly affect immune cell behavior and tissue function. In pathological conditions, ECM remodeling becomes dysregulated, shifting the balance toward excessive synthesis, abnormal crosslinking, or impaired degradation. In cancer, fibrosis, and chronic inflammation, the ECM often becomes dense, stiff, and aberrantly crosslinked, altering its biochemical and biomechanical properties [84]. This subsequently impacts various immune responses, either by creating physical barriers or by regulating signaling pathways, ultimately leading to pathological outcomes such as cancer or fibrosis [85]. ECM remodeling is not only structural but also regulates immune infiltration, hypoxia, angiogenesis, cytokine signaling, CAF activation, matrix metalloproteinase (MMP) activity, and accumulation of immunosuppressive cells. Thus, a pathologically remodeled ECM facilitates tumor-cell proliferation, invasion, and metastasis by providing mechanical cues and activating signaling pathways [86]. This further evokes an immune-excluded niche that physically blocks cytotoxic immune cells and suppresses effective antitumor immunity [87]. In parallel, remodeled ECM supports angiogenesis, disrupts normal tissue architecture, and contributes to hypoxia, thereby further reinforcing a tumor-promoting and immunosuppressive microenvironment [88] (Fig. 3).

#### Cellular and enzymatic drivers of ECM remodeling

ECM homeostasis is regulated by a broad network of secreted enzymes and inhibitors that collectively determine its structural, mechanical, and signaling properties [89]. Key mediators include cross-linking enzymes [e.g., lysyl oxidase (LOX) and transglutaminases] [90], proteases (e.g., MMPs, heparanase, and cathepsins) [91], modifying enzymes (e.g., extracellular kinases and sulfatases) [92], and protease inhibitors (e.g., tissue inhibitors of metalloproteinases, cystatins, and serpins) [93]; each can alter the structural, mechanical, and biochemical properties of the ECM. Remodeling of the ECM by serine proteases, kallikrein-related peptidases (KLKs) [94], urokinase-type plasminogen activator (uPA) [95], furin [96], high temperature requirement A (HtrA) [97], granzymes [98], matriptase [99], and hepsin [100], has been reported as a prerequisite for tumor progression. Importantly, ECM remodeling is not driven solely by tumor cells. CAFs and immune cells actively participate in matrix deposition and reorganization, and sustained crosstalk among tumor cells, fibroblasts, and myeloid populations generates a self-reinforcing network that drives persistent ECM remodeling within the TME [101].

#### BM as a barrier to immune surveillance

The BM is a specialized, densely organized ECM structure that regulates tissue compartmentalization and immune cell trafficking [102]. In tumors, abnormal thickening, crosslinking, or disruption of the BM, often driven by altered laminin and collagen IV deposition, creates both physical and biochemical barriers to cytotoxic lymphocyte infiltration [77]. Concurrently, BM components engage inhibitory receptors on immune cells and modulate chemokine availability, impairing immune surveillance and facilitating tumor immune evasion [103].

#### Interstitial matrix in immune exclusion and tumor progression

The interstitial matrix, composed predominantly of fibrillar collagens, FN, PGs, hyaluronan, GAGs, and other glycoproteins, plays a central role in shaping antitumor immune responses [104]. The ground substance is a gel-like matrix composed of PGs, GAGs, and glycoproteins that fills the space between the fibers and cells [105]. In tumors, excessive matrix deposition, collagen fiber alignment, and enzymatic crosslinking increase tissue density and stiffness, forming a physical barrier that restricts immune cell infiltration and motility [105]. At the same time, the interstitial matrix acts as a reservoir for signaling molecules, including chemokines and GFs, and their sequestration can distort chemotactic gradients and impair effective immune cell positioning [106]. Additionally, the interstitial matrix sequesters chemokines and GFs, distorting immune cell gradients and impairing effective immune surveillance [107]. Together with BM alterations, these structural and biochemical abnormalities promote immune exclusion and support tumor immune evasion.

### TME crosstalk remodels the ECM to support immune evasion and tumor growth

The constant cell–cell communication of the TME actively reshapes ECM structure, composition, and mechanics, thereby creating conditions that favor tumor-cell survival and proliferation [87]. Within the TME, coordinated interactions among malignant cells, endothelial cells, CAFs, and immune cells continuously reshape the ECM through paracrine cues, mechanical signaling, and metabolic exchange [108]. Tumor-derived factors, such as TGF-β, platelet-derived growth factor (PDGF), and IL-6, activate CAFs and induce a contractile phenotype via the RhoA/ROCK (Rho-associated protein kinase)-dependent actomyosin pathway [109]. This contractile force is transmitted to the surrounding matrix through integrin-based adhesions, promoting ECM contraction and compaction [110]. In parallel, CAF-driven collagen fiber alignment and LOX-mediated collagen crosslinking enhance matrix stiffness and tensile strength, thereby establishing a progressively rigid tumor niche [111].

**Fig. 4.**
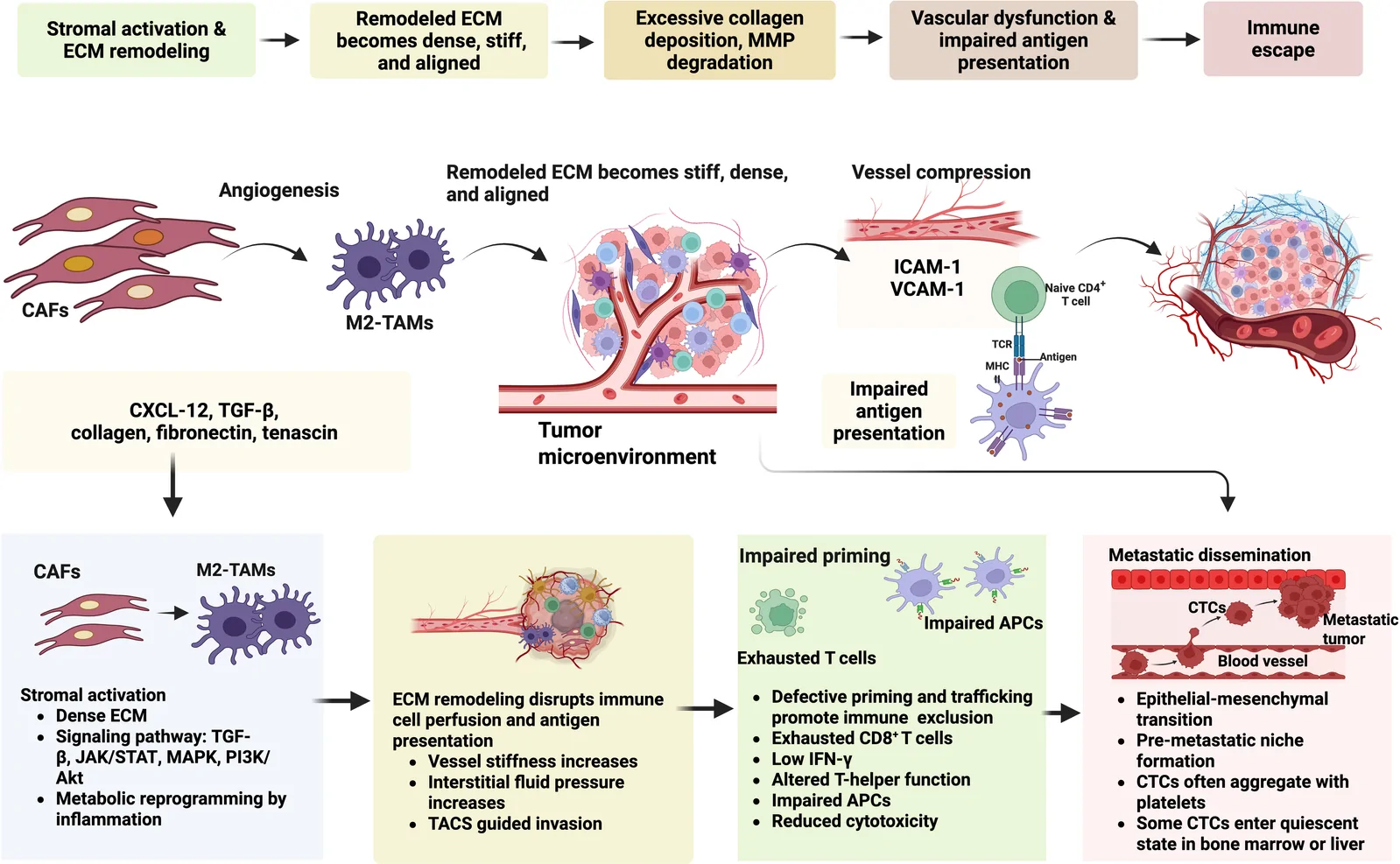
Stromal activation and ECM remodeling drive vascular dysfunction, impaired antigen presentation, immune escape, and metastatic dissemination. The figure illustrates a stepwise model in which stromal activation promotes extracellular matrix (ECM) remodeling, generating a dense, stiff, and aligned matrix that disrupts immune surveillance and facilitates tumor progression. Activated CAFs and M2-TAMs initiate stromal remodeling through angiogenic and inflammatory programs and by producing ECM-associated mediators, including CXCL-12, TGF-β, collagen, fibronectin, and TN. These factors promote excessive collagen deposition, matrix crosslinking, and proteolytic remodeling, including MMP-mediated ECM degradation. As the ECM becomes increasingly dense, stiff, and aligned, the remodeled TME mechanically compresses blood vessels and alters vascular function. This contributes to vessel compression, altered endothelial adhesion molecule expression, including ICAM-1 and VCAM-1, impaired immune-cell perfusion, and defective trafficking of antigen-presenting cells and effector lymphocytes. The remodeled ECM also disrupts antigen presentation by impairing the interaction between MHC-presented antigen and the TCR on naïve CD4^+^ T cells, thereby weakening T cell priming and adaptive immune activation. The lower panels summarize the cellular and functional consequences of ECM remodeling. CAFs and M2-TAMs reinforce stromal activation by promoting dense ECM formation, inflammatory metabolic reprogramming, and activation of signaling pathways such as TGF-β, JAK/STAT, MAPK, PI3K/AKT, and other profibrotic or inflammatory axes. ECM remodeling subsequently disrupts immune-cell perfusion and antigen presentation by increasing vessel stiffness, expanding interstitial fluid pressure, and enabling TACS-guided invasion. These changes support the development of an immunosuppressive niche characterized by defective immune priming and trafficking, exhausted CD8^+^ T cells, reduced IFN-γ production, altered T helper cell function, impaired APCs, and reduced cytotoxicity. Together, these stromal and immune alterations promote immune escape and facilitate metastatic dissemination. The remodeled ECM supports EMT premetastatic niche formation, intravasation and circulation of CTCs, aggregation of CTCs with platelets, and the entry of some CTCs into quiescent states in distant organs such as the bone marrow or liver. Overall, the figure highlights how CAF- and TAM-driven ECM remodeling links stromal activation to vascular dysfunction, impaired antigen presentation, immune suppression, and metastasis. APCs, antigen-presenting cells; CAFs, cancer-associated fibroblasts; CD4^+^, cluster of differentiation 4; CD8^+^, cluster of differentiation 8; CTCs, circulating tumor cells; CXCL-12, C-X-C motif chemokine ligand 12; ECM, extracellular matrix; ICAM-1, intercellular adhesion molecule 1; IFN-γ, interferon-γ; JAK/STAT, Janus kinase/signal transducer and activator of transcription; MAPK, mitogen-activated protein kinase; M2-TAMs, M2-like tumor-associated macrophages; MHC, major histocompatibility complex; MMP, matrix metalloproteinase; PI3K/AKT, phosphoinositide 3-kinase/protein kinase B; TACS, tumor-associated collagen signature; TCR, T cell receptor; TGF-β, transforming growth factor-β; TME, tumor microenvironment; TN, tenascin; VCAM-1, vascular cell adhesion molecule 1. The figure was created using BioRender.

Stromal activation is a central driver of ECM-mediated immune escape. Activated CAFs and M2-polarized tumor-associated macrophages (M2-TAMs) promote the deposition and remodeling of collagen, FN, and other matrix components through signaling pathways such as TGF-β, Janus kinase/signal transducer and activator of transcription (JAK/STAT), mitogen-activated protein kinase (MAPK), and phosphoinositide 3-kinase/protein kinase B (PI3K/AKT) [47]. As the ECM becomes increasingly dense, stiff, and aligned, it compresses blood vessels, elevates interstitial fluid pressure, disrupts immune cell perfusion, and restricts antigen presentation. These changes further impair T cell priming, trafficking, and cytotoxic activity, thereby promoting immune exclusion. In parallel, remodeled ECM architecture facilitates invasion, circulating tumor-cell dissemination, premetastatic niche formation, and metastatic progression (Fig. 4).

In parallel, localized ECM degradation by MMPs and cathepsins releases matrix-bound GFs, further reshaping the biochemical landscape and leading to local ECM softening, with the release of specific GFs, such as TGF-β, VEGF, and FGF [99]. This proteolytic remodeling induces localized ECM softening and establishes biochemical gradients, thereby creating spatial heterogeneity within the predominantly stiff tumor matrix. As a result, the remodeled ECM functions as a GF reservoir that continuously sustains proliferative signaling. These mechanical and biochemical changes directly enhance tumor-cell proliferation by reinforcing integrin-dependent mechanotransduction pathways, including focal adhesion kinase/Src (FAK/Src) [112], MAPK/extracellular signal regulatory kinase (ERK) [113], and Yes-associated protein/transcriptional coactivator with PDZ-binding motif (YAP/TAZ) signaling [114], which drive cell-cycle progression, suppress apoptosis, and promote long-term survival [99]. In addition, ECM remodeling facilitates metabolic coupling between CAFs and tumor cells, thereby further supporting tumor-cell proliferation. Similarly, juxtaposed stiff and compliant ECM niches generate mechanical heterogeneity that differentially regulates tumor-cell behavior; stiff regions activate integrin-FAK-YAP/TAZ signaling [115]. In contrast, softer areas permit proliferation and metastatic competence. Loss of contact inhibition within this heterogeneous landscape promotes clonal expansion, with maximal proliferation occurring at the stiffness transition zones [116]. Beyond tumor growth, cell–cell communication-driven ECM remodeling plays a central role in immune escape. CAF and tumor-derived signals induce ECM contraction, collagen crosslinking, and fiber alignment, leading to matrix densification and reduced pore size [117]. Dense and stiff ECM physically restricts cytotoxic T cell infiltration, impairs immune synapse formation through altered mechanotransduction, and promotes the accumulation of immunosuppressive populations such as regulatory T cells and M2-polarized macrophages [104]. Moreover, ECM-induced hypoxia and metabolic reprogramming further suppress antitumor immunity by enhancing immune-checkpoint signaling. Collectively, cell–cell communication does not merely accompany ECM remodeling but actively converts the ECM into a mechanically stiff, biochemically enriched, and immunosuppressive signaling platform, thereby coupling tumor proliferation with immune evasion within the TME.

### ECM remodeling as an active immune modulator of tumor progression

During tumor progression, malignant cells employ diverse strategies to evade immune surveillance and suppress antitumor immunity [118]. Such adaptations facilitate immune escape, reprogram host immunity, and promote the establishment of a permissive microenvironment that sustains tumor growth. Concurrently, coordinated interactions among multiple cellular components within the TME drive ECM remodeling [119].

Tumor cells directly contribute through aberrant collagen deposition, FN assembly, and secretion of matrix-degrading enzymes such as MMPs [104]. CAFs act as principal architects of the remodeled ECM, responding to paracrine cues including TGF-β, IL-6, and PDGF by enhancing matrix deposition, contractility, and LOX-mediated collagen crosslinking [120]. TAMs further reshape the ECM through protease and cytokine release, while endothelial cells contribute by altering the BM and promoting vascular compression, which further increases matrix stiffness [56]. Collectively, these biochemical and mechanical cues sustain ECM remodeling and establish a tumor-permissive, immunomodulatory microenvironment [89]. Effective antitumor immunity requires coordinated antigen capture, presentation, T cell priming, and tumor-cell killing. Within the TME, APCs, particularly DCs, capture tumor-derived antigens and migrate to lymph nodes, where they present them to naive T cells via major histocompatibility complex (MHC) class I and II molecules [89]. With appropriate costimulatory signals, this promotes the generation of antigen-specific effector T cells, which then recognize and eliminate tumor cells through T cell receptor–MHC interactions and release of cytotoxic mediators such as perforin and granzymes [89]. Disruption of any step in this cascade weakens antitumor immunity and facilitates immune escape.

Cancer cells further evade immune surveillance by engaging immune-checkpoint pathways and suppressing T cell and DC activity. These mechanisms impair immune cell recruitment and effector function while promoting an immunosuppressive TME [89]. In parallel, extensive ECM remodeling, anoikis resistance, escape from tissue size control, and sustained GF signaling reinforce tumor progression [47]. Stromal and immune components, including CAFs, TAMs, and other myeloid populations, amplify this state through cytokine release, hypoxia-associated signaling, and reactive oxygen species (ROS), thereby promoting epithelial–mesenchymal transition (EMT), invasion, and metastasis. Desmoplastic remodeling also enhances mechanotransductive signaling by strengthening cell–matrix and cell–cell interactions [121]. This enables tumor cells to tolerate elevated matrix stress while maintaining proliferation. Such adaptation is linked to aberrant activation of Hippo pathway-associated regulators, including Yes proto-oncogene 1 (YES1), YAP, and WW domain-containing transcription regulator protein 1 (WWTR1)/TAZ, downstream of integrin-mediated mechanotransduction [116].

## General Tumor Immune Evasion Mechanisms

### Tumor heterogeneity

Within the tumor bulk, individual cell populations exhibit pronounced heterogeneity in gene expression profiles, cellular morphology, metabolic states, biochemical activities, and proliferative capacity. This intratumoral diversity underlies differential sensitivity to therapeutic interventions, resulting in variable treatment responses across distinct tumor-cell subsets [122]. This will disrupt antitumor immunity and immune evasion by T cells. Specific immunogenic subclones are frequently eliminated through T cell-mediated immunoediting. However, cancer cells that evade this process ultimately give rise to distinct immune-escaped tumor populations. This is referred to as intratumor heterogeneity. For instance, CD19 is an established and effective target for CAR-T therapy in B cell acute lymphoblastic leukemia (B-ALL) [123]; however, its heterogeneous or lost-CD19 expression can contribute to immune escape and the emergence of immune-escaped tumor populations [124,125].

### Low tumor mutation burden

Tumor mutation burden (TMB) reflects the number of somatic mutations in cancer cells, expressed as mutations per megabase (mut/Mb). Tumors with low mutational burden, often defined as fewer than 10 mut/Mb, tend to produce fewer neoantigens. As a result, these tumors are less likely to be recognized and presented by MHC molecules, thereby impairing T cell activation and immune recognition [126]. The association between TMB and endogenous processes, such as deficient mismatch repair (dMMR) and microsatellite instability-high (MSI-H), is the focus of several ongoing immunotherapy studies [127,128]. If a tumor possesses more somatic mutations, it is more likely to form immunogenic neoantigens [128]. Therefore, TMB has emerged as a potential predictive biomarker for identifying patients likely to respond to immunotherapy [128,129]. Large-scale pan-cancer studies have documented that among tumor types, melanoma is reported with the highest levels of TMB [130], followed by relatively high TMB in non-small cell lung cancer (NSCLC) [130]. In contrast, lower TMB levels have been reported in leukemias [131].

### Down-regulation of antigen processing, presentation, and antigen loss

Down-regulation of antigen processing and presentation will lead to an immunosuppressive environment. This will affect the binding of MHC class molecules and further restrict T cell antigen recognition. This MHC-I antigen presentation is how CD8^+^ cytotoxic T cells find and eradicate tumor cells. Tumor cells generally express a dysregulated pattern of MHC-I, β2-microglobulin (B2M), or other proteasome components required for antigen presentation to cytotoxic T cells. DCs are the most efficient APCs. Any DC maturation dysfunction caused by traps in DC maturation will abrogate their roles [132]. During the immunoediting process, tumors acquire resistance to immunotherapy when most immunostimulatory antigens are eliminated by immunoselecting cells with low immunogenicity. The immune system becomes oblivious to cancer cells when neoantigens are lost, which leads to immune evasion and resistance to immune-checkpoint therapy [133,134]. Defects in the antigen-presentation machinery enable tumor cells to escape cytotoxic T cell recognition. Loss or dysfunction of B2M, an essential component of MHC-I molecules, is a well-established mechanism of resistance to immunotherapy [135,136]. In mismatch repair-deficient cancers, B2M mutations can abolish surface expression of human leukocyte antigen (HLA) class I molecules, thereby preventing effective T cell-mediated immune surveillance and facilitating tumor immune evasion [137,138].

### Immune-checkpoint activation

Immune checkpoints are activated when protein receptors/ligands on the T cell surface interact with their corresponding binding partners on tumor cells or APCs, such as DCs. Upon interaction, these immune-checkpoint proteins transmit either activating or inhibitory signals to T cells. Most tumor immunotherapy drugs work by blocking these immune-checkpoint proteins to prevent further partner protein association and modulate signal transduction pathways [51]. These immune-checkpoint protein targets include CTLA-4 [139], PD-1 [140], adenosine A2A receptor (A2AR) [141], signal regulatory protein α (SIRPα) [142], and the newly discovered T cell immunoreceptor with Ig and immunoreceptor tyrosine-based inhibitory motif domains (TIGIT) [143].

### Lineage switch

Lineage switch (LS) is the phenomenon in which a tumor-cell undergoes a change in cellular identity that leads to conversion from one lineage or differentiation state to another. It represents either a relapse of the initial tumor with phenotypic/genotypic heterogeneity or the emergence of a new clone [144]. LS has been reported as a mechanism of relapse after antigen-targeted immunotherapies and can adversely affect cancer treatment outcomes, especially CAR-T therapy [145,146]. The most complicated part of the post-CAR-T therapy is the immunophenotypic heterogeneity of the relapse, with the rarest and most alarming being the LS. Due to rare occurrences, studies are relatively scarce, with only a few case reports [147]. Recent evidence indicates that approximately 8% of patients with relapsed/refractory B cell acute lymphoblastic leukemia (r/r B-ALL) experienced LS following CD19-targeted CAR-T therapy [148]. In leukemias, this phenotypic shift is frequently associated with the lysine methyltransferase 2A (KMT2A) gene rearrangement, leading to the emergence of genetically distinct leukemic subclones with enhanced aggressiveness and an unfavorable clinical outcome [149]. Recent evidence indicates that B-ALL can evade immune pressure through LS to acute myeloid leukemia (AML), a phenomenon associated with adverse clinical outcomes [150]. These findings suggest that intrinsic lineage plasticity in B-ALL may facilitate myeloid relapse and contribute to poor prognosis.

## ECM-Mediated Immune Evasion Mechanisms

The ECM plays a central role in shaping tumor–immune interactions and is now recognized as a critical determinant of immune escape in cancer [87,151]. Far from being a static structural framework, the ECM undergoes continuous remodeling during tumor progression, driven by reciprocal interactions among malignant cells, stromal cells, infiltrating immune populations, and the ECM structural components [104]. A key feature of ECM-mediated immune evasion is the restriction of immune cell infiltration [104]. Excessive deposition of fibrillar collagens, FN, and other stromal components increases matrix density and tissue stiffness, creating a mechanically hostile environment for cytotoxic T cells and NK cells [56]. This barrier effect is not merely structural; ECM architecture, crosslinking, and fiber alignment can further limit lymphocyte motility, reduce direct tumor–immune cell contact, and promote immune exclusion [152]. Thus, ECM architecture and mechanics directly influence whether immune cells can access, recognize, and eliminate tumor cells. Another major mechanism involves the ability of ECM molecules to regulate the spatial distribution and bioavailability of cytokines, chemokines, and GFs. PGs and GAG-rich structures can bind, sequester, stabilize, or present soluble mediators, thereby shaping local signaling gradients that determine where immune cells migrate and how they function [153]. MMPs are particularly important in this process because they drive ECM turnover while simultaneously regulating immune escape. By degrading BM and interstitial matrix components, MMPs facilitate tumor invasion and alter immune cell access to tumor nests [154]. Cancer immunoediting further contributes to this process by selecting tumor-cell populations that can exploit ECM remodeling to evade immune destruction. Key ECM components, including collagens, PGs, FN, laminins, and MMPs, each contribute in distinct yet interconnected ways to immune exclusion and dysfunction [104]. Collectively, they regulate immune cell infiltration, adhesion, migration, cytokine distribution, and stromal reprogramming, creating a TME that favors tumor persistence and resistance to antitumor immunity. Under immune pressure, tumor cells that survive elimination and equilibrium phases are selected for traits that enhance escape, including the ability to exploit stromal remodeling programs [73]. In turn, the remodeled ECM reinforces this escape phenotype by restricting immune cell access, sustaining suppressive signaling, and protecting residual malignant cells from eradication [30]. Understanding how these ECM components cooperate to establish this immune-resistant niche is essential for designing ECM-targeted strategies that can convert immune-excluded tumors into immunologically responsive ones. The following subsections discuss these major ECM-mediated mechanisms of immune escape and their implications for cancer progression and therapy.

### ECM-driven immune escape through T cell exhaustion, immunosuppression, and impaired infiltration

ECM remodeling is not merely a structural consequence of tumor progression; it actively drives immune escape by altering how tumor antigens are exposed, how immune cells traffic through the tumor, and how inhibitory signals are sustained within the TME. First, ECM-associated barrier formation can contribute to antigen masking [87]. A remodeled pericellular matrix enriched in glycocalyx components, fibrin, FN, and platelet-associated deposits can physically shield tumor-cell surfaces and obscure antigenic epitopes from immune recognition [30]. In parallel, abnormal ECM deposition around tumor nests may limit antigen release and uptake by APCs, thereby weakening efficient priming of antitumor T cell responses [155]. Tumors may further reinforce this defective antigen visibility by altering antigen-processing machinery, including immunoproteasome-associated components such as LIM domain kinase 1 (LIMK1) [156], tapasin [157], calnexin [158], and calreticulin [159]. Second, ECM remodeling promotes impaired T cell infiltration by creating both physical and biochemical barriers. Dense collagen networks, excessive fibrin accumulation, and increased matrix crosslinking stiffen the stroma and compress the interstitial space, which restricts T cell migration into tumor nests [104]. These structural changes also disturb chemokine distribution and generate tortuous migratory paths, causing lymphocytes to remain trapped in stromal or perivascular regions rather than reaching tumor cells [160]. In addition, ECM-driven vascular abnormalities and endothelial dysfunction further reduce effective immune cell extravasation, thereby reinforcing the immune-excluded phenotype [161]. Third, the remodeled ECM supports a profoundly immunosuppressive microenvironment. Matrix stiffening, abnormal integrin signaling, and fibrosis contribute to hypoxia, acidosis, oxidative stress, and nutrient deprivation, all of which suppress T cell activation and favor the recruitment or expansion of immunoregulatory populations such as Treg cells, MDSCs, and TAMs [87]. ECM components and their degradation products can also modulate cytokine and chemokine retention, amplifying inhibitory signaling cascades and sustaining suppressive cell–cell communication within the TME [104]. Thus, ECM remodeling does not simply coexist with immunosuppression; it also helps to establish the biochemical and mechanical conditions that maintain it [104]. Further, this exhausted phenotype is marked by reduced IL-2 production early, followed by loss of tumor necrosis factor-α (TNF-α) secretion, and ultimately diminished IFN-γ production and cytolytic activity accompanied by high levels of PD-1, lymphocyte activation gene 3 protein (LAG-3) [162], CTLA-4 [163], B and T lymphocyte attenuator (BTLA) [164], T cell immunoglobulin and mucin domain-3 (TIM-3) [165], and TIGIT [166], which makes them unable to eliminate cancer cells.

A dysfunctional TME is hostile to immune surveillance failure and immunosuppression in cancer. Therefore, the effectiveness of anticancer immunotherapy primarily depends on this [167]. On the other hand, it leads indirectly to nutrient deprivation (glucose and amino acids), the production of immunosuppressive metabolites, altered lipid metabolism, hypoxia, ROS, and an acidic extracellular pH [168]. Moreover, an immunosuppressed TME is a niche for microbial dysbiosis, microbial activation of inhibitory checkpoints, and the production of metabolites [167].

### Cancer immunoediting: Impact on ECM remodeling and immune escape

Dense, highly crosslinked, and stiffened ECM networks impose physical constraints that limit immune cell infiltration and motility, giving rise to immune-excluded tumor regions where cytotoxic T lymphocytes and NK cells fail to establish effective contact with malignant cells. In parallel, ECM constituents, including collagen, FN, hyaluronan, and PGs, sequester chemokines and GFs, reshaping immune gradients and fostering localized immunosuppressive niches that promote regulatory T cell accumulation and MDSC activity. These combined physical and biochemical barriers suppress cytotoxic immune function and facilitate immune evasion, thereby driving cancer immunoediting, a dynamic process in which sustained immune pressure selectively favors tumor-cell variants with reduced immunogenicity and enhanced malignant potential [73]. There are 3 stages to this cancer immunoediting process: elimination, equilibrium, and escape. The first phase involves immunosurveillance, while the second involves selecting tumor variants. This ultimately leads to the third stage of immunological escape, a hallmark of cancer, during which tumor cells grow unchecked, and the tumor manifests clinically. These phases represent a dynamic interplay between the immune system’s ability to eliminate cancerous cells [169].

A study by Matsushita et al. [170] on cancer exome analysis revealed that cancer immunoediting is the consequence of a T cell-dependent immunoselection process, ultimately leading to the progression of tumor-cell clones that lack potent immunodominant rejection antigens. The stimulator of interferon genes (STING) is an innate immune signaling protein that plays a significant role in all 3 phases of cancer immunoediting, particularly in regulating antitumor immunity [171]. Several studies have demonstrated that activation of STING signaling enhances antitumor immunity [172,173], whereas STING signaling has also been implicated in immune escape, leading to cancer progression in some cases [174–176]. Certain cancers evade immune-mediated elimination by recruiting immunosuppressive cell populations, such as regulatory T cells and MDSCs, thereby establishing a TME that suppresses effective antitumor immune responses [177,178]. During the elimination phase of cancer immunoediting, both adaptive and innate immune mechanisms cooperate to detect and destroy emerging tumor cells [73]. In addition to cytotoxic T lymphocytes, innate immune cells including NK cells, macrophages, and neutrophils play critical roles in recognizing transformed cells and initiating early antitumor responses [179]. However, tumor cells that escape complete eradication may enter the equilibrium phase, in which immune effector mechanisms restrain tumor growth but fail to eliminate all malignant cells [169]. In this state, the residual tumor cells remain in a dormant or clinically undetectable condition for prolonged periods, sometimes lasting years or even throughout the host’s lifespan [180]. This dynamic balance between immune surveillance and tumor-cell persistence represents a key intermediate stage before tumors eventually acquire mechanisms that enable immune escape and disease progression [180].

In addition, adaptive immune components, such as T helper 1 (Th1) cells, cytotoxic T lymphocytes, and cytokines such as IL-2, IL-12, and IFN-γ, are deeply involved in antitumor immunity and cancer immunoediting [181,182]. The immune escape phase in cancer is characterized by the clinical appearance of a tumor that favors the development of rapidly proliferating cells, which are invisible to the immune system. The latent phase involves the acquisition of the ability to invade tissues and form metastases [183]. This phase further promotes the development of necrotic lesions, which trigger inflammation by releasing proangiogenic factors. Several cellular components, such as MDSCs, Treg cells, and various cytokines, including IL-10, contribute to preventing antitumor immunity and facilitating immune escape in the TME. Similarly, specific proteins, such as BCL-2 [184], proteinase inhibitor 9 [185], serpin B9 [68], and cellular FLICE, inhibiting protein like c-FLIP [186] and CASP8 and FADD-like apoptosis regulator (CFLAR) [187], facilitate immune resistance and immune escape in the tumor. Collectively, these findings highlight how ECM remodeling and tumor-driven immunomodulatory mechanisms cooperatively reshape the TME, promoting immune evasion, tumor progression, and resistance to antitumor immune responses.

### Collagens: Impact on immune cell infiltration and activity

Collagens constitute the predominant structural elements of the ECM, accounting for nearly one-third of the total protein content in the human body [188]. To date, 28 distinct collagen types have been identified, and these are structurally and functionally grouped into major subclasses such as fibril-forming collagens, fibril-associated collagens with interrupted triple helices (FACITs), network-forming collagens, transmembrane collagens, endostatin-generating collagens, anchoring fibrils, and beaded filament-forming collagens [189]. Among these, fibrillar collagen type I is the most abundant and represents a principal structural component of the interstitial matrix [12]. Structurally, collagen molecules are composed of 3 left-handed polypeptide α-chains stabilized by interchain hydrogen bonding, with each collagen type defined by a unique α-chain composition [190]. Collagen type I consists of 2 α1 chains and one α2 chain. Accumulating evidence indicates that excessive collagen deposition and enhanced fiber crosslinking are closely associated with increased tumor tissue stiffness [8]. Most cancers, particularly solid tumors, are stiffer than healthy tissue, making them palpably noticeable [191]. Variations in collagen density within the TME modulate immune cell infiltration and shape their interactions with cancer cells. Supporting this concept, Kuczek et al. [192] demonstrated that T cells cultured within dense collagen matrices exhibited markedly reduced proliferative capacity, accompanied by an increased CD4^+^ to CD8^+^ T cell ratio. These findings highlight collagen density as a key regulator of T cell abundance and composition in human breast cancer [192]. Nevertheless, the proliferation of cancer cells was unaffected by collagen density; however, collagen density plays a role in regulating T cell abundance in cancer. It modulates the cytotoxic potential of tumor-infiltrating lymphocytes (TILs) [193]. Alterations in collagen abundance and organization influence the differentiation and effector functions of TILs [192]. TAMs and TILs are major immune components of the TME that critically influence tumor progression and antitumor immunity. Their recruitment, localization, and functional state are strongly shaped by the ECM, particularly collagen, which acts not only as a structural scaffold but also as an active regulator of immune cell behavior. Through these effects, collagen and other ECM components can directly or indirectly alter T cell migration, phenotype, and effector function, thereby promoting immune evasion and tumor progression when aberrantly remodeled [194,195]. These observations have prompted the exploration of collagen-targeting interventions in combination with immune-checkpoint inhibitors (ICIs) to enhance antitumor immunity. One such example is the inhibition of LOX enzyme activity by anti-PD-1 administration, which reverses tumor stiffening and facilitates efficient CD8^+^ T cell infiltration, thereby enhancing PD-1 blockade in murine models [196]. Similarly, in another study, collagen degradation was initiated via IL-17 signaling, which was further augmented by anti-PD-L1-mediated tumor regression in a murine model of cutaneous squamous cell carcinoma (cSCC) [197]. Also, the up-regulation of the mannose receptor C-type 2 (MRC2) collagen receptor genes can reverse the efficacy of anti-PD-1 therapy [198]. Hence, deleting this specific receptor could facilitate CD8^+^ T cell infiltration and enhance the effectiveness of immunotherapy by blocking tumor cells’ immune escape mechanisms [197]. Moreover, the combined effects of TGF-β blockade, PD-L1 blockade, and inhibition of leukocyte-associated immunoglobulin-like receptor 1 (LAIR-1) signaling have been studied in murine mammary carcinoma and colon cancer models [199]. This strategy directly linked collagen remodeling to immune reprogramming, as it altered the tumor collagenous matrix, reduced collagen-mediated immune suppression, enhanced CD8^+^ T cell infiltration and activation, and repolarized suppressive macrophage populations [199]. These findings highlight that targeting collagen-driven tumor remodeling can reshape the immunosuppressive TME and promote durable antitumor immunity. Accordingly, inhibition of FAK reduces fibrotic collagen remodeling and enhances therapeutic responsiveness [200]. Overall, these studies highlight collagen as an active determinant of immune exclusion and therapeutic resistance within the TME, suggesting that interventions targeting collagen deposition, organization, and signaling could potentiate antitumor immunity and improve responses to immune-checkpoint blockade (ICB).

### PGs: Modulation of cytokine gradients and immune cell migration

PGs are emerging as critical regulators of cytokine gradients and immune cell trafficking within the TME, functioning far beyond their traditional role as structural ECM components. Through their GAG chains, PGs bind, immobilize, and present chemokines and cytokines [7], thereby controlling their spatial distribution, local retention, and receptor accessibility to infiltrating immune cells. Under physiological conditions, these interactions orchestrate leukocyte recruitment and positioning; however, in tumors, aberrant PG expression and altered GAG sulfation reprogram these gradients to impede cytotoxic T cell infiltration and favor the accumulation of immunosuppressive cell populations. Importantly, the effects of PGs are highly context dependent [201]. Large chondroitin sulfate-rich proteoglycans (CS-rich PGs), such as versican, can remodel collagen architecture and promote immune-excluded stromal niches, whereas small leucine-rich PGs, such as decorin, can restrain collagen fibrillogenesis and exert tumor-limiting effects [202]. In parallel, HS and chondroitin sulfate (CS) actively regulate chemokine–leukocyte interactions during immune cell recruitment [203], underscoring their role as dynamic organizers of immune signaling rather than passive matrix constituents [204]. Notably, the immunoregulatory influence of PGs is not dictated solely by their abundance; qualitative changes in GAG composition and sulfation can profoundly reshape chemokine sequestration, cytokine presentation, stromal organization, and leukocyte positioning, thereby reinforcing immune escape, tumor progression, and therapeutic resistance [205]. Altogether, by reshaping chemokine presentation, cytokine signaling, and stromal organization, PGs actively contribute to immune escape, tumor progression, and therapeutic resistance.

#### GAG sulfation patterns in chemokine regulation and immune evasion

The functional properties of CS and HS are governed by specific sulfation motifs, such as 4-O/6-O sulfation in CS and variable N/O-sulfation in HS, which selectively control the binding and spatial distribution of chemokines, including CCL2 and chemokine C-X-C motif ligand 9/10/12 (CXCL9/CXCL10/CXCL12) [206]. In turn, these sulfation-dependent interactions determine whether chemokines are retained in the ECM, presented to infiltrating leukocytes, or sequestered away from immune cell receptors, thereby shaping immune cell recruitment [207]. For instance, altered HS sulfation in lung cancer promotes sequestration of CXCL12 within the matrix and disrupts immune cell trafficking [208]. Similarly, accumulation of hyaluronan and sulfated GAGs after anti-VEGF therapy in metastatic colorectal cancer (CRC) contributes to ECM stiffening and the formation of a physical barrier to immunotherapy [209]. Therefore, tumor aggressiveness is influenced less by total PG abundance than by qualitative changes in PG composition and GAG sulfation, which reprogram ECM architecture, chemokine availability, and immune cell positioning [210]. This concept may also explain why high-grade gliomas can remain highly aggressive despite reduced PG levels, as altered GAG chemistry can still favor immune exclusion and protumor signaling [211].

#### PGs linking ECM remodeling and stromal organization to immune escape

Specific PGs, particularly versican and brevican, actively promote tumor progression by linking ECM remodeling to immune exclusion and invasive behavior. In cervical cancer, increased versican expression, together with its associated CS and dermatan sulfate (DS) chains, has been associated with a marked reduction in tumor-infiltrating CD8^+^ T cells, supporting a role in suppressing antitumor immune surveillance [212]. Mechanistically, versican promotes immune evasion by binding and sequestering chemokines through its sulfated CS and DS chains, thereby disrupting the chemokine gradients required for efficient T cell recruitment and favoring the development of an immune-excluded TME [213]. In addition, versican can remodel stromal architecture in ways that further limit cytotoxic lymphocyte infiltration. Similarly, in glioma, brevican enhances tumor aggressiveness by reorganizing the ECM into migration-supportive tracks and activating integrin/CD44-dependent signaling pathways, including FAK/Src, MAPK/ERK, and PI3K/AKT, that promote motility and invasion [214]. At the same time, brevican-rich ECM may restrict immune cell access through both physical and biochemical mechanisms, including matrix compaction and altered chemokine availability [215]. Collectively, these observations indicate that tumor-associated PGs are not only structural ECM components but also active drivers of immune exclusion and malignant progression.

#### Hyaluronan fragments and PG-associated immune signaling

Low-molecular-weight hyaluronan fragments released by tumors promote immune escape by activating DCs through Toll-like receptors (e.g., TLR2 and TLR4), and CD44, which in turn induce CD8^+^ T cell apoptosis via CD3-ε down-regulation and impaired T cell receptor signaling [216–218]. PG–chemokine interactions are central to leukocyte adhesion, trafficking, and recruitment. Chemokines bind receptors on circulating leukocytes and trigger signaling cascades that activate integrins, thereby strengthening leukocyte adhesion to the endothelium [219]. PGs and GAGs regulate immune cell trafficking in the tumor ECM primarily by controlling the spatial presentation and activity of soluble immune mediators. By binding chemokines, cytokines, and GFs, GAG chains immobilize these molecules within tissues, protect them from rapid diffusion, and generate localized gradients that guide leukocyte adhesion, migration, and activation [220]. This function is particularly relevant for chemokines, where GAG-dependent presentation helps establish directional cues for immune cell recruitment. GAGs also interact with several interleukins, including IL-1, IL-2, IL-6, IL-8, IL-12, and IL-15 [221], and with interferons such as IFN-γ, thereby influencing immune cell activation and inflammatory signaling [222]. For example, high-affinity binding of IFN-γ to GAGs can enhance downstream induction of CXCL9, CXCL10, and CXCL11, amplifying leukocyte recruitment [60,223].

Beyond cytokines and chemokines, HS PGs bind VEGF [224], FGFs [225], and TGF-β family members, including TGF-β1 and TGF-β2 [226], thereby shaping cytokine localization, immune cell differentiation, and immunosuppressive signaling within the cancer ECM. GAG structure also determines immune cell trafficking specificity. For instance, differential binding of CCL19 and CCL21 to HS and CS regulates DC migration through lymphatic tissues [227]. In addition, HS and CS can act as damage-associated molecular patterns (DAMPs) that activate TLR4 signaling, linking ECM remodeling to innate immune activation [228]. GAG-mediated regulation further extends to adhesion molecules such as selectins, which support tumor-cell interactions with endothelial cells and platelets during metastasis [229]. For example, P-selectin binding to CS PGs enhances tumor-cell adhesion in metastatic breast cancer models, thereby promoting metastatic dissemination [230,231]. Thus, PGs and GAGs are not passive ECM components but active regulators of immune cell positioning, inflammatory signaling, and metastatic niche formation.

### FN and Laminins: Influence on cell adhesion, migration, and immune surveillance evasion

FN and laminins are major adhesive glycoproteins of the ECM that play central roles in regulating cell adhesion, migration, tissue organization, and signal transduction within the TME [7]. By engaging integrins and other adhesion receptors, they influence cytoskeletal dynamics, cell polarity, survival, and motility, thereby supporting tumor-cell dissemination and stromal remodeling [232]. In cancer, aberrant deposition and organization of FN and laminin-rich matrices not only facilitate invasion and metastatic spread but also reshape the physical and biochemical landscape encountered by immune cells. These changes can impair effective immune cell trafficking, restrict cytotoxic lymphocyte access to tumor nests, and promote immune surveillance evasion [233]. Thus, FN and laminins function not only as structural ECM components but also as dynamic mediators linking adhesion-dependent signaling, migratory behavior, and immune escape.

#### FN and laminins as determinants of cell adhesion, migration, and mechanotransduction

FN is an ECM glycoprotein with multiple functions, but its most prevalent role is to connect integrins on the cell membrane to collagen and GAG-like ECM components [234]. This connection recruits a series of cellular proteins and cytoskeletal structures, which ultimately lead to the formation of focal adhesions (FAs), a typical integrin-based specialized adhesive organelle [235]. This coupling of ECM-FN with actin filaments via FAs eventually drives directed cell migration, with dynamic control of cell adhesion in wound-healing processes and metastatic tumors [236,237]. In particular, integrin α5β1 appears to be a major mediator of FN fibrillogenesis and ECM assembly, while integrin-linked kinase (ILK)-dependent conversion of soluble FN into fibrillar FN enhances cytoskeletal tension and adhesive signaling. These adhesive and mechanical signals are further fine-tuned by posttranslational regulation of integrins. For example, altered N-glycosylation of α5β1 can weaken FN binding and modify migratory behavior. Beyond serving as a structural ligand, FN also participates in broader prometastatic programs by supporting tumor-cell invasion, extravasation, and stromal reprogramming [238].

Earlier studies described context-dependent tumor-suppressive effects of pericellular FN (peri-FN), including reduced migration upon peri-FN hyperdeposition [239,240]. The prevailing evidence indicates that FN predominantly functions as a pro-adhesive, pro-migratory, and pro-metastatic ECM cue across a wide range of tumors, thereby facilitating tumor progression [241–245]. Later evidence redirected the consensus toward its pro-metastatic role in cancer progression [239,240,246–248]. In line with these findings, several studies have compared nontransformed and tumorigenic epithelial cells in nude mouse models and reported reduced cell-surface FN expression along with altered ECM assembly, suggesting an association with the acquisition of malignant characteristics [249,250]. Since integrins are the central FN receptors, integrin-N glycosylation is a key factor contributing to altered FN–integrin interaction. A study showed that N-glycans on α5β1 integrin blocked α5β1-mediated binding to FN and further inhibited subunit association [251]. Distant metastasis of cancer tissues is primarily ascribed to the assembly of peri-FN [252]. The re-expression of peri-FN in transformed rat kidney cells resulted in flattened morphology and surface-associated fibrillar FN, accompanied by normalized monolayer growth, indicating that peri-FN may exert tumor-suppressive effects [249]. However, the role of peri-FN in primary tumor growth remains elusive and inconclusive [253]. Similar to the 2020 study, the group again investigated the impact of ILK on FN fibrillogenesis using human intestinal epithelial cells (HIECs) and further explored the integrin–actin axis signaling [254]. The findings indicate that ILK-dependent conversion of soluble FN into fibrils drives ECM assembly, which is accompanied by elevated cytoskeletal tension, and substantiate that integrin α5β1 is a key contributor to FN assembly [255]. Yet, after 2000s, drastically fewer studies document the tumor-suppressive ability of FN [256–258]. Zoppi et al. [259] reported the effect of FN13 peptide, a 13-amino-acid human FN peptide, in inhibiting tumor-cell invasion via the modulation of integrin α5β1 organization and ILK inactivation. In tumors, these adhesive and mechanical cues collectively support invasive programs, including stromal interaction, local invasion, and extravasation [260].

Besides FN, laminins are large heterotrimeric glycoproteins that are major components of BM and play essential roles in BM formation and structural integrity [261]. Laminins, particularly laminin-332 (LM-332), also play central roles in regulating tumor-cell adhesion, migration, and survival within the BM-rich microenvironment [262]. Laminins support cell–ECM attachment through binding to integrins α3β1 and α6β4, thereby influencing cell spreading, motility, and invasive behavior [263–265]. LM-332-driven activation of α6β4 integrin/Rac/nuclear factor κ-light-chain-enhancer of activated B cells (NF-κB) signaling promotes survival and resistance to anoikis, enabling tumor cells to remain viable during detachment and dissemination [266]. In addition, CD151-mediated regulation of α3β1 and α6β4 interactions with LM-332 enhances tumor-cell adhesion and motility on the ECM [264], while the 67-kDa laminin receptor further contributes to invasive and metastatic behavior [267]. Together, these findings indicate that laminins are not only structural BM components but also active regulators of adhesive signaling and tumor-cell motility.

#### Tumor-associated remodeling of FN and laminin signaling

During tumor progression, FN and laminin signaling are extensively remodeled to favor invasion, stromal activation, and metastatic dissemination. Although some early studies suggested context-dependent tumor-suppressive effects of peri-FN, the prevailing evidence indicates that in established cancers FN predominantly acquires a pro-adhesive, pro-migratory, and pro-metastatic role [261]. Tumor-associated changes in FN expression, deposition, and assembly are regulated by multiple oncogenic and inflammatory pathways, including TNF-α/activator protein 1 (AP1)/NF-κB, human papillomavirus E2 protein (HPV E2), ribosomal S6 kinase 2 (RSK2), and von Hippel–Lindau (VHL) tumor suppressor-linked mechanisms, illustrating that FN dynamics are tightly integrated with tumor signaling networks. Increased stromal FN, often driven by endothelial leakage, macrophage-mediated inflammation, and fibroblast reprogramming, enhances tumor-cell invasion, extravasation, and metastatic colonization [268]. Likewise, laminin signaling is altered in tumors through changes in BM composition and receptor activity.

Increased FN abundance within the tumor stroma, often driven by tumor-associated fibroblasts and endothelial leakage, has been linked to enhanced intravasation and metastatic spread, whereas elevated circulating or urinary FN levels correlate with advanced disease in several malignancies, including renal cell carcinoma and CRC. Consequently, FN expression appears to be preferentially associated with Th1 cells, potentially facilitating the recruitment of additional immune populations, including macrophages and other Th1 lymphocytes, thereby promoting immune cell migration [269]. In contrast, T helper 2 (Th2) cells show little to no interaction with FN, underscoring the selective association of FN with the Th1 immune phenotype [238,270]. TNF-α secreted by macrophages induces ECM remodeling through AP1- and NF-κB-mediated FN suppression. Similarly, another mechanism identified in head and neck tumors revealed that HPV early protein 2 acts as an inhibitor of FN transcription [271]. Gawecka et al. [257] reported that RSK2 protein of the Ras/Raf pathway suppresses integrin activation and FN assembly and participates in a feedback loop in controlling FN–integrin interactions. High circulating and urinary FN levels are observed in advanced metastatic renal cell carcinoma and CRC, supporting their use as noninvasive prognostic biomarkers [272,273]. In addition, α-1 antitrypsin (A1AT) may promote tumor-cell invasion, migration, and metastatic colonization by regulating peri-FN assembly [274]. In lung adenocarcinoma, A1AT up-regulation has been associated with metastasis and poor survival, potentially through FN–integrin α5-mediated adhesion [275]. In breast cancer, FN accumulation has been associated with tumor progression and metastatic disease, while altered A1AT/SERPINA1 expression has also been reported [276,277]. Another study demonstrated that up-regulation of discoidin domain receptor 2 in Twist family BHLH transcription factor 1 (TWIST1) promoted ovarian cancer metastasis, which, in turn, increased FN cleavage and the activity of matrix-remodeling enzymes, thereby elevating the metastatic and migratory potential of tumor cells [278]. In line with its metastasis-promoting functions, FN up-regulates the long noncoding RNA (lncRNA)–FOXF1 adjacent noncoding developmental regulatory RNA (FENDRR), which correlates with poor prognosis in gastric malignancies [279]*.* Moreover, the interaction between survivin and X-linked inhibitor of apoptosis (XIAP) activates NF-κB signaling, leading to increased FN expression, β1-integrin signaling, and activation of the cell-motility kinases FAK and Src, thereby promoting tumor-cell invasion and metastasis [280].

LM-332, laminin-γ2 (LM-γ2), and the 67-kDa laminin receptor contribute to invasive growth, metastatic potential, and EMT-related programs. LM-332 activates the α6β4 integrin/Rac/NF-κB pathway, providing a survival signal to cancer cells and preventing anoikis [281]. Particularly in glioblastoma and bladder cancer, LM-332 up-regulates integrin α6β4/Notch1 signaling to promote tumor progression by disrupting the antitumor immune cascade [263]. Tetraspanin molecules regulate binding of integrins to LM-332. Tetraspanin CD151 is believed to strengthen the cell attachment to ECM by binding with integrins α3β1 and α6β4 [282,283]. Li et al. [284] reported the influence of LM-γ2 in mediating T cell exclusion. Emerging evidence demonstrates that glycosyltransferases, including GnT-III, GnT-V, and fucosyltransferase 8 (FUT8), modify their laminin receptor activity, contributing to EMT and oncogenic progression [285]. Therefore, LM-γ2 can serve as a biomarker to inform cancer treatment strategies [286]. Thus, tumor-associated remodeling of FN and laminin pathways reinforces matrix-dependent signaling circuits that support aggressive tumor behavior.

#### Roles of FN and laminins in immune surveillance evasion

In tumors, FN is frequently enriched within the desmoplastic stromal regions and along the aberrant neovasculature, where it is largely produced and organized by CAFs [287]. This FN-rich matrix not only supports tumor-cell adhesion and directional migration but also restructures the stromal architecture into a dense, permissive scaffold that limits efficient immune cell access to malignant nests [40]. Likewise, laminins regulate the interface between tumor cells, stromal cells, endothelial barriers, and infiltrating leukocytes. When laminin composition is altered during tumor progression, BMs shift from being protective tissue boundaries to becoming specialized immunomodulatory barriers that hinder antitumor immunity [233]. Together, FN and laminins create biochemical and biophysical conditions that reshape leukocyte trafficking, prevent T cell access, favor immune exclusion, persist in suppressive myeloid programs, and confer resistance to immunotherapy. FN accumulation in the tumor stroma, supported by abnormal vascular permeability and fibroblast activation, facilitates the influx of plasma-derived matrix proteins and promotes a tumor-permissive niche associated with protumor leukocyte recruitment [288]. FN promotes immune evasion by organizing a dense stromal network that spatially excludes effector lymphocytes [88]. CAF-derived FN fibrils form aligned tracks that support tumor invasion while increasing matrix density and mechanical stiffness, thereby separating CD8^+^ T cells from tumor nests and reinforcing the immune-excluded phenotype [199]. FN also accumulates in perivascular regions, where it contributes to abnormal vascular remodeling and a dysfunctional, hyperpermeable vasculature that does not support efficient cytotoxic T cell entry into the tumor parenchyma [289]. Thus, FN-rich stroma acts as an active barrier that directs tumor expansion while limiting effective immune cell infiltration.

Beyond its structural role, FN can directly promote immune suppression through the FN–immunoglobulin-like transcript 3 (FN-ILT3) axis, a stromal checkpoint pathway in which FN engages the inhibitory receptor ILT3 on myeloid cells [290]. This interaction sustains tolerogenic myeloid states, suppresses antitumor immune activation, and weakens T cell priming and effector function. FN also promotes immune escape by enhancing integrin signaling, matrix tension, and stromal mechanotransduction [88]. Through receptors such as α5β1 and αv integrins, FN-rich matrices increase cytoskeletal tension and matrix remodeling, generating a dense and mechanically restrictive stroma. This limits efficient T cell migration and reduces immune–tumor contact, allowing FN to support immune evasion through both biochemical signaling and biophysical barrier formation.

FN has been linked to selective interactions with Th1-associated immune populations, suggesting that it can influence immune cell positioning within the ECM [291,292]. In tumor niche, FN-associated vascular remodeling can promote blood vessel hyperpermeability, resulting in protumor leukocyte recruitment and a high influx of FN and fibrin-like molecules into the tumor site [293]. These changes can subsequently facilitate tumor intravasation [294]. In addition, tumor-derived secretory cues promote the emergence of a distinct fibroblast subset enriched in FN production, further contributing to immune evasion during malignant progression [295]. Elevated FN levels have been reported as prognostic biomarker of advanced-stage metastatic renal cell carcinoma, pancreatic cancer, and CRC [296].

Laminin also influences stromal interactions, modulating TME dynamics, including angiogenesis, FN-synthesizing fibrogenic fibroblast activation, immune cell infiltration, and immune evasion [233]. LM-γ2 has been implicated in T cell exclusion, as CAF-derived TGF-β1 induces its expression through c-Jun N-terminal kinase (JNK)/AP1 signaling, which disrupts T cell infiltration into the tumor bed. Similarly, laminin-511 strengthens endothelial junctional integrity and restricts leukocyte extravasation. It was reported that TGF-β1 secreted by CAFs transcriptionally activates LM-γ2 via JNK/AP1 signaling that impedes T cell infiltration, further reshaping stromal topology and chemotactic cues to keep CD8^+^ T cells away from tumor-cell clusters [284]. Accordingly, high LM-γ2 expression has been linked to poor prognosis and reduced anti-PD-1 efficacy, particularly in lung and esophageal cancers [297]. Similarly, LM-332-driven α6β4/Notch1 signaling has been associated with tumor progression and disruption of antitumor immune responses in cancers such as glioblastoma and bladder cancer [298]. Laminins also indirectly promote immune escape by enhancing tumor-cell survival, polarity changes, migration, and invasion through receptors such as integrins and dystroglycan [233]. During tumor progression, altered laminin expression, proteolytic cleavage, and chain redistribution, particularly involving LM-332, promote invasive matrix remodeling and tumor–stromal crosstalk. These changes can also sustain the spatial separation of tumor cells from antitumor lymphocytes, thereby contributing to primary resistance to ICB [299]. Collectively, these findings show that FN and laminins are not only regulators of tumor-cell behavior but also active participants in immune evasion by establishing ECM states that hinder immune surveillance and favor tumor persistence.

### MMPs and their effect on ECM remodeling and immune evasion

ECM remodeling is a necessary process, and ECM MMPs play a vital role. MMPs are a group of calcium and zinc-dependent proteolytic endopeptidases that promote the evasion of antitumor immune responses, primarily by degrading the ECM, modulating immune cell infiltration and activity, and critically influencing the TME [300]. The structure of MMPs typically consists of several distinct domains [301]. There are 6 groups of MMPs: collagenases (MMP-1, MMP-8, MMP-13, MMP-18), gelatinases (MMP-2, MMP-9), stromelysins (MMP-3, MMP-10, MMP-11), matrilysins (MMP-7, MMP-26), membrane-type MMPs, and macrophage metalloelastase. MMPs and their inhibitors have multiple biological functions at various stages of cancer development. Foremost, MMPs have the potential to create a suitable tumor niche that supports the initiation of a primary tumor and assists in neovascularization [301]. MMPs promote immune evasion through diverse but interconnected mechanisms, including ECM remodeling, chemokine processing, cytokine-receptor modulation, and suppression of antitumor immune cell activity [300]. Rather than acting as passive degradative enzymes, MMPs actively reshape the TME to favor immune escape.

MMP-1 has recently been implicated in tumor–immune crosstalk through scRNA-seq and spatial transcriptomics (ST) analyses, which identified MMP-1-associated cell subsets as drivers of macrophage enrichment and CD8^+^ T cell dysfunction. These studies highlighted the CXCL16–CXCR6 and ANXA1–FPR3 signaling axes as important mediators of an immunosuppressive TME [302].

MMP-2 exerts immunomodulatory effects at multiple levels. In lymphoma and melanoma, MMP-2 expressed by DCs and TILs promotes Th2-skewed immune responses. Mechanistically, antigen-associated MMP-2 degrades the IFN-α/β receptor on immature DCs, thereby favoring OX40L-dependent signaling during DC maturation [303]. MMP-2 has also been reported to inhibit the IL-12 subunit p35 (IL-12α), thereby weakening Th1-associated antitumor immunity [304]. More broadly, together with MMP-9 and MMP-13, MMP-2 contributes to ECM degradation and remodeling, generating a microenvironment that supports tumor immune evasion [154].

MMP-7 contributes to immune escape in CRC by impairing Fas/FasL-mediated apoptosis. Wang et al. [305] showed that in SW480, HCT15, and HT29 CRC cells, pretreatment with MMP-7 reduced apoptotic responses triggered either by CH11-mediated Fas activation or by coculture with Fas ligand-expressing Jurkat cells. In patient samples, a high MMP-7/low Fas expression pattern correlated with poor survival, indicating that MMP-7-mediated loss of Fas responsiveness is a clinically relevant immune-evasion mechanism in CRC [305].

MMP-8 appears to have context-dependent functions. On the one hand, MMP-8, together with MMP-9, proteolytically activates the neutrophil-attracting chemokines CXCL5, CXCL6, and CXCL8, thereby amplifying CXCR1/2-dependent neutrophil recruitment and activation. Concurrent ECM remodeling by MMP-8 and MMP-9 further facilitates neutrophil infiltration into tumors. On the other hand, MMP-8 has also been reported to exert tumor-suppressive effects in both in vitro and in vivo settings [304], indicating functional duality depending on tumor context.

MMP-9 is one of the most extensively studied MMPs in tumor-associated immune remodeling. In pancreatic cancer, elevated MMP-9 has been associated with enhanced immune cell infiltration and has been proposed as a potential prognostic biomarker [306]. MMP-9 also promotes immune evasion by cleaving the osteopontin isoform OPN-32 kDa, which drives MDSC expansion [307]. MMP-11 has emerged as another important immune-evasion mediator in CRC. Pan et al. [308] reported that MMP-11 promotes tumor immune escape by altering the TME and increasing slug protein expression. Vaccination against MMP-11 could elicit antitumor responses in a colon adenocarcinoma mouse model, supporting its potential as an immunotherapeutic target [309]. MMP-13, although less well characterized in this context, acts together with MMP-2 and MMP-9 in ECM degradation and remodeling, thereby contributing to the structural and signaling changes that favor immune escape [154].

MMP-14 plays a central role in cancer invasion and metastasis by degrading pericellular ECM [300]. In addition to direct matrix remodeling, MMP-14 can promote tumor progression by modifying cell-surface cytokine receptors and adhesion molecules, or through proteolytic processing of additional substrates that facilitate invasion and metastatic spread [310]. Collectively, these findings show that MMPs do far more than degrade matrix proteins. They reprogram the TME by altering chemokine gradients, cytokine signaling, immune cell recruitment, and death receptor responsiveness, ultimately disrupting ECM homeostasis and promoting a progressively immunosuppressive tumor niche [311].

## Signaling Pathways and Molecular Mechanisms Involved in Immune Evasion

**Fig. 5.**
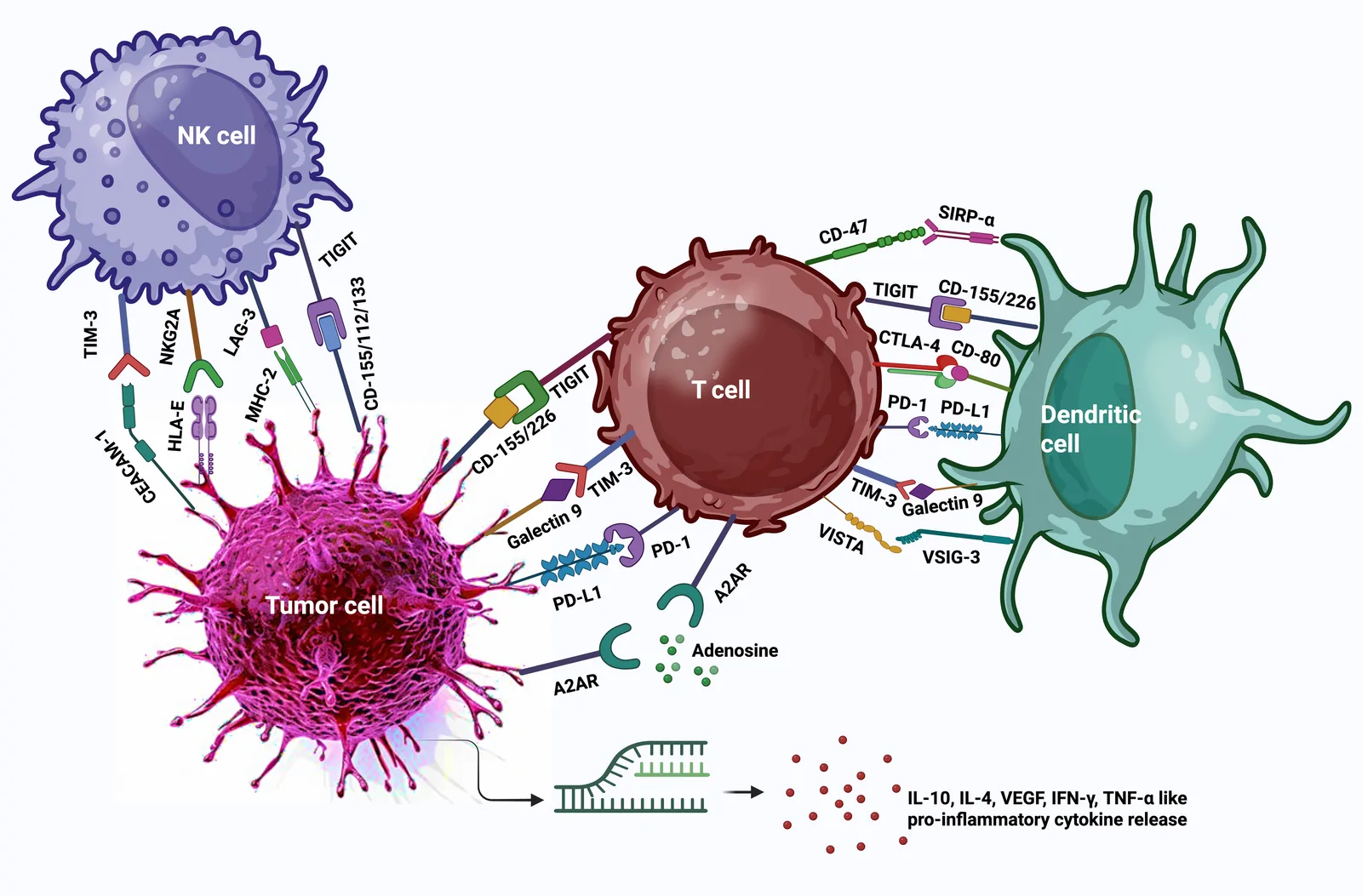
Immune-checkpoint networks mediating tumor immune escape across tumor cells, T cells, NK cells, and dendritic cells. The figure illustrates multiple inhibitory receptor–ligand interactions through which tumor cells suppress antitumor immune surveillance and reshape immune-cell function within the TME. Tumor cells express several immune-evasion ligands, including PD-L1, Galectin 9, CD155/CD112, CEACAM-1, HLA-E, MHC-I, and CD47, which engage inhibitory receptors on cytotoxic lymphocytes and antigen-presenting cells. In T cells, PD-1–PD-L1 signaling inhibits T cell receptor-dependent activation, proliferation, cytokine production, and cytotoxic effector function. Additional inhibitory axes, including TIM-3–Galectin 9, TIGIT–CD155/CD112, A2AR–adenosine, and VISTA/VSIG-3, further reinforce T cell exhaustion, reduce effector activity, and promote immune tolerance. The figure also highlights checkpoint interactions that suppress NK cell function. Tumor-expressed HLA-E binds NKG2A, MHC-I engages LAG-3, CEACAM-1 interacts with TIM-3, and CD155/CD112/CD113 bind TIGIT, collectively limiting NK cell activation, cytotoxic granule release, and tumor-cell killing. In parallel, tumor-expressed CD47 interacts with SIRP-α on dendritic cells and other myeloid cells, delivering a “don’t-eat-me” signal that reduces phagocytosis and antigen presentation. Dendritic cell-associated checkpoint interactions are also depicted, including CTLA-4–CD80, TIGIT–CD155/CD226, PD-1–PD-L1, TIM-3–Galectin 9, VISTA–VSIG-3, and CD47–SIRP-α, emphasizing how inhibitory signaling can impair dendritic cell maturation, antigen presentation, and T cell priming. The lower panel indicates that tumor-cell–derived regulatory programs and secreted mediators further support immune escape. Tumor cells can release immunomodulatory cytokines and inflammatory factors, including IL-10, IL-4, VEGF, IFN-γ, and TNF-α, which influence checkpoint expression, immune-cell recruitment, and functional polarization. A2AR, adenosine 2A receptor; CD47, cluster of differentiation 47; CD80, cluster of differentiation 80; CD112/CD133, cluster of differentiation 112/cluster of differentiation 113; CD155/CD226, cluster of differentiation 155/cluster of differentiation 226; CEACAM-1, carcinoembryonic antigen-related cell adhesion molecule 1; CTLA, cytotoxic T lymphocyte-associated antigen 4; HLA-E, human leukocyte antigen E; IFN-γ, interferon-γ; IL-4, interleukin-4; IL-10, interleukin-10; LAG-3, lymphocyte activation gene 3; MHC-2, major histocompatibility complex 2; NK cell, natural killer cell; NKG2A receptor, natural killer group 2 member A; PD-1, programmed cell death protein 1; PD-L1, programmed death protein ligand 1; SIRP-α, signal regulatory protein α; TIGIT, T cell immunoreceptor with immunoglobulin and immunoreceptor tyrosine-based inhibitory motif domains; TIM-3, T cell immunoglobulin and mucin-domain containing-3; TNF-α, tumor necrosis factor; VEGF, vascular endothelial growth factor; VISTA, V-domain immunoglobulin suppressor of T cell activation; VSIG-3, V-set and immunoglobulin domain-containing protein 3. The figure was created using BioRender.

**Fig. 6.**
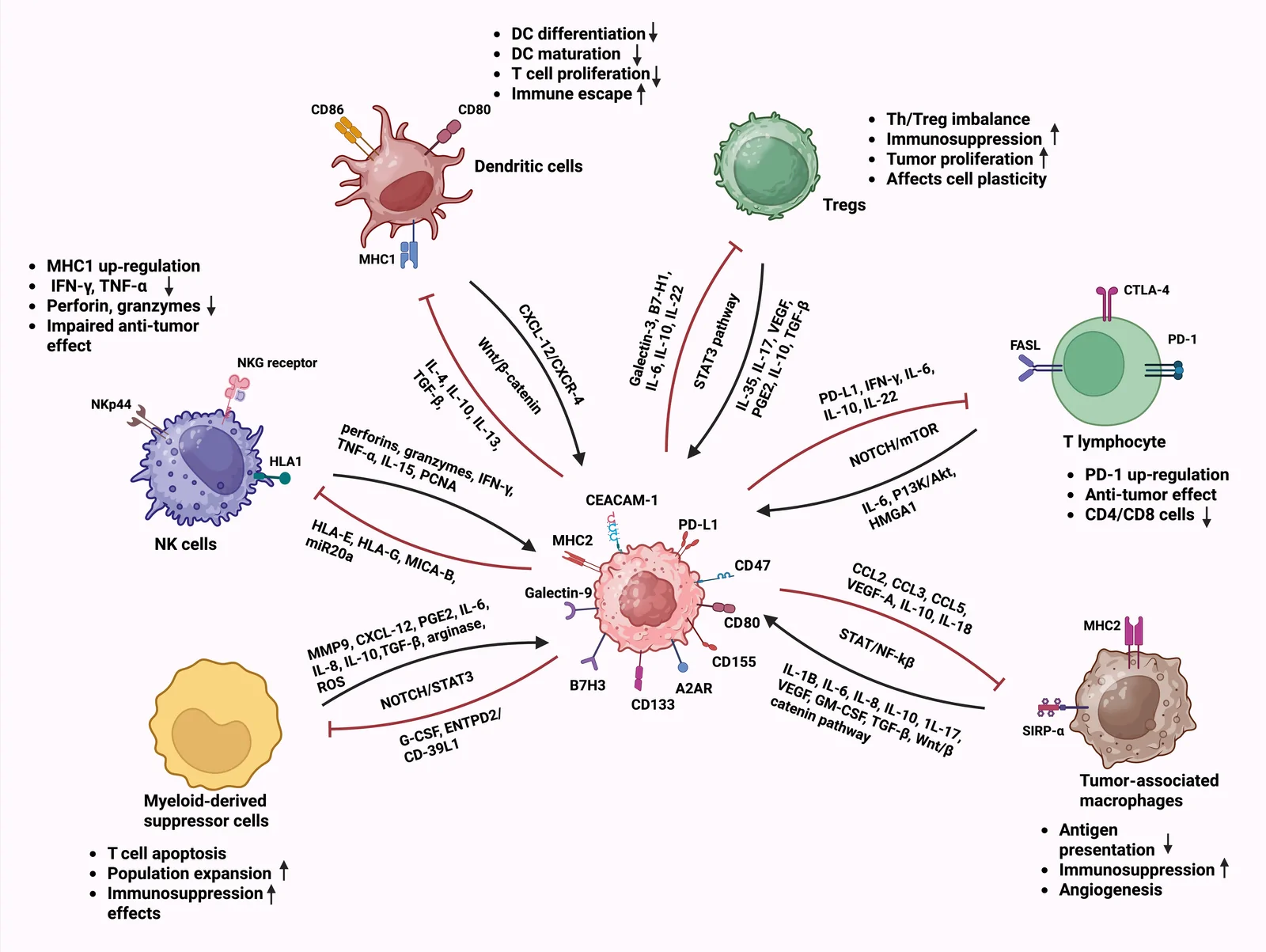
Immune-checkpoint networks and cellular crosstalk driving tumor immune escape. The schematic summarizes how tumor cells evade antitumor immunity through reciprocal interactions with major immune-cell populations in the TME. The central tumor cell expresses multiple inhibitory ligands and immunomodulatory molecules that suppress cytotoxic lymphocytes, NK cells, macrophages, DCs, and other immune populations. Red blunt-ended arrows indicate inhibitory or immunosuppressive signals directed from tumor cells toward immune effector cells, whereas dark arrows indicate reciprocal tumor–immune communication that can reinforce tumor survival, immune tolerance, and immune escape. Tumor-expressed PD-L1/PD-L2 bind PD-1 on cytotoxic T cells, exhausted T cells, regulatory T cells, B cells, DCs, macrophages, and NK cells, thereby reducing T cell receptor signaling, proliferation, cytokine production, and cytotoxic activity. The CTLA-4–CD80/CD86 axis limits T cell costimulation by competing with CD28 for binding to CD80/CD86 on APCs contributing to impaired T cell priming and expansion. Tumor or stromal expression of Fas ligand may engage Fas/CD95 on activated T cells and promote apoptosis of antitumor lymphocytes. In parallel, altered antigen-presentation machinery, including reduced MHC-I expression and impaired peptide presentation, weakens recognition by TCRs on cytotoxic T lymphocytes. The figure also highlights innate immune-checkpoint pathways. Tumor-cell CD47 interacts with SIRPα on macrophages and DCs to deliver a “do-not-eat-me” signal that suppresses phagocytosis and antigen presentation. Tumor expression of HLA-E engages NKG2A/CD94 on NK cells and subsets of cytotoxic T cells, inhibiting degranulation and interferon-γ production. Additional inhibitory receptor–ligand interactions, including TIGIT–CD155/CD112, LAG-3–MHC-II, TIM-3–galectin-9/phosphatidylserine, and VISTA-dependent signaling, further contribute to T cell exhaustion, NK cell dysfunction, and myeloid immune suppression. The surrounding immune cells represent key cellular targets of these suppressive pathways: Cytotoxic T lymphocytes lose effector function through checkpoint signaling and reduced antigen recognition; dendritic cells exhibit impaired maturation, antigen presentation, and costimulatory activity; NK cells show reduced cytotoxicity through inhibitory receptor engagement; macrophages are shifted toward immunosuppressive and pro-tumor phenotypes through CD47–SIRPα and cytokine-mediated signaling; regulatory T cells reinforce immune tolerance through CTLA-4, PD-1, and suppressive cytokines; and MDSCs contribute to T cell inhibition through arginase, ROS, NO, and immunosuppressive cytokines. Collectively, these coordinated checkpoint and cytokine networks establish an immune-excluded and immune-suppressed TME that allows tumor cells to escape immune surveillance and resist immunotherapy. A2AR, adenosine A2A receptor; AKT, protein kinase B; B7-H1, B7 homolog 1/programmed death-ligand 1; B7-H3, B7 homolog 3; CCL2, C-C motif chemokine ligand 2; CCL3, C-C motif chemokine ligand 3; CCL5, C-C motif chemokine ligand 5; CD4, cluster of differentiation 4; CD8, cluster of differentiation 8; CD39L1, CD39 ligand 1; CD47, cluster of differentiation 47; CD80, cluster of differentiation 80; CD86, cluster of differentiation 86; CD133, cluster of differentiation 133; CD155, cluster of differentiation 155; CEACAM-1, carcinoembryonic antigen-related cell adhesion molecule 1; CTLA-4, cytotoxic T lymphocyte-associated protein 4; CXCL-12, C-X-C motif chemokine ligand 12; CXCR-4, C-X-C motif chemokine receptor 4; DC, dendritic cell; ENTPD2, ectonucleoside triphosphate diphosphohydrolase 2; FASL, Fas ligand; G-CSF, granulocyte colony-stimulating factor; GM-CSF, granulocyte-macrophage colony-stimulating factor; HLA-E, human leukocyte antigen-E; HLA-G, human leukocyte antigen-G; HLA1, human leukocyte antigen class I; HMGA1, high-mobility group AT-hook 1; IFN-γ, interferon-γ; IL-1B, interleukin-1β; IL-4, interleukin-4; IL-6, interleukin-6; IL-8, interleukin-8; IL-10, interleukin-10. The figure was created using BioRender.

The ECM is increasingly recognized as an active regulator of tumor immune evasion rather than a passive structural scaffold. Through interactions with cell-surface receptors and downstream signaling pathways, the remodeled ECM influences tumor-cell behavior, stromal activation, and immune cell function [2]. Immune escape in the TME is sustained by multiple inhibitory receptor–ligand interactions involving tumor cells, T cells, NK cells, and APCs. Classical checkpoints, such as PD-1/PD-L1 and CTLA-4/CD80, suppress T cell activation and effector function, whereas additional inhibitory axes, including TIM-3/galectin-9 [312], TIGIT/CD155/CD226 [313], LAG-3/MHC-II [314], VISTA/VSIG-3 [315], A2AR/adenosine [316], CD47/SIRPα [317], and HLA-E/NKG2A (natural killer group 2 member A) [318], further dampen cytotoxic immunity and innate immune surveillance. These pathways are reinforced by tumor-derived cytokines and soluble mediators such as IL-10, IL-4, VEGF, IFN-γ, and TNF-α, which shape an immunosuppressive microenvironment and contribute to immune dysfunction [319]. The major checkpoint interactions between tumor cells, DCs, T cells, and NK cells are summarized in Fig. 5. Similarly, within the TME, cancer cells interact with multiple immune cell populations, including T cells, NK cells, DCs, macrophages, neutrophils, and MDSCs, through checkpoint receptors, cytokines, metabolic mediators, and contact-dependent signaling. These bidirectional interactions reduce antigen presentation, impair cytotoxic T cell and NK cell activity, promote immune cell exhaustion, and favor the accumulation of immunosuppressive populations. As summarized in Fig. 6, such interconnected immunosuppressive circuits collectively shift the TME from immune surveillance toward immune tolerance and tumor progression.

### ECM-associated receptor signaling in tumor progression

**Fig. 7.**
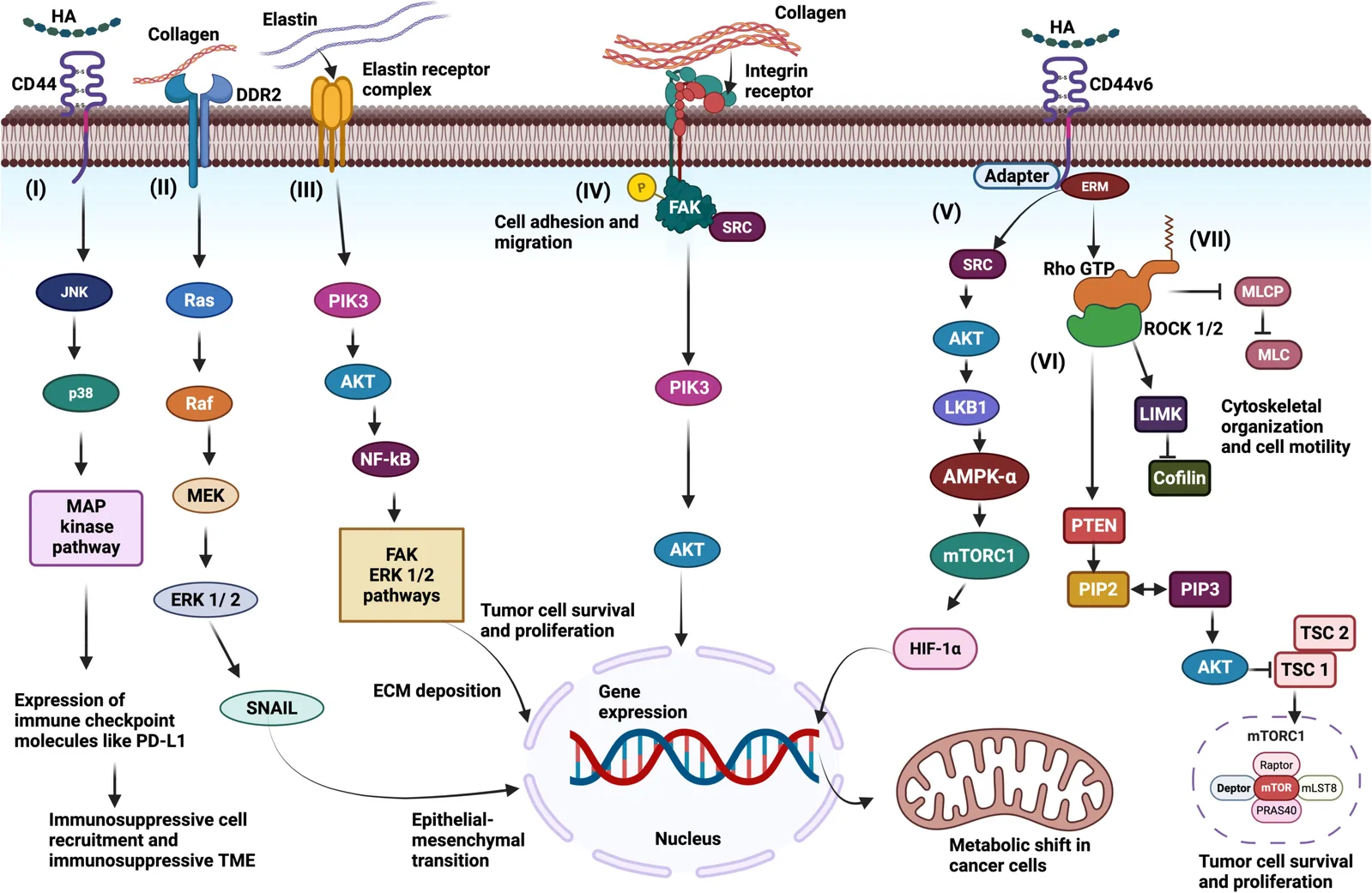
Extracellular matrix-mediated signaling pathways regulating tumor-cell behavior. The schematic illustrates representative ECM ligand–receptor interactions and their downstream signaling in tumor cells. (I) Binding of HA to CD44 activates JNK and p38, converging on the MAP kinase pathway to enhance expression of immune-checkpoint molecules such as PD-L1, thereby promoting recruitment of immunosuppressive cells and establishment of an immunosuppressive TME. (II) Collagen/DDR2 signaling activates the Ras/Raf/MEK/ERK 1/2 cascade and induces SNAIL, thereby favoring EMT. (III) Elastin signaling through the elastin receptor complex activates PI3K, followed by AKT and NF-κB, which contribute to activation of FAK/ERK 1/2-related pathways, ECM deposition, and protumorigenic transcriptional programs. (IV) Collagen–integrin receptor engagement activates FAK and SRC, thereby promoting cell adhesion and migration, while downstream PI3K/AKT signaling regulates gene expression programs associated with tumor-cell survival and proliferation. (V) HA/CD44v6 signaling, through adapter proteins and ERM proteins, activates SRC/AKT/LKB1/AMPK-α/mTORC1/HIF-1α-associated signaling, thereby supporting metabolic rewiring in cancer cells. (VI) In parallel, CD44v6-associated ERM signaling activates Rho GTP and ROCK 1/2, which further inhibit MLCP and MLC that regulate cytoskeletal dynamics, contractility, and motility. (VII) Downstream of ROCK 1/2, signaling proceeds through LIMK and cofilin to control actin reorganization, and through inhibition of MLCP and modulation of MLC to enhance actomyosin contractility. ROCK-associated signaling is also linked to PTEN, PIP2/PIP3, AKT, and the TSC1/TSC2/mTORC1 axis, which further supports tumor-cell survival and proliferation. Collectively, these ECM-driven pathways demonstrate how ECM component-dependent signaling integrates mechanotransduction, migration, metabolism, EMT, immune suppression, and growth-promoting transcriptional outputs in cancer. AKT, protein kinase B; AMPK-α, AMP-activated protein kinase α; CD44, cluster of differentiation 44; CD44v6, cluster of differentiation 44 variant 6; DDR2, discoidin domain receptor 2; ECM, extracellular matrix; ERK 1/2, extracellular signal-regulated kinase 1/2; ERM, ezrin/radixin/moesin; FAK, focal adhesion kinase; HA, hyaluronic acid; HIF-1α, hypoxia-inducible factor 1α; JNK, c-Jun N-terminal kinase; LKB1, liver kinase B1; LIMK, LIM domain kinase; MAP Kinase, mitogen-activated protein kinase; MEK, mitogen-activated protein kinase kinase; MLC, myosin light chain; MLCP, myosin light chain phosphatase; mLST8, mammalian lethal with SEC13 protein 8; mTOR, mechanistic target of rapamycin; mTORC1, mechanistic target of rapamycin complex 1; NF-κB, nuclear factor κB; p38, p38 mitogen-activated protein kinase; PD-L1, programmed death-ligand 1; PI3K, phosphoinositide 3-kinase; PIP2, phosphatidylinositol 4,5-bisphosphate; PIP3, phosphatidylinositol 3,4,5-trisphosphate; PRAS40, proline-rich AKT substrate of 40 kDa; PTEN, phosphatase and tensin homolog; Raf, rapidly accelerated fibrosarcoma kinase; Raptor, regulatory-associated protein of mTOR; Ras, rat sarcoma viral oncogene homolog; Rho GTP, Rho guanosine triphosphate; ROCK1/2, Rho-associated coiled-coil-containing protein kinase 1/2; SNAIL, snail family transcriptional repressor; SRC, SRC proto-oncogene/nonreceptor tyrosine kinase; TME, tumor microenvironment; TSC 1/2, tuberous sclerosis complex 1/2. The figure was created using BioRender.

ECM components actively regulate tumor behavior and immune escape through receptor-mediated signaling rather than acting solely as structural scaffolds. Collagen and other ECM components can engage cell-surface receptors, including integrins, discoidin domain receptor 2 (DDR2), CD44, and elastin receptor complexes. These interactions activate various downstream signaling pathways that regulate cell adhesion, migration, survival, proliferation, cytoskeletal remodeling, EMT, ECM deposition, and metabolic reprogramming. They can also influence immune regulatory programs such as PD-L1 expression and recruitment of immunosuppressive cells [320] (Fig. 7). Among these, integrin-mediated activation of FAK and Src serves as a major upstream signaling hub, with downstream convergence on PI3K/AKT, MAPK/ERK, and Rho guanosine triphosphatase (GTPase)–ROCK pathways [321]. These cascades coordinate actomyosin contractility, transcriptional adaptation, ECM deposition, and invasive cell behavior [322]. In physiological tissues, these signaling networks are tightly regulated to preserve tissue homeostasis, whereas in tumors their persistent activation promotes stromal remodeling and malignant progression [320]. Negative regulators such as PTEN normally constrain these responses, but this balance is frequently disrupted in cancer [323]. Collectively, these pathways establish ECM signaling as a central mechanistic axis through which the TME promotes cellular plasticity, invasion, and disease progression.

### ROCK and related cytoskeletal pathways in ECM-driven invasion

Among the major downstream effectors of ECM signaling, the Rho GTPase–ROCK pathway plays a central role in controlling cytoskeletal dynamics and cell motility [324]. ROCK enhances myosin light chain phosphorylation by inhibiting myosin light chain phosphatase, thereby promoting actomyosin contractility and migratory behavior. Additionally, ROCK activates LIMK1, which phosphorylates and inactivates cofilin, an actin-depolymerizing factor, thereby modulating actin polymerization and trafficking through Rac1 and mDia signaling [325]. Through additional interactions with Rac1, mDia, adducin, and ERM proteins, ROCK signaling integrates mechanical inputs from the ECM into invasive cellular responses [326]. Dysregulation of this pathway has been implicated in aggressive tumor phenotypes, particularly in squamous cell carcinoma and pancreatic ductal adenocarcinoma (PDAC) [327]. Similarly, collagen-rich ECM can activate DDR2, further reinforcing MAPK and PI3K signaling [328]. Collectively, these findings position ROCK-centered mechanotransduction as a key molecular bridge between aberrant ECM remodeling and the acquisition of invasive, tumor-promoting phenotypes.

### Tumor-driven immunosuppressive remodeling of the microenvironment

Beyond promoting proliferation and invasion, tumor cells exploit ECM-remodeled microenvironments to establish potent immunosuppressive characteristics [329]. They up-regulate inhibitory ligands, such as PD-L1, CD47, B7-H3, galectin-9, CD155, and CEACAM1, while simultaneously releasing cytokines, chemokines, and metabolites including IL-6, IL-10, TGF-β, VEGF, prostaglandin E2 (PGE2), and adenosine [330]. Collectively, these signals impair DC maturation, weaken antigen presentation, suppress NK cell cytotoxicity, expand Tregs, and recruit or polarize MDSCs and TAMs [331]. As a result, CD4^+^ and CD8^+^ effector responses are progressively attenuated, and the TME becomes increasingly permissive for immune escape [332]. Rather than treating these effects as separate processes, they should be framed as part of a coordinated immunosuppressive network shaped by tumor–ECM interactions.

### PD-1/PD-L1 and CTLA-4 as major adaptive immune-checkpoint pathways

Among immune-checkpoint pathways, PD-1/PD-L1 and CTLA-4 are the major regulators of adaptive immune suppression in tumors. PD-1 is expressed on multiple immune cell populations, including T cells, B cells, Tregs, NK cells, DCs, and monocytes, and contains immunoreceptor tyrosine-based inhibitory and switch motifs that suppress lymphocyte activation [333]. The binding of tumor-expressed PD-L1 or PD-L2 to PD-1 inhibits T cell activation, proliferation, and survival, thereby promoting immune escape in an immunosuppressive microenvironment [334]. Its regulation is influenced by cytokines such as IFN-γ [335], IL-10 [336], IL-4 [337], VEGF [338], and TNF-α [339], as well as intracellular pathways including PI3K/AKT/mechanistic target of rapamycin (mTOR) [340] and MAPK [341]. ECM signaling can further enhance PD-L1 expression through integrin-dependent and super-enhancer (SE)-associated mechanisms. In this context, Ma et al. [342] identified a PD-L1/L2-SE driven by ECM-associated integrin/BRAF/TAK1/ERK/ETV4 signaling; its deletion markedly reduced PD-L1/PD-L2 expression and restored susceptibility to T cell-mediated killing. Chakravarthy et al. [343] likewise showed that PD-1/PD-L1 signaling activates Hedgehog (HH) signaling in ECM-derived CAFs in gastrointestinal carcinoma, linking this axis to immunosuppression and immunotherapy failure. Several studies have shown the influence of microRNAs (miRNAs) like miR-34a [344], miR-28 [345], miR-138 [346], miR-25 [347], miR-93 [348], and miR-106b [349] in PD-1 pathways, deciphering their roles in regulating immune escape. Similarly, several LncRNAs, such as SNHG12 [350], PMSB8-AS1 [351], FGD5-AS1 [352], MALAT1 [353], PCED1B-AS1, and CASC11, have been reported to be involved in the PD-1 up-regulation mechanism. Interestingly, exosomal noncoding small RNA Yh4 has been reported to up-regulate PD-1 through TLR7-dependent signaling by stimulating monocytes to produce cytokines that promote immune evasion [354]. PTEN has also been implicated in PD-1 regulation through the PI3K/AKT/mTOR-S6K1 axis, as shown by Parsa et al. [355]. Clinically, several anti-PD-1/PD-L1 antibodies including atezolizumab, durvalumab, and avelumab have been approved for the treatment of multiple malignancies [356].

CTLA-4 (CD152) is an inhibitory cell-surface receptor expressed predominantly on activated T cells and Tregs, where it helps limit immune-mediated damage to normal tissues [357]. Mechanistically, CTLA-4 competes with CD28 for binding to CD80 and CD86 on APCs, thereby suppressing early T cell priming, reducing IL-2 production, and limiting T cell proliferation [358]. This inhibitory effect is most prominent during the initial phase of T cell activation in lymphoid organs, where CTLA-4 restrains naive T cell responses and weakens interactions with APCs [44]. In addition, because CTLA-4 binds B7 ligands with higher affinity than CD28, it can effectively sequester and remove CD80/CD86 from the APC surface, further attenuating costimulatory signaling and T cell activation [359]. CTLA-4 also suppresses downstream signaling pathways, including MAPK, PI3K, and NF-κB, contributing to cell-cycle arrest and immune suppression [360]. Together with PD-1, CTLA-4 forms a major immune-checkpoint axis; however, while both inhibit antitumor T cell immunity, CTLA-4 acts primarily during early T cell priming, whereas PD-1 mainly suppresses effector T cell activity in peripheral tissues and within the TME [361]. Collectively, PD-1/PD-L1 and CTLA-4 act as complementary but temporally distinct checkpoint pathways that tumors exploit to suppress both the priming and effector phases of antitumor immunity, thereby sustaining immune escape and limiting therapeutic responsiveness.

### Innate immune-checkpoint pathways and NK cell evasion

In addition to adaptive immune checkpoints, tumors also evade innate immune surveillance through inhibitory pathways that suppress NK cell and macrophage activity. One important mechanism involves the nonclassical MHC molecule HLA-E, which binds inhibitory receptors such as NKG2A on NK cells and subsets of T cells, thereby dampening cytotoxic activity [362]. This pathway may be especially relevant in tumors with reduced classical MHC-I expression, where HLA-E helps preserve immune evasion despite altered antigen-presentation capacity [224]. Additional suppression is mediated by HLA-G and other NKG2 receptor-associated interactions, which further weaken NK cell-dependent tumor killing [224]. Tumors can also disrupt missing-self recognition by releasing ligands that interfere with NKG2D-mediated activation.

Another major innate checkpoint is the CD47–SIRPα axis. CD47 expression on tumor cells delivers a “do not eat me” signal to macrophages, thereby protecting malignant cells from phagocytosis [142]. A study by Jaiswal et al. [317] found that hematopoietic and leukemia cells were protected from phagocytosis and immune attack through up-regulation of CD47. At the same time, the antibody against SIRPα drastically favored the phagocytosis of CD47-expressing cancer cells [363]. Therefore, experimental blockade of this pathway has been shown to enhance phagocytosis and promote a more immune-reactive TME, including increased IFN-γ production and macrophage-dependent antitumor responses [364]. Altogether, these mechanisms show that tumors subvert innate immune checkpoints to disable NK cell cytotoxicity and macrophage phagocytosis, thereby reinforcing immune escape and expanding the therapeutic rationale for combining innate and adaptive checkpoint blockade.

### Adenosine-mediated suppression in ECM-rich tumors

Adenosine signaling represents another important immunosuppressive pathway in ECM-rich and hypoxic tumors [365]. The adenosine receptor A2AR, a G-protein-coupled receptor linked to cyclic adenosine monophosphate (cAMP)/protein kinase A (PKA) signaling, restrains immune cell activation and helps preserve tissue integrity under physiological conditions [366]. By coupling to Gαs family members, including guanine nucleotide binding protein α stimulating activity polypeptide (GNAS) and G protein subunit α (GNAL), A2ARs activate cAMP/PKA signaling to restrain immune cell responses and preserve tissue integrity [367]. In tumors, however, severe hypoxia and stromal remodeling promote extracellular adenosine accumulation, while ectoenzymes such as CD39 further amplify this effect [368]. Activation of A2AR suppresses NK cells, myeloid cells, and other immune populations, thereby reinforcing tumor-associated immune tolerance [369]. Because this pathway links hypoxia, ECM remodeling, and metabolic immune suppression, it is best considered separately from classical receptor–ligand checkpoints while remaining within the broader framework of tumor immune evasion.

### Additional inhibitory pathways reinforcing immune exhaustion

Several additional inhibitory pathways further reinforce immune dysfunction within the TME. LAG-3 is expressed on Tregs [370], NK cells [371], CD4^+^ [372] and CD8^+^ T cells [373], DCs [374], and B cells [375] and interacts with ligands such as MHC-II [376], galectin-3 [377], LSECtin [378], and fibrinogen-like protein-1 [377]. These interactions suppress IL-2 production, reduce T cell expansion, and promote immune evasion. TIM-3, which is expressed on multiple immune cell populations, is frequently coexpressed with PD-1 and contributes to T cell dysfunction and impaired Th1 polarization [165]. Increased TIM-3 expression in endothelial cells leads to the progression and dissemination of lymphoma by suppressing CD4^+^ T cells via the IL-6/STAT3 pathway and inhibiting Th1 polarization [379]. Similarly, indoleamine 2,3-dioxygenase (IDO) suppresses antitumor immunity through tryptophan depletion, while B7-H3 has been associated with reduced cytotoxic T cell activity and adverse clinical behavior in several solid tumors [380]. The mechanistic convergence between ECM signaling and immune-checkpoint activation has important therapeutic implications. Targeting integrin-FAK/Src signaling, PI3K/AKT, MAPK, or ROCK may help disrupt tumor-promoting ECM signaling [381], while blockade of PD-1/PD-L1 [382], CTLA-4 [383], NKG2A [384], CD47–SIRPα [385], A2AR [386], LAG-3 [387], TIM-3 [388], or IDO [389] may restore antitumor immunity. Together, these mechanisms establish a multilayered inhibitory system that weakens antitumor immunity, promotes tumor persistence, and underpins resistance to immunotherapy, thereby supporting the rationale for combinatorial checkpoint-targeting strategies.

## Mechanistic Insights into ECM-Mediated Immune Escape

ECM stiffness is a defining biomechanical feature of tumors and results from collagen accumulation, crosslinking, and fibrosis [390]. In cancer, alterations in ECM composition and topology increase matrix stiffness and reshape mechanotransduction signaling [391]. Tumor cells and stromal elements drive extensive ECM remodeling through matrix degradation, GAG accumulation, and deposition of FN and TN [392], promoting both mechanical and immune dysregulation [87]. These changes restructure the tumor stroma and set the stage for immune and mechanical dysregulation.

As the matrix becomes denser and more highly crosslinked, it functions not only as a physical barrier to immune cell infiltration but also as an active signaling platform that promotes immune escape [88]. Increased collagen density and fiber alignment restrict T cell trafficking, impair immune cell access to tumor nests, and favor an immune-excluded phenotype [152]. At the same time, stiff ECM activates integrin-dependent mechanotransduction pathways, including FAK, Src, YAP/TAZ, and RhoA/ROCK signaling, thereby enhancing tumor-cell survival, invasiveness, and resistance to immune attack [56].

Matrix densification also compresses blood and lymphatic vessels, reduces perfusion, and promotes hypoxia, which further sustains stromal activation, collagen crosslinking, and immunosuppressive signaling, as reflected by tumor-associated collagen signatures (TACSs) linked to invasion [393]. Within this remodeled ECM, CAFs expand, and macrophages shift toward an immunosuppressive M2-like phenotype, collectively limiting effective cytotoxic T cell function [394]. In parallel, increased collagen density and alignment generate tension-bearing migration tracks that facilitate tumor-cell invasion and intravasation, while stiffness-dependent cues alter tumor-cell morphology, proliferation, and immune-evasive behavior through mechanotransduction pathways [395]. Consistent with this, high type I collagen density is associated with poor prognosis in oral, breast, and gastric cancers [396]. Collagen fibers aligned parallel to the tumor boundary define TACS-2, whereas radial or perpendicular alignment at the invasive front characterizes TACS-3, particularly in highly invasive lesions [397]. These organized, tension-bearing collagen architectures promote directional invasion and have prognostic relevance [398]. Thus, ECM remodeling is not only a structural consequence of tumor growth but also a dynamic regulator of mechanosignaling, stromal immunosuppression, and immune evasion.

### From epigenetics to ECM mechanics: RASSF1A and Hsp47 as modulators of tumor progression

Epigenetic dysregulation is a key upstream driver of ECM remodeling in cancer. As a clinical biomarker, ras association domain family member 1A (RASSF1A) is associated with the mechanical properties of the ECM, which increase cancer stemness and metastatic spread [399]. Loss of RASSF1A expression is often associated with increased ECM stiffness, which promotes tumorigenesis by facilitating cell migration, invasion, and resistance to apoptosis [400,401]. These observations support the idea that RASSF1A functions not only as an epigenetically silenced tumor suppressor but also as a biomarker associated with the mechanical state of the tumor ECM.

#### RASSF1A silencing links epigenetic change to collagen remodeling and ECM stiffening

Mechanistically, RASSF1A silencing disrupts cytoskeletal control and promotes nuclear accumulation of YAP1, thereby enhancing mechanosensitive transcriptional programs [402]. Activated YAP1 up-regulates collagen-remodeling genes, including prolyl 4-hydroxylase α-2 (P4HA2), which promotes collagen biosynthesis and maturation [400]. P4HA2 catalyzes collagen-specific hydroxylation reactions required for triple-helix formation, proper collagen folding, and fiber stabilization [403]. As a result, collagen deposition and matrix stabilization increase, leading to a denser and stiffer ECM. This mechanically altered matrix further amplifies YAP/TAZ signaling in tumor and stromal cells, establishing a feed-forward loop that promotes EMT, stem-like traits, and metastatic dissemination. In lung adenocarcinoma, RASSF1A-associated collagen remodeling has also been linked to changes in differentiation markers such as TTF-1 and mucin 5B, highlighting the clinical relevance of this axis [400].

#### Hsp47 promotes collagen maturation and matrix deposition

Another important regulator of ECM remodeling is Hsp47, an endoplasmic reticulum collagen-specific chaperone encoded by serpin family H member 1 (SERPINH1) [404]. Hsp47 facilitates procollagen folding, maturation, secretion, and deposition, thereby supporting the assembly of a stable collagen-rich matrix. Elevated Hsp47 expression has been reported during tumor progression, and its silencing can revert invasive cancer cells toward less aggressive, noninvasive structures in 3D culture systems [405]. These findings indicate that Hsp47 is a key mediator of collagen-driven ECM remodeling in cancer.

Beyond its role in collagen maturation, Hsp47 also contributes to tumor-promoting signaling. It has been implicated in TGF-β1 induction and activation, which supports tumorigenesis and stemness [406]. Hsp47 can also activate ERK signaling and increase CCL2 production, thereby engaging the CCR2 axis and promoting tumor progression [407]. Consistent with these functions, elevated Hsp47 expression has been reported across multiple tumor types, including NSCLC [408], bladder [407], prostate [409], head and neck [410], osteosarcoma [411], colorectal [412], pancreatic [413], gastric [414], glioblastoma [415], and esophageal cancers [416]. In CRC, Hsp47 has additionally been linked to 5-fluorouracil resistance, and its inhibition improves treatment response [417].

Given its central role in collagen biosynthesis and matrix accumulation, Hsp47 represents a promising therapeutic target in tumors with pronounced desmoplasia. Experimental targeting of Hsp47 disrupts collagen-processing machinery, induces ER stress, and interferes with the production of a protumorigenic ECM [418]. Several anti-Hsp47 strategies, including pirfenidone [419], terutroban [420], and AK-778 [421], have shown antifibrotic activity. However, because Hsp47 is also required for physiological collagen homeostasis, therapeutic approaches may need to rely on tumor-selective or targeted delivery systems to minimize off-target toxicity. Together, RASSF1A and Hsp47 illustrate how epigenetic and posttranscriptional dysregulation can converge on collagen remodeling to alter ECM mechanics. By increasing collagen maturation, deposition, and stiffness, these pathways reshape mechanotransduction, stromal organization, and tumor-cell behavior, ultimately promoting progression and metastasis.

### CAF and ECM remodeling

Tumor ECM stiffness is an important determinant of response to ICIs and is strongly shaped by CAFs [422]. CAFs are recruited by GFs such as TGF-β, FGF2, and PDGF, which are secreted by tumor cells. PDGF (particularly PDGF-AA and PDGF-BB) promotes fibroblast chemotaxis and expansion through PDGF receptor (PDGFR)-α/β signaling, while FGF2 enhances fibroblast survival, motility, and ECM remodeling capacity [423]. Activated CAFs up-regulate α-smooth muscle actin (α-SMA) and vimentin, thereby acquiring a stellate myofibroblast-like morphology and developing increased cytoskeletal tension [424]. They also secrete ECM components and soluble mediators, including FN, VEGF, stromal cell-derived factor-1 (SDF1), and a broad secretome enriched in cytokines and chemokines such as IL-1β, IL-6, IL-10, CXCL1, CXCL5, and CXCL12 [425]. They then remodel the tumor stroma by promoting deposition, alignment, and crosslinking of FN, fibrillar collagens, laminin, elastin, and PGs. CAF-derived FN serves as a provisional scaffold for collagen fibrillogenesis, while Rho–ROCK–myosin II signaling and related cytoskeletal regulators further enhance matrix contraction and reorganization [426]. CAFs markedly alter the biochemical composition and physical properties of the ECM by increasing the deposition, alignment, and crosslinking of fibrillar collagens (type I and III), FN, laminin, elastin, and PGs [78]. CAF-derived FN serves as an early provisional matrix that facilitates collagen fibrillogenesis, thereby enhancing ECM stiffness and anisotropy [78]. In parallel, CAFs induce LOX/LOXL, prolyl hydroxylases, and transglutaminases, which stabilize collagen fibers and increase matrix stiffness and anisotropy [427]. A study showed that fibroblasts generate tracks within the ECM through force- and protease-mediated matrix remodeling, whereas squamous cell carcinoma cells use CDC42 and myotonic dystrophy kinase-related CDC42-binding protein kinase (MRCK) to migrate along these tracks [428]. Another study demonstrated that tensile forces drive a reversible fibroblast-to-myofibroblast transition and promote the deposition of highly stretched FN fibers. As the ECM matures into collagen-rich tissue, FN-fiber tension gradually decreases [429]. These processes collectively generate a stiff, highly organized matrix that supports tumor-cell migration, invasion, and metastatic dissemination.

### CAF heterogeneity, functional subtypes, and ECM remodeling

Recent scRNA-seq, ST, and proteomic studies have shown that CAFs cannot be defined by a single marker or functional state. Instead, they comprise diverse transcriptional and functional subpopulations shaped by local microenvironmental signals, with distinct effects on ECM remodeling and immune regulation [429]. This heterogeneity is highly relevant to tumor immune evasion because different CAF subsets promote stromal remodeling and immunosuppression through complementary mechanical and paracrine mechanisms.

Among the best-characterized subsets, COL11A1 and periostin (POSTN)-CAFs show distinct but convergent protumor functions [430]. COL11A-CAFs are strongly linked to desmoplastic ECM remodeling and immune exclusion. They promote collagen deposition, fiber alignment, and matrix entanglement, thereby increasing tissue stiffness and restricting immune cell motility [431]. This subset has also been associated with the SPP1–CD44 axis, which may further support collagen organization and the retention of immunosuppressive myeloid populations [432]. While direct SPP1–CD44 mechanisms in CAFs have not yet been fully mapped, recent cancer transcriptomic analyses show that SPP1 macrophages colocalize with COL11A1-CAF regions, and this interaction correlates with lower T cell infiltration and immunosuppressive microenvironments, suggesting a functional crosstalk relevant to immune escape [433].

In contrast, POSTN-CAFs appear to act predominantly through paracrine immunosuppressive signaling. By secreting POSTN**,** they promote stromal remodeling and activate TGF-β-dependent pathways that support the recruitment, polarization, and persistence of TAMs [434]. TAMs, in turn, suppress antitumor immunity by producing IL-10, TGF-β, and arginase-1, thereby reinforcing an immunosuppressive cytokine milieu [433]. POSTN CAFs can also contribute to ECM reorganization by enhancing FN assembly and BM disruption, further facilitating immune escape [435].

Evidence from multiple cancers supports the concept that CAF-driven immune suppression is subtype-specific. In breast cancer, Costa et al. [436] identified 4 CAF populations (CAF-S1 to CAF-S4), with CAF-S1 and CAF-S4 reported to be responsible for immunosuppression. Similarly, in PDAC, Öhlund et al. [437] have reported the first observation of diverse CAF subtypes and defined their unique gene signatures in vivo, named the “myofibroblastic CAFs” with α-SMA up-regulation localized near to neoplastic cells within the peri-glandular region, as well as “inflammatory CAFs” with relatively low α-SMA expression along with high expression of IL-1, IL-6, IL-11, and CXCL1 [437]. Similarly, the same team has identified “antigen-presenting CAFs” capable of activating CD4^+^ T cells [438]. In cholangiocarcinoma, a rarer mesothelial CAF population has also been described, which expresses mesothelial markers like Msln, Upk1b, and Upk3b [439]. Rather than listing these as isolated observations, these studies collectively indicate that CAF subsets differ in how they remodel the ECM, shape cytokine networks, and influence immune cell behavior.

The clinical importance of CAF heterogeneity lies in its association with stromal architecture, immune exclusion, and treatment response [440]. Several scRNA-seq-based studies offer insights into the dynamic interactions between the TME and CAFs [441,442]. Subtype-specific ECM programs, particularly those linked to COL11A1 and POSTN-CAFs, may help explain differences in sensitivity to ICB and suggest that selective targeting of immunosuppressive CAF states could improve therapy while sparing tumor-restraining stromal functions [431]. Several markers, including α-SMA, PDGFRα/β, fibroblast activation protein (FAP), fibroblast-specific protein 1 (FSP1), TNC, vimentin, and podoplanin (PDPN), have therefore been explored for CAF identification and stratification [443,444].

HH signaling contributes to CAF heterogeneity by supporting protumor fibroblast states with high ECM-remodeling capacity, including CAF phenotypes functionally analogous to COL11A1 and POSTN subsets. Through these stromal states, HH signaling promotes collagen deposition, matrix stiffening, POSTN-rich remodeling, and immune exclusion. Accordingly, HH inhibitors such as vismodegib and sonidegib may be interpreted as therapies that modulate CAF heterogeneity and restrain ECM-remodeling [445], tumor-supportive fibroblast programs, rather than merely suppressing fibroblast proliferation. This provides a mechanistic rationale for combining HH-targeted agents with chemotherapy or immunotherapy.

### ECM-remodeling enzymes and tumor progression

MMPs, A disintegrin and metalloproteinases (ADAMs), ADAMTS proteases, and other tissue proteases collectively drive ECM remodeling by altering matrix composition and stiffness, releasing bioactive ECM fragments, and reshaping stromal signaling networks [446]. Among MMPs, MMP-9-mediated FN cleavage modifies αvβ6 integrin dynamics, resulting in increased β6 integrin expression and enhanced tumor-cell invasion through FAK-Src-ERK1/2 and PI3K-AKT-Smad1/5/8 signaling [447]. In parallel, MMPs also regulate the bioavailability of matrix-sequestered GFs; for example, neutrophil-derived MMP-9 releases VEGF from the ECM through HS cleavage, thereby promoting angiogenesis within the tumor niche [448]. MMP-2 up-regulation by TEM1 further contributes to matrix stiffening, reinforcing the invasiveness of the remodeled stroma [447]. In contrast to MMPs, ADAM family proteases primarily modulate cell communication through ectodomain shedding of GF receptors, cytokines, and adhesion molecules, thereby influencing paracrine signaling and cell–ECM interactions [449]. ADAMTS proteases act more directly on structural ECM substrates, particularly PGs and collagens, to reorganize matrix architecture, alter tissue stiffness, and support stromal remodeling, angiogenesis, and tumor-cell motility. Additional protease families also contribute to this remodeling landscape [450]. Meprins, for instance, cleave collagens, laminins, and PGs while also processing cytokines and GFs, linking ECM turnover to inflammatory signaling [451]. Likewise, serine, aspartic, and cysteine proteases participate in matrix degradation and facilitate immune cell infiltration, further broadening the proteolytic control of the TME [452].

### ECM architecture as determinants of immune cell distribution

ECM architecture is a major determinant of immune cell distribution within tumors [151]. Features such as matrix density, stiffness, porosity, fiber alignment, and spatial heterogeneity collectively regulate where immune cells localize, how efficiently they migrate, and whether they can reach malignant cells [56]. Excessive matrix densification and stiffening reduce ECM porosity and create physical barriers that preferentially restrict the infiltration of cytotoxic CD8^+^ T cells and NK cells, thereby promoting immune exclusion [453].

Beyond acting as a physical barrier, stiff ECM also reshapes immune distribution through mechanotransduction and stromal signaling. Increased matrix rigidity alters integrin-dependent cytoskeletal dynamics in immune cells, impairing their migration and effector function, while mechanosensitive pathways such as Piezo1 further modulate immune cell behavior [454]. In parallel, ECM stiffening activates CAFs and reinforces immunosuppressive signaling, leading to increased production of TGF-β, IL-6, and related mediators that favor the recruitment and persistence of Tregs, TAMs, and MDSCs over antitumor effector populations [453]. TGF-β further amplifies this state by promoting collagen deposition, myofibroblast differentiation, and reduced matrix degradation, thereby stabilizing a fibrotic and immune-restrictive microenvironment [455].

Collagen-rich matrices are particularly important in this process because they influence both immune cell phenotype and motility. Dense or disorganized collagen networks impair T cell trafficking and reduce the ability of CD4^+^ and CD8^+^ T cells to establish effective contact with tumor cells [456]. Experimental studies further show that cytotoxic T lymphocyte migration is hindered in concentrated collagen gels and in poorly aligned 3D collagen matrices, whereas loose and well-aligned fibers better support immune cell movement [457,458]. Consistent with this, lymphocyte motility in physiological reticular ECM relies on permissive matrix organization, highlighting how tumor-associated collagen remodeling can convert a normally supportive scaffold into a barrier to immune surveillance [459]. Myeloid cells further modify this spatial landscape through active ECM remodeling. Tissue-infiltrating myeloid cells facilitate enzymatic remodeling of BM components, generating paths that can redirect or constrain lymphocyte trafficking [87]. Macrophages can contribute to collagen deposition and act as organizers of tumor-associated matrix remodeling, while neutrophils can transport and redistribute preexisting matrix material to support the formation of new ECM scaffolds [460]. These processes are relevant because they connect immune cell behavior directly to ongoing stromal remodeling, rather than treating myeloid activity as a separate phenomenon. This spatially restrictive ECM pattern is evident in tumors such as colon, pancreatic, breast, and lung cancers, where immune cells often accumulate in stromal regions surrounding tumor nests rather than penetrating deeply into the cancer cell compartment [461]. Such immune localization frequently parallels regional differences in collagen organization, with densely bundled fibers in tumor-adjacent areas creating local barriers to immune cell movement and tumor-cell contact [107]. Collectively, these observations support the concept that ECM structure acts as a spatial regulator of antitumor immunity by integrating mechanical confinement with stromal and biochemical signaling [462]. Altered ECM deposition, cross-linking, degradation, and matrix organization reshape tissue stiffness, stromal architecture, vascular accessibility, and chemokine distribution, thereby directly influencing immune cell trafficking and localization [462]. Altogether, changes convert molecular mechanisms of immune escape into spatial and functional barriers that restrict cytotoxic T cell and NK cell infiltration while favoring the accumulation of immunosuppressive populations such as Tregs, MDSCs, and TAMs.

## Tumor-Induced ECM Mechanistic Alterations and Immune Cell Infiltration in the TME

ECM remodeling promotes immune evasion through coordinated biomechanical, stromal, and vascular mechanisms. In tumors, a dense and stiff matrix not only limits immune cell access to malignant cells but also reprograms stromal and myeloid compartments toward immunosuppression, disrupts vascular trafficking, and reduces the efficacy of immunotherapy [87]. This section focuses on how tumor-induced ECM remodeling translates mechanistic immune-evasion pathways into impaired immune infiltration and sustained immunosuppression within the TME.

Compared with softer tumors, collagen-rich and mechanically stiff tissues generally exhibit reduced immune cell infiltration. Excessive collagen deposition, altered fiber organization, and reduced matrix porosity impair immune cell migration, positioning, and antigen presentation, thereby weakening productive cytotoxic interactions between effector cells and tumor cells [463,464]. Matrix stiffening also promotes TNC accumulation, further reinforcing a nonpermissive stromal architecture that restricts T cell entry into tumor nests [465]. Thus, ECM stiffening is not only a structural abnormality but also a functional determinant of immune exclusion. These biomechanical constraints have direct therapeutic consequences. In collagen-enriched tumors, ECM stiffness and porosity reduce the efficacy of CD8^+^ T cell-mediated immunotherapies by limiting intratumoral penetration and cytotoxic engagement [466]. This may partly explain why some tumors remain poorly responsive to immunotherapy despite the presence of immune cells at the invasive margin.

Beyond forming a physical barrier, the remodeled ECM actively reshapes stromal and immune cell behavior. CAFs are central to this process because they both restrict immune cell access and secrete cytokines and chemokines that alter immune recruitment and function [467]. In several tumor settings, CAF abundance correlates negatively with CD8^+^ TIL and positively with FoxP3^+^ T cells, consistent with an immunosuppressive niche, with IL-6 emerging as an important mediator of this effect [424]. Additional mechanisms, including antigen cross-presentation and PD-L2/FASL-associated suppression, further support the role of CAFs in dampening T cell activity [468]. Specialized CAF subsets may also contribute to tumor progression and immune escape through distinct signaling programs, although these examples are best interpreted within the broader framework of ECM-driven stromal remodeling rather than as isolated phenomena [469].

Myeloid compartments are similarly shaped by the remodeled matrix. Stiff and collagen-rich ECM favors macrophage polarization toward tumor-supportive phenotypes, while TAMs reciprocally enhance ECM remodeling through collagen deposition, alignment, and crosslinking [470–472]. This establishes a feed-forward loop in which ECM stiffening and macrophage-mediated immunosuppression sustain one another. Other suppressive populations, including Tregs and MDSCs, further intensify this environment by inhibiting CD8^+^ T cell and NK cell activity through TGF-β, arginase, nitric oxide (NO), and ROS [331,473].

Specialized immune cell subsets such as γδ T cells [474], invariant natural killer T (iNKT) cells [475], mucosal-associated invariant T (MAIT) cells [476], and cytokine induced killer (CIK) cells [477] are increasingly being explored in solid tumors because they combine rapid effector function with relative MHC independence; however, in ECM-rich tumors, their activity is profoundly shaped by matrix stiffness, topology, and stromal remodeling. A dense, collagen-rich, and mechanically stiff ECM can limit immune cell infiltration by reducing pore size, increasing tissue compression, and creating fibrillar barriers while also reinforcing CAF- and TAM-driven immunosuppressive programs [88]. Another group of cells that gets targeted by increased tumor ECM stiffness is the tumor endothelial cells (TECs) and the pericytes [478]. Extreme ECM stiffness promotes VEGF hyperactivation mediated by protein phosphatase 2A (PP2A), which in turn activates the secretion of TECs and YAP activity in TECs, thereby promoting endothelial cell proliferation, sprouting, and neovascularization [479]. Pericytes, which serve as mural cells of the microcirculation, are integral to the vascular wall and are embedded within type IV collagen of the microvasculature, with established involvement in cancer and other diseases [480]. Increased ECM stiffness may trigger the aberrant conversion of pericytes into fibroblasts, which, in turn, increases metastasis through YAP activation. They also remodel the perivascular environment by degrading collagen IV, making the ECM conducive to tumor metastasis [481]. Consequently, γδ T cells, iNKT cells, MAIT cells, and CIK cells not only are physically restricted from reaching tumor nests but also may exhibit reduced effector function within fibrotic and suppressive stromal niches [482]. Thus, their antitumor activity in solid tumors is strongly shaped by ECM stiffness, matrix topology, and stromal remodeling, supporting combination strategies with matrix-normalizing or stromal-targeted therapies.

ECM remodeling also constrains antitumor immunity by altering vascular and perivascular compartments. Increased matrix stiffness promotes endothelial dysfunction, aberrant angiogenic signaling, and structural disorganization of tumor blood vessels, thereby impairing leukocyte extravasation into tumor tissue [479]. These changes reinforce the immune-excluded phenotype by restricting immune cell access to the tumor core. Perivascular remodeling adds another layer of immune dysfunction. Matrix stiffening can alter pericyte behavior and destabilize the microvascular niche, while ECM topological changes may impair DC infiltration and antigen-presenting capacity [479,483,484]. Together, these findings indicate that ECM remodeling limits antitumor immunity not only within the stromal compartment but also at the level of vascular access and immune priming.

The immune consequences of ECM remodeling have clear therapeutic relevance. Strategies that reduce matrix-associated rigidity or interrupt ECM-driven stromal signaling may help restore immune infiltration and improve treatment responses [88]. Examples include IL-6 blockade [485], dual TGF-β/PD-L1 targeting [486], colchicine-based matrix modulation [487], and ECM-associated regulators such as βig-h3 and superoxide dismutase 3 (SOD3) [488], which collectively support the concept that stromal remodeling can be therapeutically manipulated to convert immune-excluded tumors into more immune-permissive states.

### ECM stiffness in the regulation of proliferation

Stiffer ECM promotes tension within the tumor cytoskeleton, characterized by enlarged cell dimensions and pseudopodial structures [395]. This tension can either activate or suppress various GFs and subsequent signaling cascades, leading to mitogenic responses within the ECM. Current findings underscore the interaction of stiffer ECM components, such as collagen deposition and FA molecules, with other regulatory factors, ultimately supporting uncontrolled cell proliferation [489]. In a study by Schrader et al. [490], matrix stiffness values of 6 and 12 kPa were linked to fibrotic and cirrhotic hepatocellular carcinoma (HCC) phenotypes, accompanied by a 12.2-fold increase in cell proliferation and elevated expression of proliferating cell nuclear antigen (PCNA) and cyclin D1. Additionally, enhanced FAK, ERK, protein kinase B, and STAT3 indicate that ECM stiffness considerably regulates cell proliferation [56]. CXCR4 acts as a major regulator of ECM stiffness and cell proliferation via YAP signaling, thereby enhancing tumor-cell proliferation by modulating cell-cycle control, DNA replication and repair, and mitosis, as supported by numerous reports [491,492].

### ECM stiffness in metabolism reprogramming and metastasis

ECM stiffness is not only a structural feature of tumors but also an active regulator of metabolic adaptation. As the matrix becomes denser and more rigid, it impairs perfusion and metabolite diffusion, thereby promoting hypoxia, extracellular acidification, and nutrient stress within the TME [493]. These physical constraints are coupled to mechanotransduction pathways, particularly those mediated by integrins, CD44, and cytoskeletal tension, which reprogram tumor and stromal cell metabolism toward states that support survival, invasion, and immune escape. Thus, the metabolic abnormalities observed in tumors not only are consequences of rapid proliferation but also are shaped by the mechanical properties of the ECM.

#### ECM stiffness and glucose metabolism

The stiffness of the ECM can trigger mechanosignaling pathways that reprogram glycolytic and mitochondrial metabolism; however, the direction of these metabolic modifications is context dependent and varies across tumor and cell types [56]. In glioblastoma, Sohrabi et al. [494] demonstrated that a softer, brain-like matrix shifted glioblastoma cells toward a glycolysis-weighted metabolic state, accompanied by reduced proliferation and increased migration, whereas a stiffer matrix favored oxidative phosphorylation and proliferation. At the level of acid-base homeostasis, tumor cells regulate intracellular and extracellular pH through the coordinated activity of proton, bicarbonate, and lactate transport systems, together with carbonic anhydrase activity [495].

#### ECM stiffness and lipid metabolism

Matrix stiffening also reshapes lipid metabolism, thereby increasing cell membrane fluidity. By altering cytoskeletal organization and membrane demands, stiff ECM promotes lipid metabolic programs that support membrane remodeling, survival signaling, and invasive behavior [7]. Through integrin-FAK signaling and cytoskeletal tension, stiff ECM also promotes lipid metabolic adaptations that support membrane remodeling, survival, and invasion [1]. This shift may further contribute to matrix dysregulation and hinder immune cell infiltration and effector function. Increased ECM stiffness promotes ubiquitin-proteasome-mediated degradation of SCD1 (stearoyl-CoA desaturase), resulting in an elevated monounsaturated-to-saturated fatty acid ratio [496]. These changes may include increased fatty acid desaturation and altered lipid composition, which help tumor cells maintain membrane plasticity under mechanical stress [497]. In this way, lipid metabolism becomes part of the broader adaptive response to a rigid matrix rather than an isolated metabolic feature.

#### ECM stiffness and amino acid metabolism

Amino acid metabolism is likewise influenced by ECM stiffness. Mechanically activated tumor and stromal cells increase their dependence on pathways such as glutamine and arginine metabolism to sustain anabolic growth, redox balance, and cytoskeletal remodeling [498]. In CAFs, these metabolic adaptations can further support matrix remodeling and metastatic behavior, creating a reciprocal relationship in which ECM stiffening drives metabolic rewiring, and the resulting metabolic state reinforces fibrosis and tumor progression [499]. Furthermore, a stiffened ECM up-regulates l-arginine, a urea cycle-derived metabolite, via a YAP-regulated creatine phosphagen pathway in pancreatic cancer, which fuels cancer invasion and metastasis [500].

Elevated l-arginine promotes immune evasion by modulating arginine metabolism in tumor-infiltrating T cells, leading to T cell dysfunction and impaired antitumor immunity [501]. Likewise, stiffness-dependent YAP/TAZ activation and actomyosin tension reprogram amino acid metabolism to sustain anabolic growth, redox balance, and matrix-remodeling functions [502]. Additionally, this metabolic reprogramming reinforces ECM remodeling by providing energy for cytoskeletal contractility and collagen deposition, creating a feed-forward loop where ECM stiffening and metabolic alterations synergize to drive pancreatic tumor progression [503]. Targeting this mechanometabolic axis may therefore represent a dual strategy to inhibit both tumor aggressiveness and immune escape.

#### Immune consequences of stiffness-driven metabolic reprogramming

The immunological impact of this mechanometabolic shift is substantial. Stiffness-associated hypoxia, lactate accumulation, and acidosis suppress the function of effector immune cells [504], including T cells, NK cells, and DCs, while favoring immunosuppressive populations such as Tregs and M2-like macrophages [505]. In this context, ECM stiffness indirectly reinforces immune suppression by establishing the physical and metabolic conditions that maintain an immunosuppressive TME [506]. Overall, ECM stiffening establishes a feed-forward loop in which mechanical stress, metabolic reprogramming, and immune evasion cooperate to promote tumor aggressiveness.

### ECM stiffness and regulation of tumor immunity status

Stiffer ECM creates an environment with reduced T cell infiltration, with significantly reduced infiltrating CD8^+^ T cells, and it was observed in breast cancer cells with high collagen density [192]. Similarly, an increased IL-2 secretion was observed in mouse CD4^+^ T cells grown on a stiffer ECM substrate [507]. Furthermore, ECM stiffness affects macrophage polarization to a greater extent, inducing a proinflammatory, hypo-phagocytic macrophage phenotype via a positive feedback loop involving Piezo1/actin and YAP/TLR4 mechanisms [508]. For instance, the motility and migration of immune cells like macrophages are adversely affected by ECM stiffness, in which they display Rho/ROCK-assisted, podosome-independent amoeboid migration in soft ECM [509]. In contrast, in a stiffer ECM, they adopt a ROCK-independent podosome-assisted macrophage migration [422].

### ECM stiffness and anticancer drug resistance

A rigid ECM forms a diffusion barrier, reducing the efficacy of antitumor drugs and immune checkpoint-based treatments. Trafficking T cells through dense collagen-rich ECM can cause impaired motility and nuclear damage. It was reported that glioma cells grown in stiff hydrogels containing HA exhibited resistance to erlotinib, a U.S. Food and Drug Administration (FDA)-approved epidermal growth factor receptor (EGFR) inhibitor [510]. ECM stiffness reduces cell–cell interactions, compromising barrier integrity and leading to vascular extravasation. This creates a hostile ECM that impairs immune cell infiltration, resulting in immunosuppression and resistance to immunotherapy [104]. ECM rigidity has been shown to modulate ABC transporter function, which in turn affects intracellular drug uptake. A stiffened ECM reduces the activity of ABC transporters, leading to reduced drug efflux from cells [511]. In a study by Baltes et al. [512], breast cancer cells grown on collagen I and FN substrates exhibited significant resistance to doxorubicin, mitoxantrone, and cisplatin via up-regulation of ABC transporter activity. Similarly, breast cancer cells grown on an FN substrate exhibited activation of the FN/PI3K/AKT2/survivin axis, leading to docetaxel resistance [512].

### Signaling pathways related to ECM stiffness and tumor progression

ECM stiffness regulates tumor behavior through a coordinated mechanotransduction cascade that links extracellular mechanical cues to intracellular signaling, transcriptional reprogramming, and further matrix remodeling [229]. At the cell surface, integrins, adhesion molecules, and mechanosensitive ion channels such as Piezo1 act as primary sensors of matrix rigidity [513]. These inputs promote calcium flux, actin polymerization, and myosin-dependent contractility, generating traction forces that are transmitted through FAs and the cytoskeleton to the nucleus via the linker of nucleoskeleton and cytoskeleton complex (LINC). In this way, stiff ECM is converted into biochemical signals that reshape cellular behavior [514].

A central downstream consequence of this mechanical sensing is activation of the integrin-FAK/Src–RhoA/ROCK axis, which functions as a core signal transduction module in stiffness responses. Once integrin-associated forces exceed a critical threshold, proteins such as talin promote FAK activation, followed by Src recruitment and amplification of downstream signaling [515]. This signaling enhances actomyosin tension, cytoskeletal remodeling, and fibroblast contractility while also promoting collagen synthesis in CAFs. Rather than acting as separate parallel pathways, FAK/Src and RhoA/ROCK should be viewed as an integrated mechanosignaling network that couples matrix rigidity to force stromal generation and activation [516].

These mechanical signals converge at the transcriptional level primarily through YAP/TAZ**,** which serve as major nuclear effectors of ECM stiffness [517]. In stiff matrices, cytoskeletal tension and Rho-dependent signaling favor YAP/TAZ nuclear localization and transcriptional activation, thereby promoting programs linked to proliferation [518], stem-like features [519], myofibroblast activation [520], and survival [521]. In CAFs, YAP/TAZ activity further supports profibrotic and pro-angiogenic functions, reinforcing the tumor-promoting stromal state. Other pathways, including TGF-β**,** can cooperate with this mechanotransduction program by sustaining myofibroblast differentiation and matrix deposition, but their relevance here lies in how they strengthen stiffness-dependent stromal remodeling rather than as isolated signaling events. Activation of multiple signaling axis, including IL-33 and insulin-like growth factor receptors like IGF1R, FAK/Src, TGF-β, MAPK, and PI3K/AKT, drives ECM protein synthesis and shapes ECM architecture. Several other pathways, such as the LOX, the procollagen-lys 2-oxoglutarate 5-dioxygenase (PLOD) family, particularly peptidyl-proline cis-trans isomerase (PPIase), and lysyl hydroxylase 2 (LH2), assist in collagen rearrangement and stabilization [56].

The outcome of this signaling hierarchy is ECM remodeling that feeds back to increase tissue stiffness. Mechanotransduction-driven stromal activation enhances deposition, organization, and stabilization of fibrillar ECM components, while collagen-modifying enzymes, such as LOX, PLOD family members, and LH2, promote collagen crosslinking and maturation. This creates a self-reinforcing loop in which stiff ECM activates mechanosignaling, mechanosignaling induces profibrotic transcriptional programs, and these programs further stiffen the matrix. Within this framework, other pathways such as Wnt/β-catenin or PI3K/AKT are best interpreted as context-dependent secondary effectors that intersect with the core stiffness program, rather than as equivalent parallel drivers.

## Clinical and Therapeutic Implications

Understanding ECM remodeling as a driver of immune escape provides a strong rationale for therapeutic strategies that aim to normalize ECM and restore antitumor immunity. The rigid and cross-linked nature of the ECM not only facilitates tumor formation but also hampers the effective distribution of immune cells and anticancer medications within the tumor. Targeting ECM stiffness could be a viable approach to combat cancer, overcome drug resistance, and evade immune surveillance, thereby enhancing the efficacy of cancer treatments [391]. Therapeutic targeting of the tumor-associated ECM can be done in several ways: targeting ECM molecules, targeting enzymes that remodel the ECM, modifying the matrix’s physical or structural characteristics, or controlling fibroblast activities as a covert means to change ECM deposition (Table 1).

**Table 1.** Extracellular matrix components involved in tumor immune escape and therapeutic targeting

ECM component	Function	Immune escape mechanism	Therapeutic strategies
Type I collagen	~90% of total body collagen; abundant in skin, bone, tendon, ligament, and many organs	• COL I forms a barrier to T cell, increases stiffness, vessel compression, and supports immune exclusion via DDR1, and LAIR-1-collagen signaling [591]	• LOX/LOXL inhibition to reduce crosslinking • DDR1 blockade [626] • LAIR-1/collagen blockade [650], collagenase and stromal-remodeling in combination with immunotherapy [627]
Type II collagen	Supports joints and cartilage resilience	• Direct evidence for a specific immune-evasion role in solid tumors is limited [651]	• No established tumor-specific COL II-targeted immune therapy [651]
Type III collagen	Supports extensible tissues and regulates fibrillar matrix architecture	• COL III reflects CAF-driven fibrosis, corelate with immune exclusion and poor drug penetration [652]	• Stromal normalization, antifibrotic strategies [653] • Biomarker use of COL III fragments/pro-peptides to stratify fibrotic tumors rather than direct COL III inhibition alone [654]
Type IV collagen	Core structural scaffold of BM, regulates polarity, filtration, and tissue compartmentalization	• BM remodeling exposes bioactive fragments and facilitates invasion [655] • Altered COL IV-rich BM can support tumor progression and immune dysregulation [104]	• Target BM remodeling, collagen-receptor signaling, and collagen-immune checkpoints [587] • Indirect targeting via LAIR-1 axis or ECM-remodeling therapy [587]
Type V collage	Controls collagen fibril assembly and diameter	• Indirectly contribute to shaping fibril organization and matrix architecture, thereby influencing stiffness and immune cell trafficking [656] • Direct immune-escape evidence is still limited [656]	• No established direct therapy; potential indirect relevance through fibrillogenesis/stromal remodeling
Type VI collagen	Organizes pericellular matrix, cell adhesion, and matrix-cell interactions.	• COL VI and its fragment endotrophin promote fibrosis [657], tumor progression, inflammation, macrophage skewing, and an immunosuppressive microenvironment • Elevated COL VI/COL6A3 is associated with poor outcomes in multiple cancers [658]	• Target COL VI/COL6A3-endotrophin axis [659], stromal fibrosis, macrophage polarization, and combinatorial immunotherapy in fibrotic tumors [657]
Type VII collagen	Anchors epithelium to underlying stroma	• COL VII loss or deficiency can alter TGF-β and epithelial microenvironments, especially in cutaneous SCC settings [660] • Not yet a broadly established pan-cancer immune-escape collagen [660]	• Context-specific replacement/restoration concepts in COL VII-deficient disease; not an established general immuno-oncology target [661]
Type X collagen	Critical in endochondral ossification	• In cancer, COL10A1 is more often considered a stromal/tumor progression biomarker than a defined immune-evasion effector [662] • May reflect matrix remodeling and aggressive behavior	• Biomarker-guided stratification; no established direct collagen-X-targeted therapy
Types IX, XII, XIV, XIX, XXI (FACIT collagens)	Modulate fibril organization, spacing, interfibrillar interactions, and matrix architecture	• FACIT collagens indirectly shape immune escape by reorganizing fibrillar networks [8] • Among them, COL XII is the strongest cancer-linked example [663] • CAF-derived COL XII reorganizes COL I, promotes pro-invasive matrix architecture, and is associated with metastasis and poor survival [189]. • Evidence for IX/XIV/XIX/XXI in immune escape is much more limited and mostly emerging [664]	• For COL XII: stromal targeting, CAF modulation, collagen-organization interference, and use as circulating biomarker [663]. • Other FACIT collagens currently lack established direct therapeutic programs
Elastin	Composed of tropoelastin subunits crosslinked with fibrillin microfibrils that form the elastin fibers. The *ELN* gene codes for the human tropoelastin	• Critical role in ECM stiffening by elastin/collagen crosslinking mediated by LOX enzymes [665]) • Positively correlates with fibroblast molecular marker VIM to promote EMT [640] • Elastin degradation produces EDPs, which induce tumorigenesis and immune escape [642]	• Targeting EDP/Elastokines, α_v_β_3_ and α_v_β_5_ integrins [643] • Targeting Elastokines/ERC binding, facilitating Ca^2+^ channel opening and activating FAK, c-Src, PDGF, and Ras-Raf-MEK1/2-ERK1/2 pathways [644]
Fibronectin	Arranged into a mesh of fibrils and linked to integrins for signal transduction, targeting cell adhesion, growth, migration, and differentiation. Two forms of fibrinogen, plasma fibrinogen and cellular fibrinogen, are secreted by fibroblast T cells.	• TGF-β-dependent immune escape via expansion of the tolerogenic Treg compartment [666] • Mobilize active TGF-β1 through αvβ8 integrins to evade the immune system [645] • Leukocyte extravasation and immune escape via integrin αvβ3/PI3K/AKT/SOX2 signaling pathway [667]	• ILT3-specific antibody targeting FN [668] • Tumor-specific FN splice variant EDA with CAR-T cells [198,669], Human FN-EDB [670]
Laminins	A major structural component of the BM is a glycoprotein. It is a family of trimetric proteins containing α, β, and γ subunits. Critical role in angiogenesis, FN-generating fibrogenic fibroblast activation, immune cell infiltration, and immune evasion	• Laminin γ2 mediate T cell exclusion via TGF-β1 mediated Laminin γ2/JNK/AP1 signaling [671] • Laminin 322 maintains stem cell state and chemoresistance via mTOR signaling [672]	• Administration of the TGF-β inhibitor galunisertib inhibits laminin γ2 to favors T cell infiltration into the tumor niche [673] • Inhibiting 67LR binding to laminin could block tumor aggressiveness [285] • The laminin-332 receptor is a prognostic marker used to check chemoresistance [674]
Tenascin	ECM glycoproteins are modulators of cell adhesion, migration, and differentiation. Bind to VEGF to stimulate cell proliferation via integrin.	• The CD47–SIRPα axis inhibits the phagocytosis of tumor cells by macrophages and other myeloid cells [675] • TN-C inhibits the proliferation of TIL [676] • Arrest T cell activation in prostate cancer [677]	• CD47 knockout showed significant phagocytosis in glioblastoma cells [678] • Vaccination using tumor-derived peptides triggers specific immune responses in glioblastoma [679] • Centyrin and Tencon molecules employ TNC protein sequences [680]
Nidogen/entactin	It is a sulfated glycoprotein in the BM that binds with laminins, PGs, hyaluronan, and collagens to form ternary complexes.	• NID2 is associated with a poor response to immunotherapy in melanoma [681] • NID1 is associated with immune surveillance in glioma [682]	• NID2 is a therapeutic target of fibroblast activation [682] • NID1 is a potential prognostic biomarker of glioblastoma [683]
Matrix metalloproteases (MMPs)	Proteolytic calcium-dependent endopeptidases that degrade ECM. Create a suitable environment that supports the initiation of a primary tumor along with assisted neovascularization. Facilitates the penetration of connective tissue architecture and assists in neovascularization.	• Key mediator of ECM degradation. • MMP-1, -2, and -9 down-regulate the interleukin receptor on the T cell surface [684] • MMP-7 resists FasL-triggered apoptosis in CRC cells [685] • Modify cell-surface cytokine receptors and adhesion molecules [686] e initiation of a primary tumor along with assisted neovascularization [687] • In melanoma, MMP-2-holding DCs prime CD4^+^ T cells to differentiate into the Th2 pathway, allowing cancer cells to escape [290]	• Targeting MMP-9 promotes an antitumor immune response by inducing neutrophil infiltration and activating tumor-infiltrating macrophages [688] • Target PAR-2-mediated MMP-2 expression that promotes immune escape from T cells [689] • Arrest specific proteins of p38 MAPK/MK2/HSP27 axis [690] • MMP-13 knockdown by treatment with MMP-13 antisense oligonucleotide [691] • IGFBP-1 regulation can directly influence MMP-9-mediated tumor evasion [689]
Proteoglycans and GAGs	Proteoglycans partner with various ECM components to facilitate chemokine signaling, invasion, and tumor metastasis. Variations in sulfation patterns in GAGs serve as biomarkers for different types of cancer. GAGs form part of the proteoglycan complex except HA. These hydrating molecules can orchestrate the tumor’s immune evasion strategy by altering the biophysical properties of the tumor.	• HA barrier inhibits access to NK cells, preventing them from executing cytotoxic effects on tumor cells [692] • HS side chains of syndecans and glypicans bind to VEGF, EGF, PDGF, and FGF to promote tumorigenesis and immune escape by interacting with NK Cells, DCs [693]	• WT1 peptide vaccine for glioblastoma [694] • GPC3 peptide vaccines for hepatocarcinoma [695] • Chondroitin sulfate proteoglycan 4 (CSPG4) as a target antigen for glioblastoma [696] • Target Wnt/Frizzled/β-catenin pathway [697] • Target MMP-7/syndecan-1/TGF-β autocrine loop [698]
Perlecan (HSPG2)	Part of BM. Controls the cell-surface receptors and growth factors by forming mitogenic and morphogenic variants. Interact with ECM proteins and growth factors during the processes of adhesion and migration.	• Facilitate ECM remodeling and immune evasion [699]	• Anti-HSPG2 antibodies for triple-negative breast cancer [700] • Antisense targeting of perlecan reduces tumor growth, antiperlecan/LG3 antibody [681]
Agrin	Glycosylated proteoglycan protein. Involved in cell cycle, cell proliferation, invasion, and migration. Regulate ECM-cancer cell communications and promote oncogenic signaling	• Influence on NSCLC growth and tumor-infiltrating Treg cells, making a negative correlation with immunotherapy [701] • Increased IL-6 expression through the PI3K/AKT pathway [701] • Agrin enhances tumorigenesis by activating FAK and MAP kinase signaling pathways [702] • MA07.11 Agrin-positive tumor cells suppress cancer immunotherapy in lung adenocarcinoma [703]	• Agrin knockdown suppresses Tregs and IL-6 secretion [704] • Lrp4/MUSK acts as a potent therapeutic target [705] • Agrin acts as a prognostic biomarker in hepatocellular carcinoma [706]
Fibulin	ECM glycoproteins are associated with BM, elastic fibers, FN microfibrils, and proteoglycan aggregates. Act as intermolecular bridges within the ECM and as mediators for ECM remodeling and cell signaling for proliferation and differentiation.	• Activates pro-invasive NF-κB signaling in glioblastoma [707] • Fibulin- activates ADAM-17 by competing with TIMP3, with the release of soluble TNF-α and activating canonical NF-κB signaling [708] • Fibulin-5 blocks TRPV1 to activate ROS/MAPK and AKT pathways [709] • Estradiol and Fibulin-1 inhibit FN-induced motility of tumor cells [710]	• Inhibition of fibulin-3 suppresses ADAM17/Notch/NF-κB pathway activation [708] • Block Fibulin-5 to prevent metastasis [711]
Lumican	Structure regulatory proteoglycan of the ECM with cell instructive property by interacting with the receptors in cell proliferation, differentiation, inflammation, and innate/humoral immune responses	• CAFs-derived Lumican induces tumor progression via immune evasion through the β1-FAK pathway in Gastric cancer [712]	• Target the AC117386.2/hsa-miR-378c/LUM regulatory axis [713] • Lumican targets the miR200 family for epithelial–mesenchymal transition [714]
Periostin	The matricellular protein of the ECM acts as a ligand for β3 and β5 integrins, supporting the adhesion and migration of endothelial cells. Highly expressed in breast cancer cells [715]	• Cancer stem cell maintenance by establishing a protumorigenic niche. • Interaction of fibroblast secreted periostin and tumor-derived IL-6 promotes colitis-associated Colorectal cancer [716]	• Twist shRNA can inhibit Periostin expression in lung cancer [717] • Periostin knockout can regulate collagen crosslinking [717]
Growth factors	Growth factors FGF, PDGF, VEGF, EGF, and TGF-β1 interact with the ECM to ensure that cells maintain appropriate proliferation, growth, division, differentiation, and apoptosis.	• TGF-β1 promotes cancer immune evasion by facilitating the trans differentiation of NK cells into ILC1, which lacks cytotoxic function [718] • API5 acts as an immune escape gene by up-regulating FGF2 signaling through an FGFR1/PKCδ/ERK effector pathway and degrading proapoptotic molecule BIM [719]	• Target AP15 immune escape gene and FGFR1/PKCδ/ERK pathway [701] • Monoclonal antibodies like trastuzumab and rituximab block growth signals [720]
ADAMs	ADAMS contains adhesive and metalloprotease domains that can remodel ECM components. These domains are key modulators of cell–cell and cell–matrix interactions.	• ADAM-17 present in platelets is involved in tumor immune evasion. ADAM-17 mediated cleavage of TGF-α promotes tumor metastasis [721] Regulates PD-L1 and PD-1 interaction in breast cancer cells [722] • Hypoxia induces up-regulation of ADAM-10, causing decreased NKG2D expression in NK cells and facilitating tumor escape [707]	• D8P1C1, an anti-ADAM 17 monoclonal antibody, can resist tumor growth in triple-negative breast cancer [723]

ADAM, a disintegrin and metalloproteinase; ADAM10, a disintegrin and metalloproteinase domain-containing protein 10; ADAM17, a disintegrin and metalloproteinase domain-containing protein 17; AKT, protein kinase B; AP1, activator protein 1; API5, apoptosis inhibitor 5; BIM, BCL-2-like protein 11; BM, basement membrane; CAF, cancer-associated fibroblast; CAR-T, chimeric antigen receptor T cell; CD4, cluster of differentiation 4; COL, collagen; COL 1, collagen type 1; COL10A1, collagen type 10 α1 chain; COL6A3, collagen type 6 α3 chain; COL II, collagen type 2; COL III, collagen type 3; COL IV, collagen type 4; COL VI, collagen type 5; COL VII, collagen type 7; COL XII, collagen type XII; CRC, colorectal cancer; CSPG4, chondroitin sulfate proteoglycan 4; c-SRC, cellular SRC proto-oncogene tyrosine-protein kinase; DCs, dendritic cells; DDR1, discoidin domain receptor 1; D8P1C1, anti-ADAM17 monoclonal antibody candidate; ECM, extracellular matrix; EDA, extra domain A; EDB, extra domain B; EDP, elastin-derived peptide; EGF, epidermal growth factor; ELN, elastin; ERK 1/2, extracellular signal-regulated kinase 1/2; ERC, elastin receptor complex; FACIT, fibril-associated collagens with interrupted triple helices; FAK, focal adhesion kinase; FasL, Fas ligand; Fc, fragment crystallizable region; FGF, fibroblast growth factor; FGF2, fibroblast growth factor 2; FGFR1, fibroblast growth factor receptor 1; FN, fibronectin; GAGs, glycosaminoglycans; GPC3, glypican 3; HA, hyaluronan; HSP27, heat shock protein 27; HSPG2, heparan sulfate proteoglycan 2; HS, heparan sulfate; IGFBP-1, insulin-like growth factor-binding protein 1; IL-6, interleukin-6; ILC1, group 1 innate lymphoid cell; ILT3, immunoglobulin-like transcript 3; JNK, c-Jun N-terminal kinase; LAIR-1, leukocyte-associated immunoglobulin-like receptor 1; LOX, lysyl oxidase; LOXL, lysyl oxidase-like; LRP4, low-density lipoprotein receptor-related protein 4; LUM, lumican; MAPK, mitogen-activated protein kinase; MEK 1/2, mitogen-activated protein kinase kinase 1/2; miR-378c, microRNA-378c; MK2, MAPK-activated protein kinase 2; MMP-1, matrix metalloproteinase 1; MMP-2, matrix metalloproteinase 2; MMP-7, matrix metalloproteinase 7; MMP-9, matrix metalloproteinase 9; MMP-13, matrix metalloproteinase 13; MUSK, muscle-associated receptor tyrosine kinase; NF-κB, nuclear factor κB; NID1, nidogen 1; NID2, nidogen 2; NK, natural killer; NKG2D, natural killer group 2D; NSCLC, non-small cell lung cancer; PAR-2, protease-activated receptor 2; PD-1, programmed cell death protein 1; PDGF, platelet-derived growth factor; PD-L1, programmed death-ligand 1; PG(s), proteoglycan(s); PI3K, phosphoinositide 3-kinase; PKCδ, protein kinase Cδ; p38 MAPK, p38 mitogen-activated protein kinase; Raf, rapidly accelerated fibrosarcoma kinase; Ras, rat sarcoma viral oncogene homolog; ROS, reactive oxygen species; SCC, squamous cell carcinoma; shRNA, short hairpin RNA; SIRPα, signal regulatory protein α; SOX2, SRY-box transcription factor 2; TGF-α, transforming growth factor α; TGF-β1, transforming growth factor β1; Th, T helper; TIL, tumor-infiltrating lymphocyte; TIMP3, tissue inhibitor of metalloproteinases 3; TNF-α, tumor necrosis factor α; TN-C, tenascin-C; Treg, regulatory T cell; TRPV1, transient receptor potential vanilloid 1; VEGF, vascular endothelial growth factor; VIM, vimentin; Wnt, wingless-related integration site; WT1, Wilms tumor 1; 67LR, 67-kDa laminin receptor

ICI-based therapies, which target regulatory pathways such as PD-1/PD-L1 and CTLA-4 to enhance T cell-mediated antitumor immunity, have demonstrated significant clinical efficacy in several cancers, including small cell lung carcinoma and melanoma [522]. Their efficacy in glioblastoma remains limited because of the highly complex central nervous system (CNS) TME. Moreover, glioblastoma is considered an immunologically “cold” tumor, characterized by low infiltration of cytotoxic T cells and a paucity of other effector immune cells [523]. Furthermore, the blood–brain barrier restricts the entry of both immune cells and therapeutic antibodies into the brain. In addition, glioblastoma generally has a low tumor mutational burden, leading to reduced neoantigen availability, while concurrently secreting high levels of immunosuppressive factors, including TGF-β, IL-10, and IDO [524].

Given that the elements of the ECM can function as endogenous mediators of inflammation linked to cancer, this presents a unique concept that targeting these mediators may alleviate immunosuppression [525]. Fibrillar collagen, a major factor in the increased stiffness of the ECM, has emerged as a potential target in cancer therapeutics [526]. Direct depletion of collagen by recombinant collagenase has emerged as a possible treatment strategy for cancer [527]. Prior research has shown that certain regulators of ECM stiffness, such as mechanosensors and mechanotransducers, can be targeted in combination with drugs [528]. Yet, specific collagen-depleting therapies have failed, underscoring the need for caution when targeting CAFs in cancers like PDAC. Reports suggested that the depletion of α-SMA-positive CAFs led to invasive, undifferentiated tumors with enhanced hypoxia, EMT, and cancer stemness in mouse models, with no responsiveness to gemcitabine, whereas anti-CTLA-4 immunotherapy significantly reversed disease progression [527]. Similarly, the depletion of the *COLLA1* gene, which encodes the α1 subunit of collagen type I, in α-SMA-positive CAFs has been shown to remarkably reduce stromal collagen type 1, concomitant with the suppression of CD8^+^ T cells, subsequently leading to the progression of pancreatic cancer with a reduced survival rate [529]. Similarly, the off-target destruction by ECM-degrading factors could prove detrimental to the body, leading to excessive bleeding events, as observed with the use of α2bβ3 antagonists, which alter platelet function. Therefore, systemic administration of such treatments to achieve target-directed recognition is necessary in diseases such as cancer [529]. To avoid such effects, a proper 3D model for each tumor type TME is needed, elucidating the cell–cell and cell–ECM connections and organizational properties.

For the same reason, the ECM properties of each cancer type should be precisely determined, thereby drastically reducing the rate of false-positive results. Similarly, the off-target action of collagenase and hyaluronidase enzymes carries the risk of promoting tumor metastasis by disrupting physical barriers and releasing pro-invasive fragments. To overcome such adverse effects, researchers are leveraging endogenous ECM components, such as FN and laminin, to conjugate with ECM-mimicking nanoparticles without triggering massive pathological ECM remodeling. This targeted approach reduces the risk of metastasis by enhancing the accumulation of ECM-degrading moieties within the tumor.

### Targeting ECM stiffness for improving immunotherapy

The rigid, interconnected ECM facilitates tumorigenesis and hinders the penetration of immune cells and anticancer drugs into tumors. This stiffness will eventually create an immunosuppressive environment by acting as a physical impediment to immune cell infiltration, like T cells, NK cells, and macrophages [88]. Therefore, reducing ECM stiffness could be a viable approach in cancer treatment to combat drug/immune resistance. Several immunotherapeutic strategies targeting ICIs [530], oncolytic virotherapy [531], DC vaccines [532], cytokine-mediated therapies [533], CAR-T therapy [534], and TAM-mediated therapies [535] are at the forefront among the modern cancer treatments, yet the primary factor to target should be the stiffness and related topology of the tumor ECM.

#### Combining ECM modulation with ICB

ICB therapy blocks immune-checkpoint proteins to boost antitumor responses. Still, its efficacy is adversely affected by the stiffened ECM in tumors, which hinders immune cell infiltration [536]. Elevated ECM stiffness can compress tumor vasculature, leading to poor perfusion and, in turn, tissue hypoxia, which transcriptionally up-regulates PD-L1 under the influence of HIF-1α, a key transcriptional regulator in hypoxic conditions that helps cancer cells evade the immune system [537]. Therefore, a combinational therapy targeting HIF-1α with anti-PD-L1 antibodies blocks hypoxia-driven signaling responses and reduces immune evasion [538]. A similar approach was demonstrated in a study by Ding et al. [539], in which PD-L1 expression was abrogated by either HIF-1α knockdown or HIF-1α inhibitor treatment in glioma. Similarly, another hypoxia-responsive factor, LncRNA MIR155HG, has been reported to promote PD-L1 expression in HCC, and knocking down this LncRNA substantially reduces the expression of HIF-1α and PD-L1 within tumors, thereby enhancing immune cell infiltration [540]. Therefore, PD-1/PD-L1 machinery is closely linked with ICB-based therapies. Additionally, improving PD-1 blockade therapies by targeting collagen crosslinking degradation could drastically enhance T cell migration [541]. Lysyl oxidase-like 4 (LOXL4) inhibitors also represent an active target for ICB therapies, as exposure of macrophages to LOXL4 can elevate PD-L1 expression, thereby fostering an immunosuppressive phenotype [542]. Another promising approach is anti-CTLA-4 therapy, which accelerates the survival outcomes in patients with metastatic melanoma in a noninvasive manner [543]. CTLA-4 binds to CD80/CD86 ligands on CD28, leading to subsequent interactions with T cell receptors. Additionally, granzyme B and other proteases, such as MMP-2/9, assist in the degradation of type III and IV collagens, thereby alleviating the process [544]. These processes also effectively slow down PD-L1 expression, which altogether enhances CTLA-4 or PD-1-based ICB therapies [361].

#### Targeting ECM stiffness to improve ACT therapy

Adoptive cell therapy (ACT) is a personalized immunotherapeutic strategy that employs genetically engineered T cells expressing either CARs or modified T cell receptors (TCRs). However, CAR-T cells frequently display limited trafficking to target sites and undergo metabolic reprogramming that promotes functional exhaustion [545]. FN-targeted T cell therapies typically yield remarkable outcomes, demonstrating efficacy in advanced stages of cancer. A phase I clinical trial conducted in 2014 using FN-CH296 showed quite promising results [546]. Nattokinase-like drugs aid in reducing tumor ECM stiffness by degrading FN, thereby combating fibrosis enhanced by CAFs. This further augment the therapeutic efficacy of CAR-T therapy [547]. Another emerging strategy involves targeting laminin within the tumor ECM, which has been shown to enhance immune cell infiltration into the TME by promoting ECM degradation [548]. Targeting FAP in the tumor stroma with chimeric CAR-T showed promising results with minimal toxic effects [549]. Again, it will substantially diminish further FAP-expressing stromal cells, leading to a substantial reduction in tumor size. Nevertheless, combining anti-FAP–CAR-T approaches with PD-1 inhibitors may potentially improve anticancer efficacy. Gulati et al. [550] developed anti-FAP–Δ-CD28/CD3ζ CAR-T cells and reported superior in vitro functionality, better tumor control when combined with PD-1 blockade in humanized mice, and persistence for up to 21 d in a patient with malignant pleural mesothelioma. At present, CAR-T therapy follows a personalized treatment paradigm in which autologous T cells are collected from the patient, genetically modified, expanded ex vivo, and reinfused [551]. The broader application of allogeneic CAR-T remains constrained by the risk of graft-versus-host disease (GvHD). Because GvHD is largely driven by alloreactive αβ-TCRs, multiple studies have explored gene-editing strategies to eliminate endogenous TCR expression in CAR-T [552]. However, concerns regarding off-target effects and unintended genotoxicity associated with these editing approaches have prompted continued efforts to identify alternative CAR-T platforms with inherently restricted or absent TCR repertoires [553]. This includes γδT cells, iNKT cells, virus-specific T cells, double-negative T cells (DNT cells), and MAIT cells [554,555]. γδT cells are a unique subset of T lymphocytes that do not require MHC antigen presentation, which is advantageous in the sense that many conventional T cell-based immunotherapies failed due to the ability of tumor cells to down-regulate MHC molecules for immune escape mechanisms [556]. DNT cells are a rare T cell subset that lack both CD4 and CD8 co-receptors. A recent study by Tin et al. [557] showed that DNT cells utilize TNF-α–JAK1–ICAM-1 cytotoxic axis to mediate cytotoxicity against AML. Furthermore, allogeneic DNTs do not induce GvHD, which makes them a suitable candidate for immunotherapy when transduced with CAR. Additionally, CAR-T patients with long-term remission have been reported to exhibit a DNT phenotype [558]. In parallel, cytotoxic immune populations such as NK cells and cytokine-induced killer (CIK) cells, together with nonlymphoid effectors including neutrophils and macrophages, as well as stem/progenitor cells and cell-free extracellular vesicles, are increasingly being explored as next-generation therapeutic platforms [559]. A recent study by Lonez et al. [560] suggested the possibility of a non-gene editing technology known as CYAD-211, which constructed a fully functional allogeneic anti-B cell maturation antigen CAR-T that expresses an anti-CD3ζ miRNA-based short hairpin RNA (shRNA) within the CAR construct. CYAD-211 was reported to effectively inhibit TCR-mediated signaling cascades in vitro and suppress GvHD in vivo, which subsequently supported its advancement into a phase I clinical trial in patients with multiple myeloma [560].

#### Targeting ECM stiffness to improve oncolytic virus-mediated therapy

Oncolytic viruses (OVs) selectively replicate within malignant cells, leading to tumor-cell destruction and the release of immunostimulatory signals that subsequently engage and amplify host antitumor immune responses. Once they reach the host, OVs attach to the cell receptors and replicate using the host replication machinery [561]. Since ECM already imposes a barrier to OVs, the rapid generation of neutralizing antibodies and robust host immune responses may pose substantial safety concerns, particularly in immunocompromised patients, thereby limiting the broader clinical applicability of this therapeutic approach [562]. Currently employed oncoviral therapy targets HA, relaxin, and core protein polysaccharides. The oncolytic adenovirus ICOVIR17 promotes HA degradation in stiff ECM and accelerates PD-L1 expression in macrophages and cancer cells, assisting the infiltration of CD8^+^ T cells and macrophages into the tumor [563]. Another specially engineered OV, VCN-01, produces the hyaluronidase enzyme that cleaves the tumor stroma, facilitating chemotherapy [564]. Similarly, relaxin expression from a tumor-targeting adenovirus has been reported, with higher transduction efficiency and viral spread throughout the tumor mass, as shown in a study in the B16BL6 melanoma mouse model [565]. Additionally, core protein polysaccharides, such as decorin, expressed by adenoviruses are well established for degrading the stiff ECM by reducing collagen fiber diameter, modulating TGF-β signaling, and enhancing MMP-1 activity [566].

#### Targeting ECM to improve TCVs

Therapeutic cancer vaccines (TCVs) are a type of cancer immunotherapy that activate and expand antigen-specific DCs and CD8^+^ and CD4^+^ T cells and are specifically designed to stimulate the patient’s immune system to target and destroy cancer cells [567]. Most trials of this treatment strategy are still ongoing, with early investigations of mRNA, DNA, or peptide-targeting therapies in solid tumors [568]. Thus, within the context of ECM-directed therapy, the most relevant vaccine strategies are those that couple immune activation with stromal reprogramming, thereby improving T cell access to tumor nests.

Among these, targeting FAP-expressing CAFs represents a particularly compelling approach. FAP^+^ CAFs are central mediators of desmoplasia, collagen deposition, matrix stiffening, and immune exclusion, and therefore constitute an indirect but functionally important ECM-associated target [569]. In murine models of desmoplastic tumors, an orally delivered DNA vaccine targeting FAP reduced intratumoral collagen I deposition and increased doxorubicin uptake, thereby enhancing the efficacy of combination chemotherapy [570]. Similarly, vaccination against the extra domain B (EDB) of FN, an oncofetal ECM splice variant enriched in tumor neovasculature and tumor stroma, could break tolerance, induce anti-EDB antibodies, and reduce tumor growth in mice [571]. This study supports the principle that tumor-associated ECM antigens can themselves serve as vaccine targets [572]. Such findings position ECM-targeted vaccination not only as a strategy for antigen-specific immune activation but also as a means of converting an immune-excluded, matrix-dense TME into one that is more permissive to immune cell infiltration and therapeutic delivery [572].

Vaccine platforms directed against tumor-associated antigens such as human telomerase reverse transcriptase (hTERT), as well as broader mRNA- or adjuvant-based cancer vaccines, remain important in cancer immunotherapy [573]. The relevance of hTERT-targeted vaccines lies not in direct matrix degradation, but in their ability to generate sustained tumor-reactive T cell responses that may become more effective when combined with strategies that relieve ECM-mediated immune exclusion [574]. Nevertheless, in the specific context of ECM-targeted vaccination, their relevance would be greatest when they are linked to measurable effects on CAF activity, collagen remodeling, matrix composition, or stromal architecture [575]. Similarly, adjuvanted vaccine platforms that enhance DC activation and T cell priming, including those engaging inflammasome signaling, may help overcome the immunosuppressive stromal milieu, but their contribution to ECM modulation remains largely indirect unless accompanied by measurable effects on CAF activity, collagen organization, or matrix stiffness [196]. The OFA-iLRP vaccine is somewhat more relevant to this context because laminin receptors are linked to matrix-associated signaling and tumor–stroma interactions; thus, targeting such antigens may influence how immune responses are deployed within laminin-rich tumor niches [576]. More broadly, mRNA-based cancer vaccines provide a flexible platform that can be adapted not only to encode tumor-associated antigens but also, in principle, stromal or ECM-related targets, thereby enabling combined enhancement of immune priming and microenvironmental reprogramming [577]. Therefore, within an ECM-focused framework, these vaccine approaches should be interpreted primarily as immune-activating platforms whose therapeutic value may be amplified when they are used to counteract ECM-driven immune exclusion or are combined with stromal-targeting interventions. By reducing stromal barriers, loosening collagen-dense matrices, and mitigating CAF-driven immune exclusion, ECM-targeted vaccines may enhance the recruitment, infiltration, and function of effector T cells within tumors [578]. Future vaccine design will likely benefit from combining conventional tumor-antigen targeting with stromal or matrix-directed interventions, particularly in tumors where the ECM is a dominant determinant of immune failure and therapeutic resistance.

#### ECM degradation using nanocarriers

In addition to existing approaches, nanocarriers have shown promise in improving immunotherapy outcomes. In this context, the most relevant systems are not simply those that deliver immunostimulatory agents, but those that either degrade matrix components, respond to ECM-associated cues, or exploit ECM-binding elements to improve immune cell access and therapeutic penetration. One major strategy involves nanoplatforms that directly promote ECM degradation to relieve matrix stiffness and improve intratumoral transport. MMP-incorporated nanovaccines exemplify this concept, as they are designed to degrade dense stromal barriers in a controlled manner, thereby facilitating payload release, improving tissue penetration, and enhancing downstream photodynamic immunotherapy [579]. Similarly, HA-based nanotherapeutics target another key structural determinant of the desmoplastic matrix. By remodeling HA-rich tumor stroma, these systems can reduce matrix compactness, improve intratumoral diffusion, and create conditions that favor the infiltration of CD4^+^ and CD8^+^ T cells. In a murine melanoma model, an HA-based nanovaccine carrying TRP2 and gp100 was evaluated in both prophylactic and therapeutic settings, supporting the concept that matrix remodeling can be integrated with antigen-specific immunization to improve antitumor immunity [580]. A second group comprises ECM-responsive or stromal-normalizing nanocarriers, in which the matrix-related contribution is indirect but still mechanistically relevant [581]. For example, biomimetic gelatin-based nanotherapeutics have been used to silence CD73 and modulate tumor hypoxia, thereby improving responsiveness to PD-1/PD-L1 blockade [582]. Although this approach is not a direct ECM-degrading strategy in the strict sense, its use of a gelatin-based, matrix-relevant platform and its capacity to mitigate hypoxia-related stromal immunosuppression place it within a broader stromal-reprogramming framework [78]. Such systems are best interpreted as nanocarriers that function within the ECM-rich microenvironment to normalize conditions that otherwise reinforce immune exclusion.

A third and more selective category includes ECM-associated antigen-delivery platforms. Nano-emulsion vaccines loaded with laminin peptides do not directly degrade the matrix but instead target matrix-associated antigens and may influence immune recognition within laminin-rich tumor niches [583]. Yang et al. [582] developed an intranasal self-assembled nanoemulsion nanovaccine carrying IKVAV, a laminin-derived peptide, together with the OVA epitope. The platform enhanced DC uptake and induced stronger CD8^+^ T cell and Th1 responses**,** making it an excellent ECM-associated antigen-delivery platform rather than a matrix-degrading system. Similarly, Tsoras and Champion [584] reported cross-linked peptide nanoclusters carrying epitopes from an oncofetal laminin receptor protein as a cancer peptide vaccine delivery system. This is slightly different from targeting a structural ECM protein itself, but it still fits the broader idea of matrix-associated antigen delivery because the target is closely linked to laminin biology and tumor-associated matrix interactions. Overall, nanocarrier-based strategies are most compelling in the ECM setting when they combine matrix remodeling, stromal normalization, or ECM-responsive delivery with immune activation.

## Challenges and Opportunities in Developing ECM-Targeted Therapies

ECM stiffening is a major barrier to effective cancer immunotherapy because it promotes both physical and biochemical immune evasion. Mechanistically, a stiff ECM enhances integrin-mediated activation of TGF-β signaling, which contributes to immune suppression [391]. Increased matrix stiffness and collagen density are also associated with elevated PD-L1 expression in cancer cells and reduced infiltration and function of cytotoxic T cells within tumors [192]. In addition, mechanosensitive pathways such as Piezo1 may further reinforce immunosuppression by promoting macrophage polarization toward tumor-supportive phenotypes, thereby limiting cytotoxic T cell abundance and proliferation.

These observations provide a strong rationale for therapeutic strategies aimed at reducing ECM stiffness and improving drug penetration. One major approach is the use of ECM-degrading agents, including collagenase, relaxin, and hyaluronidase, to disrupt the dense tumor matrix and facilitate delivery of anticancer therapies [585]. However, this strategy also presents important challenges. Excessive or nonspecific matrix degradation may damage normal tissues, trigger immunogenic or toxic effects, and, in some contexts, generate collagen degradation products that promote angiogenesis and metastasis [586]. Therefore, the safety, specificity, and biological consequences of ECM-degrading therapies must be carefully evaluated before clinical translation.

Despite these limitations, several preclinical studies support the therapeutic potential of controlled ECM remodeling. In vivo studies have shown that collagenase treatment can substantially enhance the diffusion and uptake of drug and gene delivery systems in solid tumors, including osteosarcoma [587] and colorectal carcinomatosis [588]. More recently, advances in nanotechnology and biomaterial engineering have enabled more localized and controlled ECM-targeting strategies. For example, thermosensitive hydrogel systems have been used to co-deliver collagenase with trastuzumab, improving intratumoral penetration of antibody therapy in HER2-positive tumor models while limiting off-target exposure [589].

Additional innovative approaches include biologically engineered platforms that remodel the tumor matrix while simultaneously enhancing antitumor immunity. Genetically engineered *Salmonella typhimurium* capable of degrading collagen I has been shown to remodel the ECM in PDAC, reduce intratumoral immunosuppressive cell populations, and improve the efficacy of combination immunotherapy [590]. Similarly, OVs have emerged as multifunctional platforms for ECM-targeted therapy. Modified oncolytic adenoviruses expressing relaxin [591], MMP-8 [592], or decorin [593] have been developed to remodel the TME and enhance immune-mediated tumor clearance. In a xenograft model of human renal carcinoma, a decorin-armed OV combined with carbonic anhydrase IX-targeted CAR-T therapy improved T cell persistence and therapeutic efficacy [593]. Some OVs have also been engineered to express bispecific T cell engagers (BiTEs) [594], enabling simultaneous targeting of stromal components and tumor cells. Table 2 details the major ECM components and their clinical translational status.

**Table 2.** Clinical and translational development status of ECM-targeted therapeutic strategies

ECM components	Clinical or translational development status
Type I collagen	• NC410, a LAIR-2 Fc fusion protein designed to block LAIR-1-collagen inhibitory signaling, has shown preclinical immune-activating activity and has entered phase Ib/II clinical testing in combination with pembrolizumab [724] • DDR1-directed therapeutic strategies remain largely preclinical or early translational [725]
Type II collagen	• No established direct oncology program; currently exploratory only[726]
Type III collagen	• Mainly at the biomarker/translational development stage; not yet a mature direct therapeutic drug class [727] PRO-C3 released during type III collagen formation is a circulating biomarker of ECM remodeling and fibrosis [728]
Type IV collagen	• Mostly preclinical and biomarker-oriented [729]
Type V collagen	• Emerging preclinical therapeutic target [730]
Type VI collagen/COL6A3/endotrophin	• Largely preclinical, with stronger biomarker potential (endotrophin) than direct clinical therapeutic development [731]
Type VII collagen	• Preclinical or disease-specific exploratory stage [732]
Type X collagen/COL10A1	• Diagnostic or prognostic biomarker and a preclinical therapeutic target with no established clinical therapy [733]
FACIT collagens, especially COL XII	• COL XII is at the biomarker/preclinical-translational stage [663] • Other FACIT collagens remain early exploratory targets [610]
Elastin/elastokines	• αvβ3/αvβ5 integrin inhibitors have been evaluated in phase I-III trials [680] • Elastokine-specific targeting remains preclinical [679]
Fibronectin	• ILT3 blockade has entered phase I-II clinical evaluation [734] • Fibronectin-targeted ILT3 antibody formats remain preclinical [735] • FN-EDB-targeted antibodies and immunocytokines have entered phase I-II clinical testing, but no approved therapies are currently available [736]
Laminins	• TGF-β inhibitor galunisertib reached phase I-II clinical testing and showed immunomodulatory effects but was discontinued and is not approved [737] • 67LR-laminin interaction inhibitors remain preclinical [738] • Laminin-332 has clinical relevance as a prognostic biomarker for chemoresistance [739]
Tenascin C	• EGFRvIII peptide vaccine has reached different stages of clinical testing, including phase III [740] • Randomized phase IIb evaluation for survivin peptide vaccination [740] • Personalized or neoantigen-derived peptide vaccines remain largely phase I/early phase II [741] • Centyrin- and Tencon-based molecules targeting or exploiting TNC sequences remain in early development, from preclinical to phase I [742]
Nidogen/entactin	• NID1 and NID2 remain preclinical and exploratory, with no registered clinical trials for direct targeting to date [681]
Matrix metalloproteinases	• Selective MMP-9 inhibition with andecaliximab entered phase I-III trials in solid tumors, but phase III studies failed to show an overall survival benefit and was discontinued [743] • PAR-2-MMP-2 axis inhibitors remain preclinical. p38 MAPK inhibitors reached phase I-III trials but showed limited efficacy and toxicity concerns [744] • The HSP27-targeting drug apatorsen reached phase II, but no advanced oncology trials are ongoing [745]
Proteoglycans and glycosaminoglycans	• WT1 peptide vaccines have been evaluated in phase I-II glioblastoma trials but no regulatory approval [746] • GPC3 peptide vaccines have reached early-stage clinical testing in hepatocellular carcinoma but are not approved [713] • CSPG4-directed CAR-T cells have entered phase I trials in solid tumors, whereas CSPG4 monoclonal antibodies remain preclinical [747]
Perlecan/HSPG2	• Anti-HSPG2 antibody-based therapy for triple-negative breast cancer remains preclinical [717] • Antisense perlecan and anti-perlecan/LG3 antibody approaches are preclinical proof-of-concept strategies with no clinical development to date [748]
Agrin	• Agrin knockdown, anti-agrin antibodies, and agrin-targeted immunomodulation remain preclinical [701] • LRP4/MUSK targeting in cancer is also preclinical, with no clinical-stage therapeutics currently available [701]
Fibulins	• Targeting fibulin-3/EFEMP1 to inhibit ADAM17-Notch-NF-κB signaling remains preclinical [749] • No phase I or II oncology trials directly targeting fibulin-3 have been reported
Lumican	• The AC117386.2/miR-378c/LUM regulatory axis is supported by preclinical studies, but no therapeutic agents targeting this axis have entered clinical development [750] • Lumican and miRNA signatures are being explored as prognostic or predictive biomarkers [751]
Periostin/POSTN	• No phase I/II oncology trials directly targeting TWIST1 or using RNA interference to suppress periostin are currently available [752] • Indirect strategies aimed at overcoming POSTN-driven immune exclusion, including TGF-β, FAK, CAF, and immunotherapy-based combinations, are being evaluated in phase I-III settings [753]
ECM-bound growth factors	• No clinical trials currently integrate API5 targeting with FGFR1/PKCδ/ERK inhibition [754] • Downstream FGFR1 and ERK pathway components are clinically actionable, with multiple inhibitors approved or under early- to late-phase evaluation[755] • Trastuzumab and rituximab are clinically approved monoclonal antibodies [756] • HER2 antibody-drug conjugates and immune-checkpoint combinations represent next-generation approaches [757]
ADAM metalloproteases	• D8P1C1, an anti-ADAM17 monoclonal antibody for triple-negative breast cancer, remains at the preclinical proof-of-concept stage and has not progressed into clinical development as of 2025 [758]

ADAM, a disintegrin and metalloproteinase; ADAM10, a disintegrin and metalloproteinase domain-containing protein 10; ADAM17, a disintegrin and metalloproteinase domain-containing protein 17; API5, apoptosis inhibitor 5; CAF, cancer-associated fibroblast; CAR-T, chimeric antigen receptor T cell; COL, collagen; COL6A3, collagen type VI α3 chain; COL10A1, collagen type X α1 chain; COL XII, type XII collagen; CSPG4, chondroitin sulfate proteoglycan 4; DDR1, discoidin domain receptor 1; D8P1C1, anti-ADAM17 monoclonal antibody candidate; ECM, extracellular matrix; EDB, extra domain B; EFEMP1, EGF-containing fibulin extracellular matrix protein 1; EGFRVIII, epidermal growth factor receptor variant III; ERK, extracellular signal-regulated kinase; FAK, focal adhesion kinase; FACIT, fibril-associated collagens with interrupted triple helices; Fc, fragment crystallizable; FGFR1, fibroblast growth factor receptor 1; FN, fibronectin; GAG, glycosaminoglycan; GPC3, glypican 3; HER2, human epidermal growth factor receptor 2; HSP27, heat shock protein 27; HSPG2, heparan sulfate proteoglycan 2; ILT3, immunoglobulin-like transcript 3; LAIR-1, leukocyte-associated immunoglobulin-like receptor 1; LAIR-2, leukocyte-associated immunoglobulin-like receptor 2; LUM, lumican; MAPK, mitogen-activated protein kinase; miR-378c, microRNA-378c; MMP, matrix metalloproteinase; MMP-9, matrix metalloproteinase 9; NC410, LAIR-2 Fc fusion protein; NF-κB, nuclear factor κB; NID1, nidogen 1; NID2, nidogen 2; PAR-2, protease-activated receptor 2; PKCδ, protein kinase Cδ; POSTN, periostin; RNA, ribonucleic acid; TGF-β, transforming growth factor β; TNC, tenascin-C; TWIST1, twist family bHLH transcription factor 1; WT1, Wilms tumor 1; 67LR, 67-kDa laminin receptor

Taken together, ECM-targeted therapy represents a promising but complex strategy in cancer treatment. Its major opportunity lies in converting a dense, immune-excluded, therapy-resistant microenvironment into one that is more permeable and immunologically accessible. However, successful translation will require precise control over the extent and location of matrix remodeling to maximize therapeutic benefit while minimizing off-target toxicity, unwanted tissue damage, and prometastatic effects.

### Potential biomarkers within the ECM for predicting disease progression and therapeutic response

ECM is a 3D maze-like structure comprising many macromolecules, including proteins, glycoproteins, polysaccharides, and PGs. ECM also encompasses various regulatory and signaling molecules, including GFs, cytokines, circular RNAs (circRNAs), and miRNAs within TME-associated exosomes [595]. The ECM, collectively referred to as the matrisome, is synthesized by multiple cell types, with fibroblasts serving as the principal contributors [596]. In addition to its structural role, the ECM acts as a dynamic and multifunctional network that governs angiogenesis [87], innate and adaptive immunity [597], and cellular migration [598], thereby profoundly influencing cancer prognosis and treatment responsiveness.

ECM-derived biomarkers for disease progression and therapeutic response can be organized into 4 major categories: molecular markers of matrix composition and remodeling, circulating ECM-derived biomarkers, biophysical and architectural matrix features, and tumor-specific ECM signatures. Molecular ECM biomarkers include matrisome-associated gene signatures, proteolytic ECM fragments, and tumor-associated splice variants [596]. Large-scale matrisome profiling across multiple malignancies has identified tumor-specific ECM signatures with diagnostic and prognostic relevance [599]. In addition, matrikines and collagen-derived fragments, such as tumstatin, PRO-C3 [600], C1M [596], C3M [601], and C4M [600], have emerged as ECM-derived biomarkers linked to tumor progression, survival, and immunotherapy response. Versikine, a versican-derived fragment, has also been associated with T cell infiltration and immunotherapy responsiveness [602]. Similarly, the oncofetal FN splice variants extradomain A (EDA) and EDB are re-expressed in highly remodeled tumor ECM and hold value as markers of malignant progression.

Circulating ECM-derived biomarkers offer a noninvasive means of monitoring stromal remodeling. Soluble ECM molecules and degradation products released into the circulation can be detected in peripheral blood using routine assays and may serve as liquid biopsy markers of tumor burden, progression, and treatment response. It includes proteins such as carcinoembryonic antigen (CEA), nucleic acids such as circulating RNAs, circulating tumor cells, and extracellular vesicles, all of which show strong potential for cancer diagnosis [603–606]. ECM-associated extracellular vesicles and matrix-bound nanovesicles may further provide tumor-specific information related to stromal activity and metastatic potential.

Biophysical and architectural features of the ECM are increasingly recognized as functional biomarkers [607]. Matrix stiffness, collagen density, and fiber organization influence tumor-cell behavior, immune exclusion, and drug sensitivity [599]. For example, increased ECM rigidity in PDAC has been associated with resistance to paclitaxel [608], whereas collagen architecture patterns such as TACS have shown prognostic value in breast cancer, with TACS-3 emerging as an independent predictor of poor outcome [398].

Finally, tumor-specific ECM expression patterns and combinatorial biomarker panels may improve clinical stratification. In gliomas, elevated expression of collagens such as COL3A1, COL4A1, and COL5A2 has been associated with immune suppression and poor prognosis [609]. In breast cancer, increased collagen deposition in triple-negative breast cancer (TNBC) and HER2-positive tumors correlates with invasive behavior, particularly in the presence of CAFs [276,610]. Multi-marker approaches may further enhance diagnostic performance; for instance, combining POSTN with CA15-3 and CEA improves breast cancer detection compared with single-marker approaches [611].

### Clinical validation of emerging ECM-related biomarkers and potential limitations

Emerging ECM-related biomarkers are gaining increasing attention for their potential to inform cancer diagnosis, prognosis, and therapeutic response. By reflecting key features of tumor desmoplasia, matrix remodeling, immune exclusion, and stromal activation, these biomarkers offer a mechanistically relevant window into disease progression. Nevertheless, their successful clinical implementation requires careful validation, as their performance may be influenced by cancer-type specificity, spatial heterogeneity, technical variability, and limited translational standardization.

#### ECM-related biomarkers with emerging clinical relevance

ECM molecules and ECM-remodeling mediators are increasingly recognized as candidate biomarkers of tumor progression, stromal activation, and immune dysfunction within the TME. These include structural ECM components such as collagens, FN, laminins, and HA; PGs such as versican, decorin, biglycan, syndecan-1, glypicans, and perlecan; matricellular proteins including TNC, POSTN, osteopontin, SPARC (secreted protein acidic and rich in cysteine), and thrombospondins; and remodeling enzymes such as MMP-2, MMP-7, MMP-9, MMP-14, LOX/LOXL2, and heparanase [599]. Collectively, these factors reflect active-matrix remodeling during tumor evolution and have been associated with desmoplasia, increased matrix stiffness, altered chemokine sequestration, impaired immune cell trafficking, and immune-excluded tumor phenotypes. As such, ECM-related biomarkers may provide not only prognostic information on invasion and metastasis but also mechanistic insight into how matrix remodeling contributes to immune escape and therapeutic resistance.

#### Biological and clinical constraints on ECM biomarker validation

Despite their promise, the clinical translation of ECM-related biomarkers remains challenging. One major limitation is cancer-type specificity, as patterns of ECM remodeling differ substantially across tumor types and even across molecular subtypes within the same cancer [612]. Biomarkers that are informative in one setting may not retain the same value in another. For example, FN and HA may be more relevant in desmoplastic tumors such as TNBC or pancreatic cancer, whereas other ECM components may dominate in distinct tumor contexts. This variability limits the broad applicability of single ECM biomarkers and underscores the need for tumor-specific validation. ECM composition, stiffness, and remodeling activity can vary markedly between patients and across spatial regions within the same tumor. Consequently, biomarker levels may differ according to sampling site, disease stage, stromal content, or local immune composition [613]. Such heterogeneity complicates interpretation and may reduce reproducibility, particularly when conclusions are drawn from localized tissue biopsies alone [614]. In addition, patient-related variables, including age, sex, and genetic background, may further influence ECM biomarker expression. Another important limitation is biological confounding. Many ECM-associated molecules are not cancer-specific and may also be altered in fibrosis, chronic inflammation, tissue repair, or wound healing. This reduces specificity and increases the risk of false-positive or misleading interpretations when ECM biomarkers are used in isolation. For this reason, single-marker strategies are unlikely to fully capture the complexity of matrix remodeling during tumor progression.

#### Predictive value for therapeutic response: Promise, but limited validation

The ability of ECM-related biomarkers to predict therapeutic response is promising, but current evidence remains uneven across tumor types and treatment settings. Associations between ECM remodeling and treatment resistance are biologically plausible, particularly in the context of matrix stiffening, stromal exclusion, and altered immune cell access. However, for many proposed ECM biomarkers, predictive utility has not yet been established through large prospective studies. Thus, it is important to distinguish prognostic associations from true predictive biomarkers of treatment response [528]. At present, many ECM-related markers should be considered exploratory or emerging, rather than clinically validated predictive tools. Their translational value will depend on whether they can reproducibly stratify patients, outperform or complement existing clinical variables, and demonstrate utility across independent cohorts [615]. A more balanced interpretation is that ECM biomarkers hold substantial translational potential, but most remain at an early or intermediate stage of clinical qualification

#### Methodological and assay-standardization challenges

Methodological limitations further constrain clinical implementation. Detection platforms for ECM-related biomarkers must be sufficiently sensitive, reproducible, and standardized to support routine clinical use. Variability in tissue processing, assay format, analytical thresholds, and sampling strategy can all affect biomarker performance [161]. Moreover, because ECM remodeling is spatially and temporally dynamic, single time-point measurements may provide only a partial view of disease biology [88]. These issues highlight the need for robust assay harmonization and longitudinal validation in well-annotated patient cohorts.

In this context, multi-analyte or matrix-centered biomarker panels may be more informative than single ECM markers. A broader assessment of ECM states, including structural, biochemical, and remodeling-related features, may better reflect tumor behavior than reliance on one protein or one biopsy region alone. Such panel-based approaches could improve sensitivity and biological interpretability, especially when integrated with complementary molecular or spatial data.

#### Translational readiness, regulatory considerations, and future directions

As ECM biomarkers move toward clinical translation, it is essential to distinguish between exploratory candidates, biomarkers supported by retrospective clinical associations, and those approaching formal clinical validation. Most ECM-related biomarkers currently fall within the first 2 categories. Advancing them toward clinical readiness will require standardized assays, independent validation across cancer-specific cohorts, and prospective studies demonstrating added value for diagnosis, prognosis, or treatment selection.

Regulatory and ethical considerations also warrant attention. Biomarker deployment in early detection or treatment stratification must be supported by analytical validity, clinical validity, and clinical utility while also addressing patient privacy, incidental findings, and responsible interpretation. Looking ahead, the most effective translational strategy will likely involve integrating ECM biomarkers with genomic, proteomic, and spatial profiling approaches, together with a deeper mechanistic understanding of ECM remodeling across tumor types. Such efforts may help refine ECM-based biomarker panels and improve their applicability in personalized cancer therapy.

## Recent Revolutions in ECM Targeted Cancer Therapy and Future Perspectives

As ECM-targeted therapies move closer to clinical translation, the most valuable analytical platforms are those that can identify actionable stromal features, guide patient selection, and monitor treatment response. Rather than viewing emerging technologies as standalone innovations, their translational importance lies in how effectively they resolve ECM composition, architecture, and immune contexture within tumors [616]. The ECM functions as both a biochemical and biophysical regulator of immune cell behavior within the TME [88]. Rather than serving as a passive scaffold, it shapes immune cell localization, activation, and functional states through its composition, architecture, stiffness, density, and topography [617]. When aberrantly remodeled, it promotes immune exclusion and stromal immunosuppression by generating collagen-rich, therapy-resistant niches that impair effective antitumor immunity [152]. These effects are reinforced by reciprocal interactions among CAFs, immune cells, and matrix-remodeling programs, which together sustain a tumor-permissive microenvironment and represent important targets for combinatorial therapeutic intervention [425]. Thus, ECM remodeling promotes immune escape through both mechanical obstruction and active stromal reprogramming.

### Single-cell and spatial omics technologies

Conventional bulk tumor profiling has been insufficient to resolve tumor heterogeneity and the spatial organization of the TME, particularly tumor–ECM interactions. In contrast, single-cell multi-omics technologies have become essential for interrogating tumors at cellular resolution across genomic, transcriptomic, proteomic, metabolomic, and epigenomic layers, although tissue dissociation can disrupt spatial context and limit analysis of cell–cell interactions [618]. To overcome this limitation, ST and related imaging-based platforms now provide spatially resolved insights into diverse cell populations, noncellular elements, and secreted mediators within the TME. The integration of scRNA-seq with ST has greatly advanced the study of ECM–immune crosstalk by revealing cellular heterogeneity, lineage plasticity, rare immune subtypes, and the effects of ECM stiffness, density, and topography on transcriptional states. Matsuoka et al. [619] used ST and scRNA-seq to show that pulmonary pleomorphic carcinoma harbors diverse driver alterations, including METex14 and ALK (anaplastic lymphoma kinase) fusions, and identified epithelial cell populations that promote sarcomatoid differentiation through an intermediate EMT state and ECM remodeling, revealing potential therapeutic targets. Another study used the same approach to show that intrahepatic cholangiocarcinoma cells display increased proliferation, immune evasion, and crosstalk with POSTN–fibroblasts and macrophages, identifying potential therapeutic targets. Nerves are integral to tumor biology. Chen et al. [620] used scRNA-seq and ST to identify distinct cellular phenotypes involved in neural invasion in pancreatic carcinoma, including immune–neural interactions marked by NOD-like receptor family pyrin domain containing 3-positive (NLRP3^+^) macrophages and neuropilin 2-positive (NRP2^+^) fibroblasts in high-neural-invasion tissues. They also highlighted transforming growth factor β induced-positive (TGFBI^+^) Schwann cells as promoters of tumor migration and poor survival, providing potential targets for cancer-neural invasion [621].

Laser-capture microdissection sequencing (LCM-seq), in combination with RNA sequencing, is a powerful tool for identifying differentially expressed genes in various tumors [616]. One study developed a multifaceted immunohistochemistry/LCM-seq approach for the ST analysis of immunohistochemically identified neurons using LCM. Using this method, approximately 16,000 transcripts were detected from paraffin-embedded, fluorescently labeled neurons in brain sections of transgenic mice [622].

Additional approaches, including CosMx, 10x Genomics Visium, Slide-seq, Stereo-seq, spatial proteomics platforms such as multiplexed ion beam imaging (MIBI) [623], co-detection by indexing (CODEX) [624], and imaging mass cytometry (IMC) [625], as well as integrated spatial multi-omics methods like assay for transposase-accessible chromatin (ATAC)–RNA-seq and CUT&Tag–RNA-seq, further enable detailed mapping of cellular neighborhoods, intercellular interactions, immune receptor diversity, and clonal heterogeneity within tumors [626]. Spatial T/B cell receptor sequencing has been developed to investigate immune receptor diversity and molecular dynamics in the TME [627]. Paired slide-DNA-seq and slide-RNA-seq enable the understanding of mechanistic relationships across omics and clonal heterogeneity. For instance, Ravi et al. [628] elucidated the transcriptomics, metabolomics, and proteomics in glioblastoma by implanting glioblastoma stem cells into human and rodent neocortical tissue, which critically highlighted the hypoxia-induced glioma ECM architecture, immunological stress, and chromosomal aberrations. Similarly, Ji et al. [629] used scRNA-seq, ST, and MIBI to define the cellular and spatial landscape of human cSCC, identifying 4 tumor subpopulations, including a tumor-specific keratinocyte population enriched in a fibrovascular niche that appears to drive cSCC. Together, these technologies provide a high-resolution framework for understanding the spatial dynamics of ECM-mediated immunosuppression and for identifying clinically relevant stromal and immune niches.

### Artificial intelligence and bioengineering

Artificial intelligence (AI) and bioengineering are emerging as important enablers of ECM-targeted therapeutic strategies. Through integration of digital pathology, second harmonic generation imaging, radiology, and multi-omics data, AI can identify ECM features such as collagen density, fiber alignment, stromal activation, fibrosis, and immune-excluded spatial patterns that are strongly linked to tumor progression and therapeutic response [630]. AI-assisted pathology and spatial analysis, therefore, provide a means to stratify patients, identify matrix-defined therapeutic vulnerabilities, and monitor stromal remodeling during treatment. Recent multimodal studies further highlight this translational potential. For example, Pentimalli et al. [631] integrated CosMx spatial profiling with ECM imaging to generate a high-resolution 3D map of a clinical tumor sample, enabling quantification of collagen and elastin content, reconstruction of multicellular neighborhoods, and identification of fibroblast-associated ECM remodeling states linked to tumor progression and immune escape. Collectively, these advances position AI as a critical bridge between ECM biology and precision therapeutics, supporting biomarker discovery, rational combination design, and more personalized application of ECM-targeted interventions [621]. In parallel, recent multimodal spatial studies have illustrated the translational value of these approaches.

On the bioengineering side, 3D dynamic hydrogels with tunable stiffness, viscoelasticity, and ligand density are being used to recapitulate the biochemical and biomechanical properties of desmoplastic tumors, allowing functional interrogation of tumor–ECM interactions under controlled conditions [632]. In parallel, advances in bioengineering provide unprecedented tools for reconstructing and interrogating tumor–ECM interactions [633,634]. Likewise, laser-capture-based molecular profiling can help interrogate specific ECM-dense regions that may harbor therapy-resistant cell populations [635]. Collectively, these approaches are most relevant when used to link ECM states with therapeutic vulnerability, thereby supporting biomarker discovery, patient stratification, and rational design of ECM-targeted combination therapies [636]. Altogether, these AI-enabled and bioengineered platforms provide a framework for designing therapies that normalize ECM architecture, reprogram stromal behavior, and enhance the efficacy of immunotherapy.

### ECM-targeted therapeutics

ECM-targeted therapeutics aim to reverse the tumor-promoting and immunosuppressive properties of the ECM by altering its composition, architecture, and mechanical features. In principle, these strategies seek to soften fibrotic stroma, reduce matrix crosslinking, degrade excessive ECM components, reprogram stromal cells such as CAFs, and restore immune cell access to tumor nests [637]. Among the most studied approaches are therapies targeting ECM crosslinking and stiffness. Inhibition of LOX family members, particularly LOXL2, has been explored to reduce collagen crosslinking and stromal rigidity [638]. Preclinical studies with the anti-LOXL2 antibody AB0023 showed reduced ECM crosslinking, impaired tumor growth, and modulation of the TME in mouse xenograft models [639]. However, the humanized counterpart simtuzumab (AB0024/GS-6624) failed to show meaningful clinical benefit in phase II trials in colorectal and pancreatic adenocarcinoma, underscoring the difficulty of translating ECM-normalizing strategies from preclinical models into effective cancer therapies in patients [640,641]. Another major strategy involves enzymatic degradation of HA-rich matrix to improve perfusion and drug penetration [642]. The PEGylated recombinant hyaluronidase, PEGPH20, enhanced the permeability and efficacy of agents such as doxorubicin and gemcitabine in preclinical pancreatic cancer models and improved survival relative to chemotherapy alone [642]. Yet clinical outcomes were disappointing [643,644]. Although the phase I/II study (NCT01839487) suggested benefit in patients with high pretreatment HA levels, the phase III trial (NCT02715804), which evaluated PEGPH20 in combination with gemcitabine and nab-paclitaxel, was terminated because it did not improve overall survival, progression-free survival, or duration of response. Consequently, clinical development of PEGPH20 was discontinued by Halozyme Therapeutics. This failure likely reflects the biological complexity of pancreatic cancer, including inadequate patient stratification and the limitations of nonselective ECM depletion, which may also disrupt vascular integrity and immune cell trafficking. The failure of PEGPH20 in pancreatic cancer appears to be multi-factorial, rooted in the complex biology of the PDAC TME [224]. Clinical studies suggest that depletion of HA alone is not enough to reverse the complex and multifactorial mechanisms driving chemoresistance in pancreatic tumors. Reported reasons for the lack of clinical benefit include inadequate patient stratification according to intratumoral HA levels and nonselective ECM degradation, which may adversely affect vascular integrity and immune cell trafficking. In 2019, a similar study assessed the efficacy of PEGPH20 combined with modified FOLFIRINOX, a chemotherapy regimen consisting of fluorouracil, leucovorin, irinotecan, and oxaliplatin, in a randomized clinical trial [645].

Another promising strategy involves the combined inhibition of multiple immune checkpoints, including PD-1, TIM-3, and LAG-3, which are frequently exploited by tumors to drive T cell dysfunction and exhaustion [646]. Recent preclinical and early-phase clinical trials demonstrate that dual or multi-ICI therapy can enhance antitumor immune responses, particularly when coupled with strategies that normalize ECM architecture [647]. Furthermore, miRNA-based therapeutic approaches hold potential for reprogramming the immune and stromal components of the TME. For instance, miR-138 has been shown to regulate CTLA-4 and PD-1 expression in T cells, while miR-34a suppresses PD-L1 in tumor cells, together contributing to the restoration of cytotoxic T cell function [648]. Similarly, upstream modulators such as PTEN and CDK5 can be targeted to overcome immune resistance, thus enhancing the durability of antitumor immunity [649]. Taken together, the future of cancer immunotherapy is likely to rely on an integrated approach that combines molecular targeting, immune modulation, and ECM remodeling. Far from being a passive bystander, the ECM is a central regulator of tumor biology, and therapeutically targeting its dynamic properties may provide one of the most promising paths toward durable, personalized, and transformative cancer treatment.

## Conclusion

A prominent hallmark of cancer cells is their ability to evade the host’s adaptive immune response mechanism and maintain their immortality, a key feature that forms the basis of immunotherapy. It has been known that cancer cells have developed an elaborate maneuver not to fight, but to escape and evade the physiological immune system, thereby minimizing their own costs, while tactically evolving by simultaneously changing their genotype and phenotype. ECM and ECM components play a key regulatory role in immune evasion mechanisms, which are robustly addressed in this review. The dynamic nature of these ECM proteins, ECM remodeling, signaling cascades, and disrupted cell–ECM mechano-reciprocity all contribute to these factors. Despite ongoing efforts to develop effective strategies against immune evasion, heterogeneous treatment responses highlight the need for personalized tumor profiling to define the immunological, immunogenetic, and signaling alterations that drive immune escape. Although immune checkpoint-based cancer immunotherapy has shown great promise in treating various types of cancer, only a small percentage of patients experience therapeutic benefits. Therefore, immunotherapy should be enhanced by combining conventional treatment strategies with personalized approaches to achieve a holistic and synergistic outcome. Indeed, the spatial heterogeneity and dynamics of the tumor ECM and TME composition in various solid tumors seem to be major challenges in designing effective therapeutic strategies. Hence, a better understanding and deeper characterization of the tumor ECM components, their specific interactions, and protumorigenic signal transduction mechanisms in an immune-excluded phenotype is essential for the futuristic therapeutic scenario. This review identifies the ECM as a central active regulator of tumor immune evasion, rather than a passive structural scaffold. Across solid tumors, ECM remodeling, abnormal stiffening, altered composition, and dysregulated cell–ECM mechanosignaling collectively restrict immune cell infiltration, promote T cell dysfunction, support immunosuppressive stromal and myeloid programs, and reduce responsiveness to immunotherapy. A major translational implication is that effective cancer immunotherapy will likely require combination strategies that integrate immune modulation with ECM-targeted and patient-specific approaches. Moving forward, deeper spatial and mechanistic characterization of tumor-specific ECM states will be essential for improving biomarker-guided stratification and developing more durable, personalized therapies.
